# Antimicrobial susceptibility and virulence genes of clinical and environmental isolates of *Pseudomonas aeruginosa*

**DOI:** 10.7717/peerj.6217

**Published:** 2019-01-22

**Authors:** Siew Mun Liew, Ganeswrei Rajasekaram, SD Ampalam Puthucheary, Kek Heng Chua

**Affiliations:** 1 Department of Biomedical Science, Faculty of Medicine, University of Malaya, Kuala Lumpur, Malaysia; 2 Department of Pathology, Hospital Sultanah Aminah, Johor Bahru, Johor, Malaysia

**Keywords:** Antimicrobial susceptibility, Virulence, *Pseudomonas aeruginosa*

## Abstract

**Background:**

* Pseudomonas aeruginosa* is ubiquitous, has intrinsic antibiotic resistance mechanisms, and is associated with serious hospital-associated infections. It has evolved from being a burn wound infection into a major nosocomial threat. In this study, we compared and correlated the antimicrobial resistance, virulence traits and clonal relatedness between clinical and fresh water environmental isolates of *P. aeruginosa*.

**Methods:**

219 *P. aeruginosa* isolates were studied: (a) 105 clinical isolates from 1977 to 1985 (*n* = 52) and 2015 (*n* = 53), and (b) 114 environmental isolates from different fresh water sources. All isolates were subjected to ERIC-PCR typing, antimicrobial susceptibility testing and virulence factor genes screening.

**Results:**

Clinical and environmental isolates of *P. aeruginosa* were genetically heterogenous, with only four clinical isolates showing 100% identical ERIC-PCR patterns to seven environmental isolates. Most of the clinical and environmental isolates were sensitive to almost all of the antipseudomonal drugs, except for ticarcillin/clavulanic acid. Increased resistant isolates was seen in 2015 compared to that of the archived isolates; four MDR strains were detected and all were retrieved in 2015. All clinical isolates retrieved from 1977 to 1985 were susceptible to ceftazidime and ciprofloxacin; but in comparison, the clinical isolates recovered in 2015 exhibited 9.4% resistance to ceftazidime and 5.7% to ciprofloxacin; a rise in resistance to imipenem (3.8% to 7.5%), piperacillin (9.6% to 11.3%) and amikacin (1.9% to 5.7%) and a slight drop in resistance rates to piperacillin/tazobactam (7.7% to 7.5%), ticarcillin/clavulanic acid (19.2% to 18.9%), meropenem (15.4% to 7.5%), doripenem (11.5% to 7.5%), gentamicin (7.7% to 7.5%) and netilmicin (7.7% to 7.5%). Environmental isolates were resistant to piperacillin/tazobactam (1.8%), ciprofloxacin (1.8%), piperacillin (4.4%) and carbapenems (doripenem 11.4%, meropenem 8.8% and imipenem 2.6%). Both clinical and environmental isolates showed high prevalence of virulence factor genes, but none were detected in 10 (9.5%) clinical and 18 (15.8%) environmental isolates. The *exoT* gene was not detected in any of the clinical isolates. Resistance to carbapenems (meropenem, doripenem and imipenem), β-lactamase inhibitors (ticarcillin/clavulanic acid and piperacillin/tazobactam), piperacillin, ceftazidime and ciprofloxacin was observed in some of the isolates without virulence factor genes. Five virulence-negative isolates were susceptible to all of the antimicrobials. Only one MDR strain harbored none of the virulence factor genes.

**Conclusion:**

Over a period of 30 years, a rise in antipseudomonal drug resistance particularly to ceftazidime and ciprofloxacin was observed in two hospitals in Malaysia. The occurrence of resistant environmental isolates from densely populated areas is relevant and gives rise to collective anxiety to the community at large*.*

## Introduction

*Pseudomonas aeruginosa* is an environmental saprophyte, and an opportunistic pathogen affecting mainly immunocompromised patients. Pseudomonal infections include otitis externa (swimmer’s ear), otitis media, folliculitis (hot tub rash), keratitis, soft tissue infections (burn wounds, post-surgical), diabetic foot infections, urinary tract infections, bacteraemia and pneumonia in cystic fibrosis (CF) patients (Gellatly & Hancock, 2013; Lyczak, Cannon & Pier, 2000).

*P. aeruginosa* is considered as one of the harmless bacterial skin flora (Cogen, Nizet & Gallo, 2008). However, once inside the host, depending on the route of entry, it may express a series of pathogenic mechanisms. Flagella, pili and lipopolysaccharide are responsible for bacterial motility and adhesion; type I, II and III secretion systems, phenazine system and lectins are responsible for invasion and dissemination; latency and antimicrobial resistance are due to quorum-sensing and biofilm formation (Kipnis, Sawa & Wiener-Kronish, 2006).

Antibiotic resistance constitutes one of the most serious threats to the global public health and impacts all aspects of therapeutics, animal husbandry and agriculture; it is natural, ancient, and hard wired in the microbial pan-genome (Bhullar et al., 2012). Since the discovery of penicillin (Fleming, 2001), various natural and synthetic antimicrobials have been developed (Bassetti et al., 2013), but the rapid emergence of resistant bacteria in contrast to the slow development of drugs has resulted in a nearly “empty” antibiotic pipeline (Spellberg et al., 2008). Approximately 8% of all healthcare-associated infections in the USA are caused by *P. aeruginosa*, and 13% of them were found to be multidrug resistant (MDR) (CDC, 2013).

*P. aeruginosa* is intrinsically resistant to many antimicrobials (Wroblewska, 2006) due to its low outer membrane permeability which is 100-fold less than *Escherichia coli* (Angus et al., 1982). Selective pressure due to antipseudomonal therapy and especially the use of imipenem has resulted in significantly higher risk of emergence of resistance than the use of ciprofloxacin or piperacillin, but ceftazidime had the lowest risk (Carmeli et al., 1999). Mechanisms responsible for the natural resistance of *P. aeruginosa* are: (i) efflux pumps, (ii) AmpC β-lactamase, (iii) loss of OprD porin and iv) mutations in the topoisomerase II and IV genes (Livermore, 2002), as well as acquired resistance due to aminoglycoside-modifying enzymes (Poole, 2005) and β-lactamases (class A, B and D) (Potron, Poirel & Nordmann, 2015).

The objectives of this study were to (a) determine the clonal relatedness of clinical and environmental isolates of *P. aeruginosa*, (b) test the antimicrobial susceptibility of clinical isolates from different isolation periods: 1977 to 1985 and 2015, (c) evaluate the antimicrobial susceptibility of environmental isolates recovered from fresh water sources in Malaysia in 2015, and (d) investigate the prevalence of virulence factor genes in both clinical and environmental isolates.

## Material and Methods

### Bacterial isolates

One hundred and five non-duplicate clinical isolates of *P. aeruginosa* were obtained from 2 hospitals at different isolation periods: 52 isolates, 1977 to 1985 from the University Malaya Medical Centre (UMMC), Kuala Lumpur; and 53 from Hospital Sultanah Aminah (HSA), Johor Bahru (southern Malaysia) in 2015. One hundred and fourteen environmental isolates recovered in 2015 from different fresh water sources (ponds, waterfall, drains, well water, pools, paddy field and lakes) (Supplemental Information 1).

All isolates were confirmed as *P. aeruginosa* by species-specific PCR (Spilker et al., 2004), grown in Luria-Bertani (LB) broth (Difco, USA) and stored in LB broth with 20% (vol/vol) glycerol at −70 °C.

### ERIC-PCR typing

Genomic DNA of the bacteria was extracted based on the approach as described previously (Teh, Chua & Thong, 2010). Strain diversity was determined by ERIC-PCR using primers as described (Versalovic, Koeuth & Lupski, 1991). PCR was performed in a final volume of 25 µl reaction mixture containing sterile MilliQ water, 4 µl 10 × DreamTaq buffer, 0.25 mM dNTP mix, 50 pmol of each primers, 3.75 mM MgCl_2_, 2 U of Taq polymerase (Thermo Scientific, US) and 100 ng of template DNA. The reaction mixture was denatured at 95 °C for 7 min, and then subjected to 30 cycles at 90 °C for 30 s, annealing at 52 °C for 1 min, extension at 65 °C for 8 min and a final extension at 65 °C for 16 min (Szczuka & Kaznowski, 2004). *P. aeruginosa* PAO1 and *P. aeruginosa* ATCC^^®^^ 27853 were used as controls.

Fingerprint analysis was carried out by the GelJ software (Heras et al., 2015) and similarity was calculated by the Dice coefficient and cluster analysis using the unweighted pair group method with average linkages (UPGMA).

### Antimicrobial susceptibility

Susceptibility of *P. aeruginosa* to the following antimicrobials was performed by the disk diffusion method, according to the Clinical and Laboratory Standards Institute (CLSI) M100-S26 guidelines: piperacillin/tazobactam (100/10 µg), ticarcillin/clavulanic acid (75/10 µg), ceftazidime (30 µg), imipenem (10 µg), meropenem (10 µg), doripenem (10 µg), piperacillin (100 µg), ciprofloxacin (5 µg), amikacin (30 µg), gentamicin (10 µg) and netilmicin (30 µg). MDR was defined as non-susceptible to at least one antimicrobial agent in three or more antimicrobial categories (Magiorakos et al., 2012). *P. aeruginosa* ATCC^®^27853 and *Escherichia coli* ATCC^^®^^ 35218 were used as controls.

### Screening of virulence factors

*P. aeruginosa* virulence factor genes *apr* (alkaline protease), *lasB* (elastase), *phzI*, *phzII*, *phzH*, *phzM*, *phzS* (phenazine precursors), *exoS*, *exoT*, *exoU*, *exoY* (type III secretion system (T3SS) effector enzymes), *pilB* (pili), *pvdA* (pyoverdine), *lecA* and *lecB* (lectins) were chosen based on previous studies (Bradbury et al., 2010; Chemani et al., 2009; Finnan et al., 2004) (Table 1).

**Table 1:** Distribution of virulence factor genes in clinical (1977 to 1985 and 2015) and environmental isolates of *P. aeruginosa*.

Isolates	No. of isolates with virulence genes (%)														
	**Alkaline protease**	**Elastase**	**Phenazine precursors**					**T3SS**				**Pyoverdine**	**Pili**	**Lectins**	
	***apr***	***lasB***^a^	***phzI***^a^	***phzII***^b^	***phzH***^a^	***phzM***	***phzS***^a^	***exoS***^c^	***exoT***^c^	***exoU***^c^	***exoY***^c^	***pvdA***^a^	***pilB***	***lecA***	***lecB***^a^
Clinical (*n* = 105)															
(a) 1977–1985 (*n* = 52)	41 (78.8)	41 (78.8)	41 (78.8)	38 (73.1)	41 (78.8)	33 (63.5)	41 (78.8)	26 (50.0)	0 (0)	17 (32.7)	35 (67.3)	25 (48.1)	4 (7.7)	41 (78.8)	25 (48.1)
(b) 2015 (*n* = 53)	49 (92.5)	51 (96.2)	51 (96.2)	50 (94.3)	51 (96.2)	32 (60.4)	51 (96.2)	39 (73.6)	0 (0)	13 (24.5)	51 (96.2)	37 (69.8)	5 (9.4)	51 (96.2)	37 (69.8)
Environmental (*n* = 114)															
Fresh water	94 (82.5)	95 (83.3)	96 (84.2)	79 (69.3)	94 (82.5)	85 (74.6)	93 (81.6)	91 (79.8)	58 (50.9)	9 (7.9)	93 (81.6)	53 (46.5)	2 (1.8)	94 (82.5)	81 (71.1)

**Notes.**

Significant difference in the prevalence of virulence factor genes a *p* < 0.05 b *p* < 0.01 c *p* < 0.001

PCR was carried out in a reaction mixture containing 1 × reaction buffer, 0.15–0.25 mM dNTP mix, 125–250 pmol of each primers, 0.5–1 U of DreamTaq polymerase (Thermo Scientific, Waltham, MA, USA), 10 ng of template DNA, and made up to 20 µl with sterile MilliQ water. The cycling conditions were: initial denaturation at 96 °C for 5 min, followed by 25–40 cycles at 94 °C for 30 s, 30 s of annealing for *phzI* and *phzII* was at 55 °C; for *apr*, *lasB*, *phzH*, *exoS*, *exoT*, *pilB*, *phzM*, *pvdA*, *lecA* and *lecB* was at 60 °C; for *exoU* and *exoY* was at 61 °C; and for *phzS* was at 65 °C, 1 min of extension at 72 °C and a final extension at 72 °C for 5 min. The products were purified by GeneAll Expin Combo™ GP kit (GeneAll Biotechnology Co. Ltd., Seoul, South Korea) and sent to First BASE Laboratories Sdn Bhd, Malaysia for sequencing. The obtained sequences were compared with sequences available in the National Center for Biotechnology Information (NCBI) database by BLAST search (https://blast.ncbi.nlm.nih.gov/Blast.cgi?PAGE_TYPE=BlastSearch).

### Statistical analysis

Statistical analysis was carried out using the Minitab 18 software (http://www.minitab.com) and the Social Science Statistics website (http://www.socscistatistics.com/tests/ztest/Default2.aspx). A chi-square test was performed to compare the prevalence of antimicrobial resistance and virulence factor genes in all isolates. The calculations were carried out at 95% confidence interval and a *p* < 0.05 considered statistically significant.

## Results

### ERIC-PCR typing

ERIC-PCR fingerprints showed genetic diversity of 50% similarity in clinical (isolation periods of 1977 to 1985 and 2015) and environmental isolates (Supplemental Information 2). However, four clinical strains (J43, x117, PA37 and PA40) had 100% identical ERIC-PCR patterns to seven environmental isolates (UW21, F9, UW7, UW12, UB30, TL3 and UB21).

**Figure 1:**
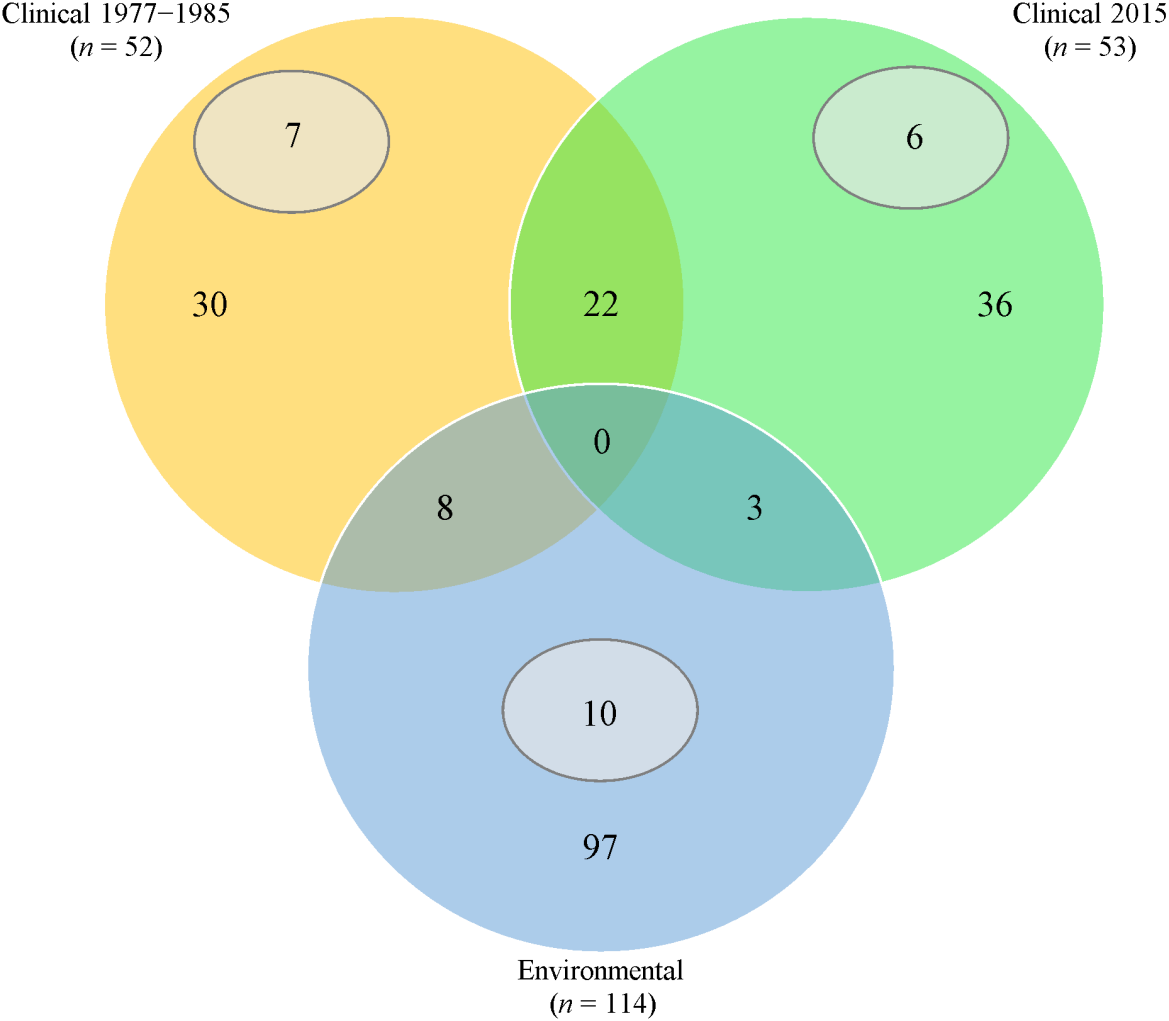
Venn diagram of ERIC-PCR patterns. Orange/Green/Blue sections and intersections represent the number of isolates with distinct and similar ERIC-PCR fingerprints, respectively. Subsets (grey) indicates the number of isolates with 100% clonality in the respective category.

Thirty (57.7%) from 1977 to 1985 and 36 (67.9%) from 2015 clinical isolates had fingerprints that were not seen in the rest of the isolates (Fig. 1). Twenty-two clinical isolates had common ERIC-PCR patterns.

Extensive heterogeneity, in comparison with clinical isolates, was observed in *P. aeruginosa* isolated from fresh water sources; 97 (85.1%) had distinct ERIC-PCR patterns. However, 10 isolates from different geographical areas exhibited 100% identical patterns.

Majority of archived (1977 to 1985) isolates were recovered from swabs (ear, nasal and wound) (38.5%) and urine (26.5%); whilst the recent isolates were from urine (32.1%), bronchial aspirate (26.4%) and tissues (24.5%). However, information of nine (17.4%) archived isolates was not available (Supplemental Information 3).

### Antimicrobial susceptibility

**Figure 2:**
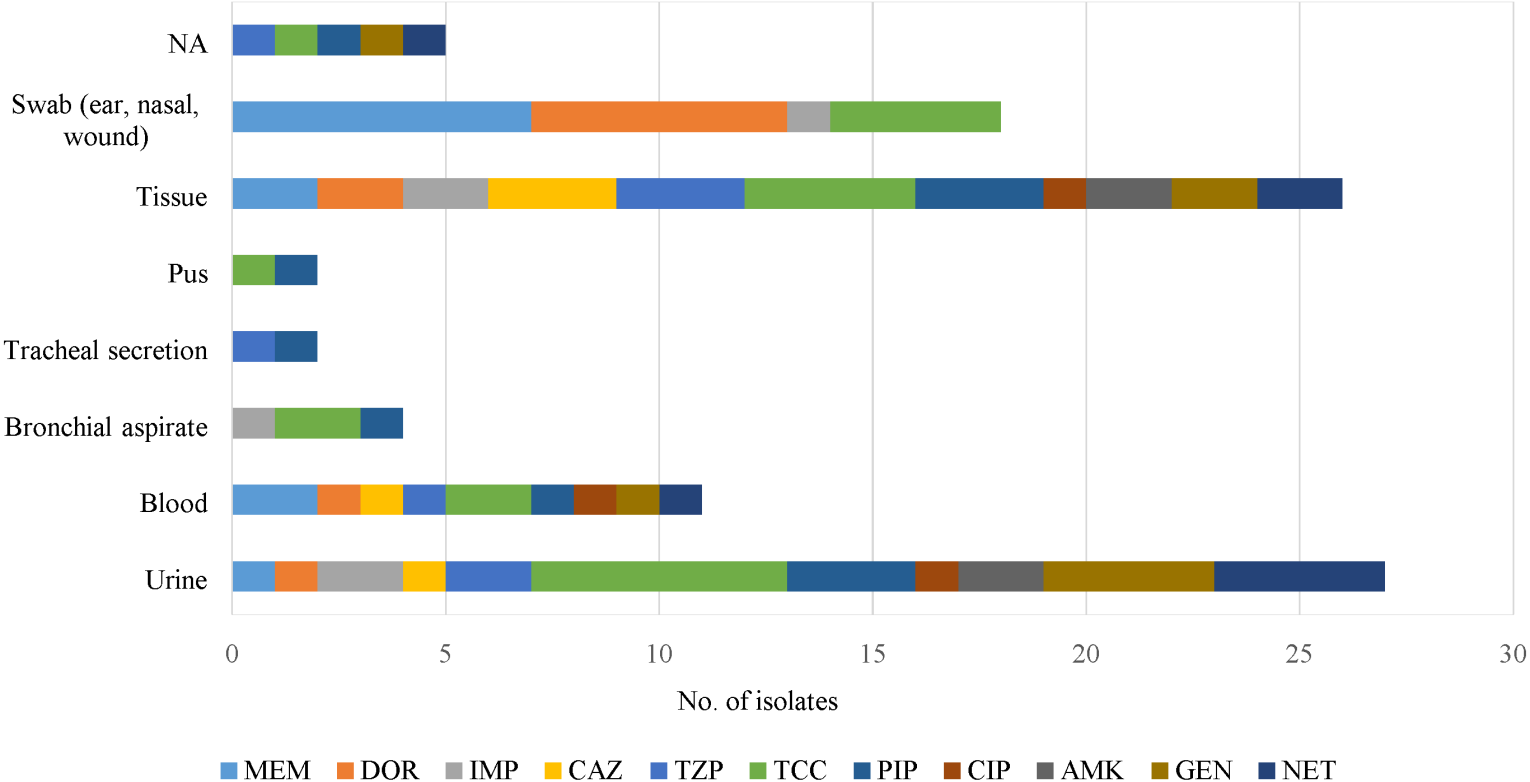
An overview of antimicrobial resistance of *P. aeruginosa* in clinical specimens from two isolation periods: archive (1977 to 1985) and 2015. No antimicrobial resistance was found in the following specimen categories: sputum, cerebrospinal fluid (CSF), slough, peritoneal fluid and discharge/drainage (ear, eye). NA indicates source of specimen is not available.

Antipseudomonal drugs were active against clinical isolates from sputum, cerebrospinal fluid (CSF), slough, peritoneal fluid and discharge/drainage (ear, eye) samples (Fig. 2). However, those from tissue and urine were resistant to all of the antimicrobials, but blood samples were sensitive to imipenem and amikacin.

**Figure 3:**
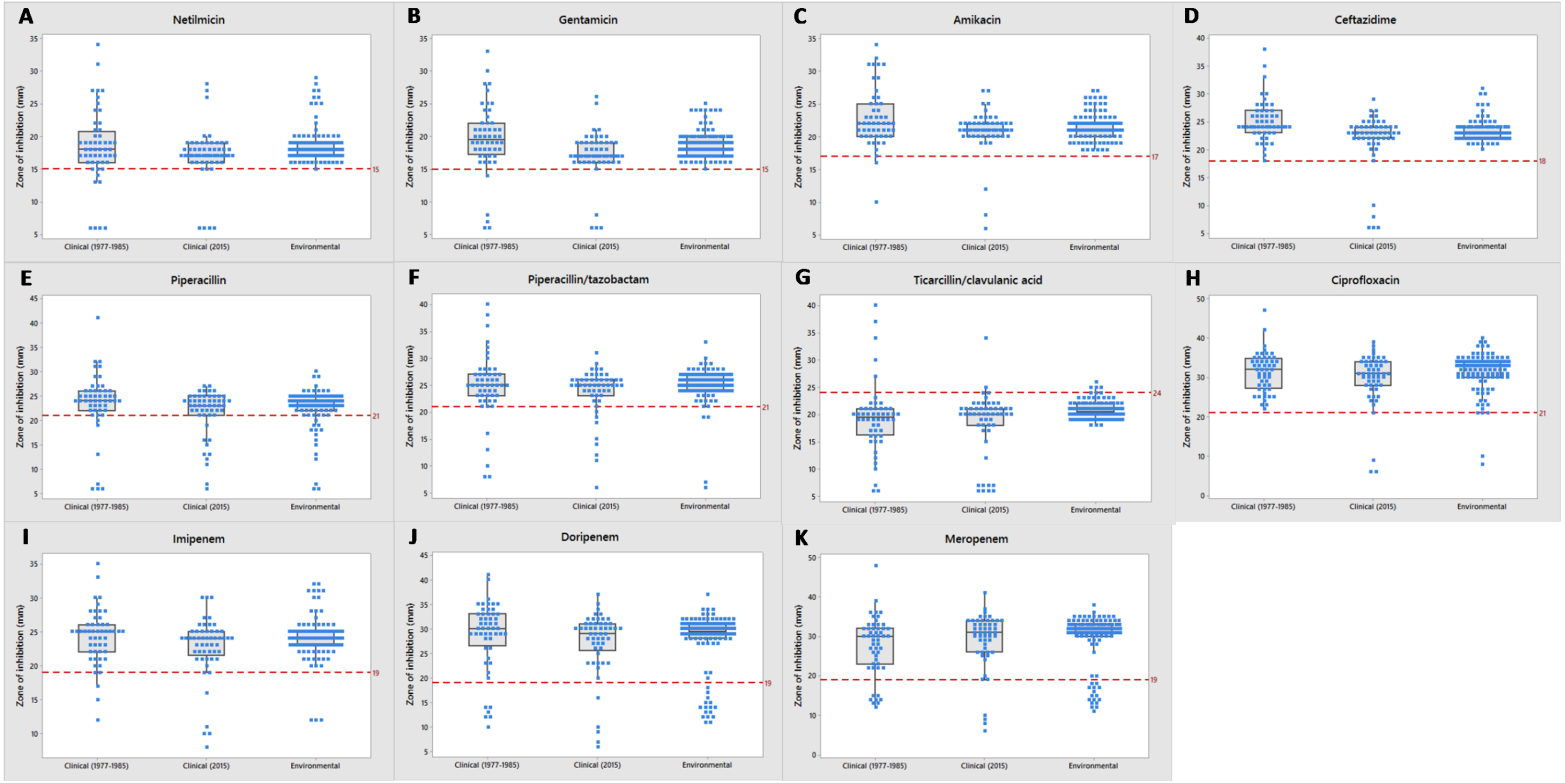
Tendency of antimicrobial susceptibility. Overview of susceptibility patterns of *P. aeruginosa* clinical (1977 to 1985 and 2015) and environmental isolates to the following antimicrobials: (A) netilmicin, (B) gentamicin, (C) amikacin, (D) ceftazidime, (E) piperacillin, (F) piperacillin/tazobactam, (G) ticarcillin/clavulanic acid, (H) ciprofloxacin, (I) imipenem, (J) doripenem and (K) meropenem. The line in each box indicates the median (Q2), the top and bottom lines are the 75th (Q3) and 25th (Q1) percentiles, respectively. The red line represents cut-off point of susceptibility as defined by the CLSI M100-S26 guidelines. Zone of inhibition (mm) above and below the red line indicates susceptibility and non-susceptibility, respectively.

Most of the clinical and environmental isolates were sensitive to almost all of the antipseudomonal drugs (above red line), except for ticarcillin/clavulanic acid (Fig. 3). Increased resistant isolates (below red line) was seen in 2015 compared to that of the archived isolates. Four MDR strains (J3, J11, J20 and J25) were detected, all were from 2015 clinical sources (Supplemental Information 1).

Environmental isolates from fresh water exhibited consistent susceptibility to almost all of the antimicrobials. However, some were non-susceptible to ciprofloxacin, piperacillin, carbapenems (doripenem, meropenem and imipenem) and piperacillin/tazobactam; almost all (*n* = 110) were non-susceptible to ticarcillin/clavulanic acid.

**Figure 4:**
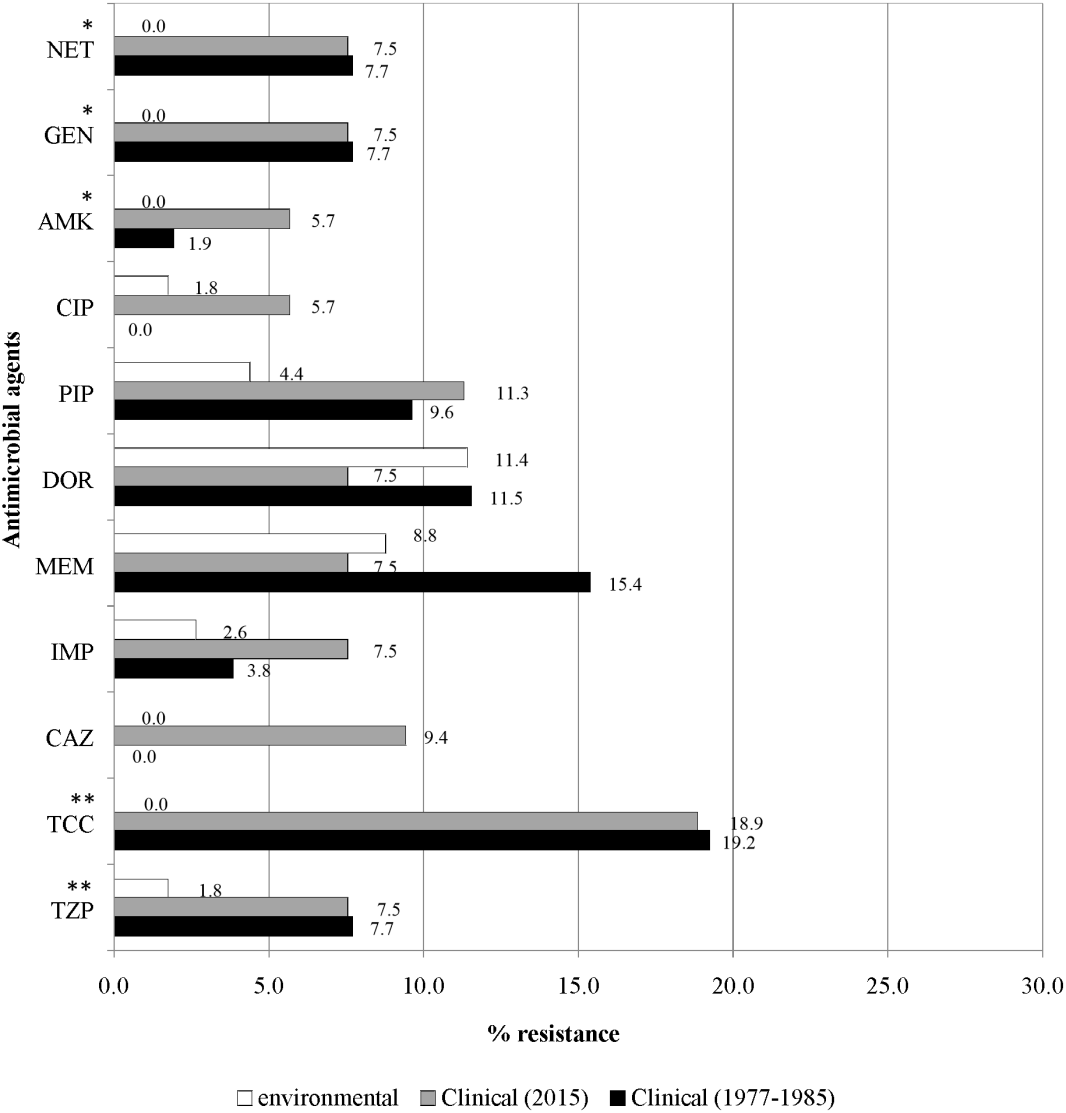
Prevalence of antimicrobial resistance. The symbols * and ** indicate significant levels of *p* < 0.05 and *p* < 0.001, respectively. Notes: NET, netilmicin; GEN, gentamicin; AMK, amikacin; CIP, ciprofloxacin; PIP, piperacillin; DOR, doripenem; MEM, meropenem; IMP, imipenem; CAZ, ceftazidime; TCC, ticarcillin/clavulanic acid; TZP, piperacillin/tazobactam.

Figure 4 illustrates the overall antimicrobial resistance patterns of both clinical and environmental isolates of *P. aeruginosa.* All archived isolates were susceptible (100%) to ceftazidime and ciprofloxacin; but in comparison, the clinical isolates of 2015 exhibited 9.4% resistance to ceftazidime and 5.7% to ciprofloxacin; imipenem resistance rise from 3.8% to 7.5%, piperacillin from 9.6% to 11.3% and amikacin from 1.9% to 5.7%. A slight drop in resistance rates was observed in the recent isolates: piperacillin/tazobactam (7.7% to 7.5%), ticarcillin/clavulanic acid (19.2% to 18.9%), meropenem (15.4% to 7.5%), doripenem (11.5% to 7.5%), gentamicin (7.7% to 7.5%) and netilmicin (7.7% to 7.5%).

Fresh water isolates of 2015 exhibited 100% susceptibility to ceftazidime and the aminoglycosides (amikacin, gentamicin and netilmicin), but were resistant to piperacillin/tazobactam (1.8%) and ciprofloxacin (1.8%), with relatively higher resistance rates to piperacillin (4.4%) and carbapenems (doripenem 11.4%, meropenem 8.8% and imipenem 2.6%).

### Virulence factor genes

Both clinical and environmental isolates of *P. aeruginosa* showed high (>60%) prevalence of virulence factor genes, except for *exoT*, *exoU*, *pvdA* and *pilB* (Table 1). None of the clinical isolates from both isolation periods harbored the *exoT* gene (*p* < 0.001). No virulence factor genes were present in ten (9.5%) clinical and eighteen (15.8%) environmental isolates (Supplemental Information 1).

**Figure 5:**
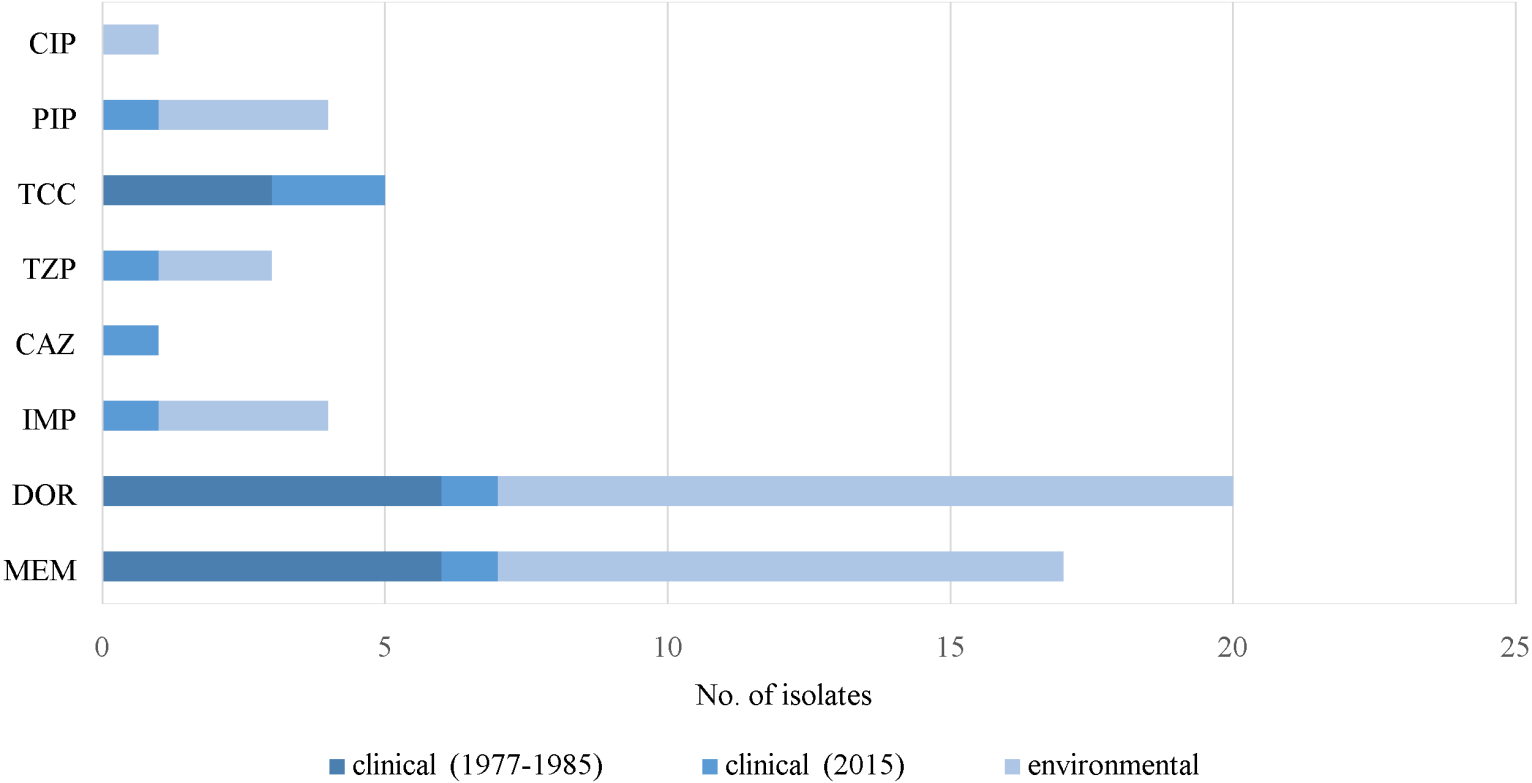
Prevalence of resistance in *P. aeruginosa.* without virulence factor genes. Notes: CIP, ciprofloxacin; PIP, piperacillin; DOR, doripenem; MEM, meropenem; IMP, imipenem; CAZ, ceftazidime; TCC, ticarcillin/clavulanic acid; TZP, piperacillin/tazobactam.

Resistance to carbapenems (meropenem, doripenem and imipenem), β-lactamase inhibitors (ticarcillin/clavulanic acid and piperacillin/tazobactam), piperacillin, ceftazidime and ciprofloxacin was observed in some of the virulence-negative isolates (Fig. 5). Five isolates without virulence factor genes were susceptible to all of the antimicrobials. Only one MDR strain (J3) was absent of any of the virulence factor genes (Supplemental Information 1).

## Discussion

We found high genetic heterogenicity in both categories of isolates as only four clinical strains (J43, x117, PA37 and PA40) displayed 100% clonality to seven environmental isolates, reflecting previous studies where *P. aeruginosa* clinical isolates harboured unique genotypes with low genetic similarity to environmental isolates (Martins et al., 2014; Tumeo et al., 2008). The difference in genetic makeup is probably due to various *P. aeruginosa* biotypes existing in nature, and only those with high adaptability can survive in wide ranging habitats.

Three of the four clonal clinical strains, i.e., PA37, PA40 and x117 were recovered more than 30 years ago indicating that some extraordinary genotypes can persist in the environment for many years and become transmissible. For example, PA14 of sequence type (ST)-253 isolated in the USA 15 years ago, became globally distributed and was found in Queensland, Australia (Kidd et al., 2012).

Aquatic habitats could be a source of pseudomonal infections in humans, as our findings are consistent with reports of environmental isolates exhibited similar genotypes to clinical isolates (Kidd et al., 2012; Pellett, Bigley & Grimes, 1983; Romling et al., 1994).

*P. aeruginosa* is a common cause of healthcare-associated infections such as bloodstream infections, urinary tract infections, surgical site infections and pneumonias especially in CF patients (CDC, 2013). Current antipseudomonal drugs were introduced in 1960s and since then, only few new drugs have been approved for clinical use (Bassetti et al., 2013; Monnet & Giesecke, 2014). Our clinical isolates recovered from 1977 to 1985 exhibited relatively low resistance to amikacin and imipenem but were totally sensitive to ceftazidime and ciprofloxacin probably due to absence or low usage in treatment. Later isolates from the same hospital in 2005 exhibited higher resistance rates than our archived isolates: piperacillin/tazobactam (9.4%), imipenem (9.9%), amikacin (6.73%), gentamicin (12.9%), netilmicin (10.1%), ciprofloxacin (11.3%) and ceftazidime (10.9%) (Raja & Singh, 2007). High resistance rates to these drugs was also documented from Malaysia (Pathmanathan, Samat & Mohamed, 2009). In general, over a period of 30 years (1977 to 2009) there has been a rise in resistance to the core antipseudomonal drugs in Malaysian isolates probably due to selection pressure. The increased resistance to ceftazidime (10.9%) and ciprofloxacin (11.3%) pose a public health challenge. The National Surveillance of Antibiotic Resistance (NSAR) by the Ministry of Health Malaysia (2015) reported that the resistance patterns of *P. aeruginosa* clinical isolates were considerably stable from 2013 to 2015, with a slight decrease in resistance to most of the antipseudomonal drugs but a slight increase in piperacillin/tazobactam resistance (from 4.6% to 5.6%) (Ministry of Health Malaysia, 2015); this was similar to our clinical isolates recovered in 2015, probably due to effective surveillance program.

Some of our aquatic isolates were resistant to ciprofloxacin, piperacillin/tazobactam, piperacillin and carbapenems (imipenem; meropenem; doripenem) which is unusual. A recent study reported 100% antimicrobial susceptibility in *P. aeruginosa* isolated from water samples; however, resistance to meropenem (30.4%), piperacillin/tazobactam (10.6%) and ceftazidime (4.2%) was observed in other *Pseudomonas* spp. isolated from the same sampling points (Kittinger et al., 2016). Another recent study on aquatic isolates of *P. aeruginosa* showed resistance to imipenem (9.43%), ticarcillin/clavulanic acid (1.88%) and co-resistance to piperacillin and ticarcillin/clavulanic acid (1.88%) (Schiavano et al., 2017).

Antibiotic biosynthesis and resistance is believed to be ancient and occurred naturally even before the introduction of antibiotics (Barlow & Hall, 2002; D’Costa et al., 2011). Bacteria isolated from the ancient Lechuguilla Cave of four million years showed most to be multidrug resistant to natural antibiotics. Physiological changes such as the production of antimicrobials occur in these bacteria under nutrient-limited cave environment and bacteria develop resistance as a defence mechanism (Bhullar et al., 2012). The plasmid-mediated quinolone resistance determinant (Qnr) occurs naturally in aquatic reservoirs, and probably enables gene transfer between different waterborne bacteria in habitats where quinolones are not present (Poirel et al., 2005). *P. aeruginosa* possesses inherent resistance to many classes of drugs attributed to the chromosomal-encoded AmpC β-lactamases and efflux pumps, and its lower membrane permeability (Masuda et al., 2000; Poole & Srikumar, 2001). We believe that our resistant environmental isolates had probably acquired resistance in order to survive and persist in diverse natural habitats.

Soil samples from the Netherlands, spanning pre- and post-antibiotic eras (1940 to 2008) had shown increased antibiotic resistance genes in the recent soil samples, with some being more than 15 times more abundant than those in the 1970s (Knapp et al., 2010). The utilization of non-degradable synthetic antibiotics (e.g., quinolones) in aquaculture, extensive use of antibiotics in livestock, broken sewage pipes, hospital effluents and runoff from farms fertilized with livestock faeces, may contribute to the selection of resistant bacteria in natural habitats such as surface waters, ground water, drinking water or sediments (Bartlett, Gilbert & Spellberg, 2013; Goni-Urriza et al., 2000; Kummerer, 2004).

Increased concentration of antibiotics in the environments due to extensive use in clinical and agricultural settings affects the evolution of bacterial resistance and virulence. The interplay between resistance and virulence is postulated to follow a Darwinian model, in which more resistant and virulent isolates will be selected in the population (Beceiro, Tomas & Bou, 2013). In most cases, increased resistance is associated with decreased virulence and fitness (Geisinger & Isberg, 2017); however, no obvious correlation between antimicrobial resistance and virulence was observed in our *P. aeruginosa* isolates.

As a free-living organism, *P. aeruginosa* possesses numerous virulence factors and regulatory mechanisms for uptake of nutrients to colonise environmental niches and under suitable conditions become opportunistic pathogens. A recent genome analysis of a clinical strain revealed the presence of T3SS exoenzymes, elastase B, exotoxin A and *P. aeruginosa* Genomic Islands (PAGI) that collectively can induce pathogenicity (Murugan et al., 2017). However, virulence in *P. aeruginosa* is both multifactorial and combinatorial where multiple virulence factors cause overall pathogenicity, but the severity may differ in different strains (Lee et al., 2006). More than 60% of our isolates carried the following virulence factor genes, i.e., *apr*, *lasB*, *phzI*, *phzII*, *phzH*, *phzM*, *phzS*, *exoS*, *exoY*, *lecA* and *lecB*. Elastase LasB, a type II secretion system (T2SS)-dependent exoprotein (Braun et al., 1998) contributes to respiratory infections by degrading elastin (a major component of lung tissues) (Hamdaoui, Wund-Bisseret & Bieth, 1987). LasB can also evade host immune response by degrading complement components (Schultz & Miller, 1974), surfactant proteins A and D (Kuang et al., 2011; Mariencheck et al., 2003), airway lysozymes (Jacquot, Tournier & Puchelle, 1985), cytokines (Parmely et al., 1990) and immunoglobulins IgG and IgA (Bainbridge & Fick Jr, 1989; Heck et al., 1990). The role of LasB as a vital virulence factor has been proven in that after exposure to ciprofloxacin the surviving cells of *P. aeruginosa* in biofilms were able to secrete elastase B (Oldak & Trafny, 2005). There was high prevalence of soluble lectins, i.e., LecA and LecB in *P. aeruginosa* which bind to galactose and fucose, respectively (Avichezer & Gilboa-Garber, 1987; Gilboa-Garber, 1972) which are involved in host cell adhesion (Von Bismarck, Schneppenheim & Schumacher, 2001), cytotoxicity and permeability disorder affecting the alveolar capillary barrier leading to bacterial dissemination (Chemani et al., 2009). Our findings agree with previous reports (Bradbury et al., 2010; Finnan et al., 2004; Wu et al., 2003), indicating that they are highly conserved in the genome of *P. aeruginosa*.

Four effectors ExoS, ExoT, ExoU and ExoY are present in the T3SS system and the secretion of ExoS and ExoT in combination reduce anti-internalization by phagocytic cells (Shaver & Hauser, 2004). The absence of the *exoT* gene in our clinical isolates is similar to a report (Finnan et al., 2004) and it is possible that clinical isolates may delete a less virulent *exoT* gene to prevent the antagonizing effect of multiple effectors.

A small number (30 clinical and nine environmental) of our *P. aeruginosa* harboured the *exoU* gene, probably acquired via horizontal transfer (Berthelot et al., 2003) to become highly virulent and cytotoxic (Schulert et al., 2003; Wong-Beringer et al., 2008). This acquisition of *exoU* probably occurs only under selective pressure resulting in low prevalence in nature. The ExoU-positive environmental isolates were mostly from recreational parks situated in densely populated areas.

The co-existence of *exoS* and *exoU* is probably mutually exclusive in *P. aeruginosa* due to their distinct loci in the genome (Bradbury et al., 2010). Only eight of the total 219 isolates contained both genes probably providing a selective advantage for the survival of *P. aeruginosa* in a specific niche. Over time, a change in the genotype may take place by the deletion of one or the other gene to prevent antagonism. Therefore, the universal genotype of *P. aeruginosa* is either *exoU* or *exoS*.

Expression of T3SS by *P. aeruginosa* is associated with increasing virulence, but T3SS-negative isolates have been recovered from patients, which may have been contaminants or probably had remained dormant to evade host immune system for long-term survival (Jain et al., 2004). The expression of virulence genes involves multiple regulatory and metabolic networks (Winstanley, O’Brien & Brockhurst, 2016). A full set of T3SS effectors was only detected in our environmental isolates probably providing selective advantage to *P. aeruginosa* under harsh natural environments.

Phenazine-modifying enzymes phzM, phzS and phzH in *P. aeruginosa* (Recinos et al., 2012) are toxic and pH-dependent (Cezairliyan et al., 2013), and we observed that many harboured all the 3 *phzH*, *phzM* and *phzS* genes. It is likely that positive isolates produce more than one type of phenazine toxin that act over a wide pH range to ensure bacterial survival and colonization under different environmental conditions (Bradbury et al., 2010; Finnan et al., 2004).

Uptake of iron is crucial for colonization and *P. aeruginosa* is able to acquire Fe^3+^ from the host by producing iron chelating siderophore pyoverdine; the responsible gene (*pvdA*) was present in 48% to 70% of our clinical isolates, but may lose this ability during long periods of colonization (De Vos et al., 2001).

## Conclusion

*P. aeruginosa* is ubiquitous and an opportunistic pathogen causing infections especially in immunocompromised patients. It is equipped with natural drug resistance and virulence mechanisms for survival in harsh environments. However, it can become resistant under selective pressure leading to increase in pseudomonal infections and possibly therapeutic failures.

Our findings indicate a rise in resistance to antipseudomonal drugs in two hospitals in Malaysia over the past 30 years. Therefore, it is necessary to implement a programme of periodic surveillance and standardization of a protocol for antipseudomonal therapy by the relevant authorities. In addition, the observation of antimicrobial resistance in environmental isolates from densely populated areas highlights the importance of increased public health awareness.

The limitation of this study was the small number of isolates, but our findings provide basic knowledge of epidemiology, antimicrobial resistance and virulence traits of *P. aeruginosa*. Works involved other typing methods such as multi-locus sequencing typing (MLST) or multi-virulent sequencing typing (MLVA) (Teh, Chua & Thong, 2011) could also be carried out to gather more differential information between clinical and environmental isolates of *Pseudomonas aeruginosa.* A robust surveillance of antimicrobial susceptibility should be implemented to monitor and prevent dissemination of pathogenic multidrug resistant strains in Malaysia.

##  Supplemental Information

10.7717/peerj.6217/supp-1 Supplemental Information 1 *P. aeruginosa* used in this study √ indicates presence of virulence genes; x indicates absence of virulence genes; S indicates susceptible; I indicates intermediate; R indicates resistant (according to CLSI guidelines M100-S26).

10.7717/peerj.6217/supp-2 Supplemental Information 2 ERIC-PCR fingerprints of clinical and environmental isolates of *P. aeruginosa*

10.7717/peerj.6217/supp-3 Supplemental Information 3 Detection of *P. aeruginosa* from isolation periods 1977 to 1985 and 2015 in various specimens Notes: NA source of specimen not available. Significant *P*-values are shown in bold.

10.7717/peerj.6217/supp-4 Supplemental Information 4 Virulence genes screening and antimicrobial susceptibility test results √ indicates presence of virulence genes; x indicates absence of virulence genes; S indicates susceptible; I indicates intermediate; R indicates resistant (according to CLSI guidelines M100-S26).

10.7717/peerj.6217/supp-5 Supplemental Information 5 Sequencing data

## Additional Information and Declarations

### Data Availability

The following information was supplied regarding data availability: The raw data are provided in the Supplemental Files.
