# Supplementary material for: Antimicrobial susceptibility and virulence genes of clinical and environmental isolates of Pseudomonas aeruginosa
Source: PeerJ. 2019 Jan 22;7:e6217. doi: 10.7717/peerj.6217 (PMC6346980; doi:10.7717/peerj.6217)
Supplement: Supplemental Information 1 — √ indicates presence of virulence genes; x indicates absence of virulence genes; S indicates susceptible; I indicates intermediate; R indicates resistant (according to CLSI guidelines M100-S26). [file peerj-07-6217-s001.docx]

***P. aeruginosa* environmental strains from fresh water (*n* = 114)**

|  |  |  |  |  | **virulence genes** | | | | | | | | | | | | | | | **Antimicrobial susceptibility** | | | | | | | | | | |
| --- | --- | --- | --- | --- | --- | --- | --- | --- | --- | --- | --- | --- | --- | --- | --- | --- | --- | --- | --- | --- | --- | --- | --- | --- | --- | --- | --- | --- | --- | --- |
| **No.** | **Strain** | **Date** | **Location** | **GPS** | ***apr*** | ***lasB*** | ***phzI*** | ***phzII*** | ***phzH*** | ***phzM*** | ***phzS*** | ***exoS*** | ***exoT*** | ***exoU*** | ***exoY*** | ***pvdA*** | ***pilB*** | ***lecA*** | ***lecB*** | **MEM** | **DOR** | **IMI** | **CAZ** | **PTZ** | **TIM** | **PRL** | **CIP** | **AK** | **CN** | **NET** |
| 1 | P2 | 24/8/2015 | fish pond, UMMC lobby (Kuala Lumpur) | N03°06.799 E101°39.136 | X | √ | √ | X | √ | √ | √ | X | √ | √ | √ | X | X | √ | X | S | S | S | S | S | I | S | S | S | S | S |
| 2 | P5 |  |  |  | X | X | X | X | X | X | X | X | X | X | X | X | X | X | X | **R** | **R** | S | S | S | I | S | S | S | S | S |
| 3 | F1 | 24/8/2015 | fountain, Gynae & Peds Building UMMC (Kuala Lumpur) | N03°06.726 E101°39.260 | X | X | X | X | X | X | X | X | X | X | X | X | X | X | X | I | **R** | S | S | S | I | S | S | S | S | S |
| 4 | F5 |  |  |  | X | X | X | X | X | X | X | X | X | X | X | X | X | X | X | I | **R** | S | S | S | I | S | S | S | S | S |
| 5 | F8 |  |  |  | X | X | X | X | X | X | X | X | X | X | X | X | X | X | X | I | **R** | S | S | S | I | S | S | S | S | S |
| 6 | F9a |  |  |  | X | X | X | X | X | X | X | X | X | X | X | X | X | X | X | I | **R** | S | S | S | I | S | S | S | S | S |
| 7 | UL2 | 09/01/2015 | Varsity Lake, University Malaya (Kuala Lumpur) | N03°07.165 E101°39.514 | √ | √ | √ | X | √ | √ | √ | √ | X | X | √ | X | X | √ | √ | S | S | S | S | S | I | S | S | S | S | S |
| 8 | UL4a |  |  |  | X | X | X | X | X | X | X | X | X | X | X | X | X | X | X | **R** | **R** | S | S | S | I | S | S | S | S | S |
| 9 | UL6 |  |  |  | √ | √ | √ | X | √ | √ | √ | √ | √ | X | √ | X | X | √ | √ | S | S | S | S | S | S | **R** | S | S | S | S |
| 10 | UL17 |  |  |  | X | X | X | X | X | X | X | X | X | X | X | X | X | X | X | **R** | **R** | **R** | S | S | I | S | S | S | S | S |
| 11 | UL19 |  |  |  | √ | √ | √ | √ | √ | √ | √ | √ | √ | X | √ | √ | X | √ | √ | S | S | S | S | S | I | S | S | S | S | S |
| 12 | UW2 | 09/01/2015 | pool, Dewan Tunku Canselor, University Malaya (Kuala Lumpur) | N03°07.311 E101°39.452 | X | X | X | X | X | X | X | X | X | X | X | X | X | X | X | **R** | **R** | **R** | S | **R** | I | **R** | S | S | S | S |
| 13 | UW4 |  |  |  | √ | √ | √ | √ | √ | √ | √ | √ | √ | X | √ | √ | X | √ | √ | S | S | S | S | S | I | S | S | S | S | S |
| 14 | UW5 |  |  |  | √ | √ | √ | √ | √ | √ | √ | √ | √ | X | √ | √ | X | √ | √ | S | S | S | S | S | I | S | S | S | S | S |
| 15 | UW7b |  |  |  | X | X | X | X | X | X | X | X | X | X | X | X | X | X | X | **R** | **R** | S | S | S | S | I | S | S | S | S |
| 16 | UW10 |  |  |  | √ | √ | √ | √ | √ | √ | √ | √ | √ | X | √ | √ | X | √ | √ | S | S | S | S | S | S | S | S | S | S | S |
| 17 | UW12 |  |  |  | √ | √ | √ | √ | √ | √ | √ | √ | √ | X | √ | √ | X | √ | √ | S | S | S | S | S | I | S | S | S | S | S |
| 18 | UW13 |  |  |  | √ | √ | √ | √ | √ | X | √ | √ | √ | √ | √ | √ | X | √ | √ | S | S | S | S | S | I | S | S | S | S | S |
| 19 | UW14 |  |  |  | √ | √ | √ | X | √ | √ | √ | √ | √ | X | √ | √ | X | √ | X | S | S | S | S | S | I | S | S | S | S | S |
| 20 | UW16 |  |  |  | √ | √ | √ | X | √ | √ | √ | X | √ | √ | √ | X | X | √ | X | S | S | S | S | S | I | S | S | S | S | S |
| 21 | UW18 |  |  |  | X | X | X | X | X | X | X | X | X | X | X | X | X | X | X | **R** | **R** | **R** | S | **R** | I | **R** | S | S | S | S |
| 22 | UW19 |  |  |  | √ | √ | √ | √ | √ | √ | √ | √ | √ | X | √ | √ | X | √ | √ | S | S | S | S | S | I | S | S | S | S | S |
| 23 | UW21a |  |  |  | X | X | X | X | X | X | X | X | X | X | X | X | X | X | X | I | I | S | S | S | I | S | S | S | S | S |
| 24 | UW23 |  |  |  | √ | √ | √ | √ | √ | √ | √ | √ | √ | X | √ | √ | X | √ | √ | S | S | S | S | S | I | S | S | S | S | S |
| 25 | DL5 | 09/06/2015 | lake, Desa Parkcity recreational park (Kuala Lumpur) | N03°11.198 E101°37.714 | X | X | X | X | X | X | X | X | X | X | X | X | X | X | X | I | I | S | S | S | I | **R** | **R** | S | S | S |
| 26 | DL6 |  |  |  | √ | √ | √ | X | √ | √ | √ | √ | X | X | X | √ | X | √ | √ | S | S | S | S | S | I | S | S | S | S | S |
| 27 | DL12 |  |  |  | √ | √ | √ | √ | √ | √ | √ | √ | X | X | √ | √ | X | √ | √ | S | S | S | S | S | I | I | S | S | S | S |
| 28 | DL13 |  |  |  | √ | √ | √ | √ | √ | √ | √ | √ | X | X | √ | X | X | √ | √ | S | S | S | S | S | S | S | S | S | S | S |
| 29 | DL26 |  |  |  | √ | √ | √ | √ | √ | X | √ | √ | X | X | √ | √ | X | √ | √ | S | S | S | S | S | I | S | S | S | S | S |
| 30 | DL27b |  |  |  | X | X | X | X | X | X | X | X | X | X | X | X | X | X | X | **R** | **R** | S | S | S | S | S | S | S | S | S |
| 31 | ML1 | 09/06/2015 | lake, Menjalara recreational park (Kuala Lumpur) | N03°11.736 E101°37.680 | √ | X | √ | X | X | √ | X | √ | X | X | X | X | X | X | X | S | S | S | S | S | I | S | S | S | S | S |
| 32 | ML2 |  |  |  | √ | √ | √ | X | √ | √ | √ | √ | X | X | √ | X | X | √ | √ | S | S | S | S | S | I | S | S | S | S | S |
| 33 | ML3 |  |  |  | √ | √ | √ | √ | √ | √ | √ | √ | X | X | √ | √ | X | √ | √ | S | S | S | S | S | I | S | S | S | S | S |
| 34 | ML11 |  |  |  | √ | √ | √ | X | √ | √ | √ | √ | X | X | √ | X | X | √ | X | S | S | S | S | S | I | S | S | S | S | S |
| 35 | ML12 |  |  |  | √ | √ | √ | √ | √ | √ | √ | √ | X | X | √ | X | X | √ | √ | S | S | S | S | S | I | S | S | S | S | S |
| 36 | ML14 |  |  |  | √ | √ | √ | √ | √ | √ | √ | √ | X | X | √ | X | X | √ | √ | S | S | S | S | S | I | S | S | S | S | S |
| 37 | ML15 |  |  |  | √ | √ | √ | √ | √ | X | √ | √ | X | X | √ | X | X | √ | X | S | S | S | S | S | I | S | S | S | S | S |
| 38 | ML16 |  |  |  | √ | √ | √ | √ | √ | X | √ | √ | X | X | √ | X | X | √ | √ | S | S | S | S | S | I | S | S | S | S | S |
| 39 | ML18 |  |  |  | √ | √ | √ | √ | √ | √ | √ | √ | X | X | √ | √ | X | √ | √ | S | S | S | S | S | I | S | S | S | S | S |
| 40 | ML19 |  |  |  | √ | √ | √ | X | √ | √ | √ | √ | X | X | √ | √ | X | √ | √ | S | S | S | S | S | I | S | S | S | S | S |
| 41 | ML21 |  |  |  | √ | √ | √ | √ | √ | X | √ | √ | X | X | √ | X | X | √ | √ | S | S | S | S | S | I | S | S | S | S | S |
| 42 | ML24 |  |  |  | √ | √ | √ | √ | √ | X | √ | √ | √ | X | √ | X | X | √ | √ | S | S | S | S | S | I | S | S | S | S | S |
| 43 | ML25 |  |  |  | √ | √ | √ | √ | √ | √ | √ | √ | X | X | √ | √ | X | √ | √ | S | S | S | S | S | I | S | S | S | S | S |
| 44 | ML26 |  |  |  | √ | √ | √ | X | √ | √ | √ | √ | X | X | √ | √ | X | √ | √ | S | S | S | S | S | I | S | S | S | S | S |
| 45 | ML27 |  |  |  | √ | √ | √ | √ | √ | √ | √ | √ | √ | X | √ | √ | X | √ | √ | S | S | S | S | S | I | S | S | S | S | S |
| 46 | JD1 | 13/9/2015 | drain, Juara Bahau (Negeri Sembilan) | N02°48.165 E102°24.462 | √ | √ | √ | X | √ | √ | √ | X | X | √ | √ | X | X | √ | X | S | S | S | S | S | I | S | S | S | S | S |
| 47 | JD2 |  |  |  | X | X | X | X | X | X | X | X | X | X | X | X | X | X | X | S | S | S | S | S | S | S | S | S | S | S |
| 48 | JD11 |  |  |  | √ | √ | √ | √ | √ | √ | √ | √ | X | X | √ | X | X | √ | √ | S | S | S | S | S | I | S | S | S | S | S |
| 49 | JD13 |  |  |  | √ | √ | √ | X | √ | √ | √ | X | X | √ | √ | X | X | √ | X | S | S | S | S | S | I | S | S | S | S | S |
| 50 | JD14 |  |  |  | X | √ | √ | X | √ | √ | X | √ | X | X | √ | X | X | X | √ | S | S | S | S | S | I | S | S | S | S | S |
| 51 | JD23 |  |  |  | √ | √ | √ | √ | √ | √ | √ | √ | X | X | √ | X | X | √ | √ | S | S | S | S | S | I | S | S | S | S | S |
| 52 | JD26 |  |  |  | √ | √ | √ | √ | √ | √ | √ | √ | X | X | √ | √ | X | √ | √ | S | S | S | S | S | I | S | S | S | S | S |
| 53 | JD28 |  |  |  | X | X | X | X | X | X | X | X | X | X | X | X | X | X | X | S | S | S | S | S | I | S | S | S | S | S |
| 54 | BL2 | 13/9/2015 | lake, Bahau recreational park (Negeri Sembilan) | N02°48.456 E102°23.910 | √ | √ | √ | √ | √ | √ | √ | √ | X | X | √ | X | X | √ | X | S | S | S | S | S | I | S | S | S | S | S |
| 55 | BL4 |  |  |  | √ | √ | √ | √ | √ | √ | √ | √ | X | X | √ | X | X | √ | X | S | S | S | S | S | I | S | S | S | S | S |
| 56 | BL6 |  |  |  | √ | √ | √ | √ | √ | √ | √ | √ | √ | X | √ | X | X | √ | X | S | S | S | S | S | I | S | S | S | S | S |
| 57 | BL7 |  |  |  | √ | √ | √ | √ | √ | √ | √ | √ | √ | X | √ | X | X | √ | X | S | S | S | S | S | I | I | S | S | S | S |
| 58 | BL11 |  |  |  | √ | √ | √ | √ | √ | √ | √ | √ | √ | X | √ | X | X | √ | X | S | S | S | S | S | I | S | S | S | S | S |
| 59 | BL18 |  |  |  | √ | √ | √ | √ | √ | √ | √ | √ | √ | X | √ | X | X | √ | X | S | S | S | S | S | I | S | S | S | S | S |
| 60 | BL20 |  |  |  | √ | √ | √ | √ | √ | √ | √ | √ | √ | X | √ | √ | X | √ | √ | S | S | S | S | S | I | S | S | S | S | S |
| 61 | UB3a | 13/9/2015 | Water fall, Ulu Bendul recreational park (Negeri Sembilan) | N02°43.782 E102°04.664 | √ | √ | √ | √ | √ | √ | √ | √ | √ | X | √ | √ | X | √ | √ | S | S | S | S | S | I | S | S | S | S | S |
| 62 | UB7 |  |  |  | √ | √ | √ | √ | √ | √ | √ | √ | √ | √ | √ | √ | X | √ | √ | S | S | S | S | S | I | S | S | S | S | S |
| 63 | UB8 |  |  |  | √ | √ | √ | √ | √ | √ | √ | √ | √ | X | √ | X | X | √ | √ | S | S | S | S | S | I | S | S | S | S | S |
| 64 | UB9 |  |  |  | √ | √ | √ | √ | √ | √ | √ | √ | √ | X | √ | √ | X | √ | √ | S | S | S | S | S | I | S | S | S | S | S |
| 65 | UB11 |  |  |  | √ | √ | √ | √ | √ | √ | √ | √ | X | √ | √ | √ | X | √ | √ | S | S | S | S | S | I | S | S | S | S | S |
| 66 | UB12 |  |  |  | √ | √ | √ | √ | √ | √ | √ | √ | √ | X | √ | √ | X | √ | √ | S | S | S | S | S | I | S | S | S | S | S |
| 67 | UB13a |  |  |  | √ | √ | √ | √ | √ | √ | √ | √ | √ | X | √ | √ | X | √ | √ | S | S | S | S | S | I | S | S | S | S | S |
| 68 | UB14a |  |  |  | √ | √ | √ | √ | √ | √ | √ | √ | √ | X | √ | √ | X | √ | √ | S | S | S | S | S | I | S | S | S | S | S |
| 69 | UB15 |  |  |  | √ | √ | √ | √ | √ | √ | √ | √ | √ | X | √ | √ | X | √ | √ | S | S | S | S | S | I | S | S | S | S | S |
| 70 | UB17 |  |  |  | √ | √ | √ | √ | √ | √ | √ | √ | X | X | √ | √ | X | √ | √ | S | S | S | S | S | I | S | S | S | S | S |
| 71 | UB21 |  |  |  | √ | √ | √ | √ | √ | √ | √ | √ | √ | X | √ | √ | X | √ | √ | S | S | S | S | S | I | S | S | S | S | S |
| 72 | UB24 |  |  |  | √ | √ | √ | √ | √ | √ | √ | √ | √ | X | √ | √ | X | √ | √ | S | S | S | S | S | I | S | S | S | S | S |
| 73 | UB26 |  |  |  | √ | √ | √ | √ | √ | √ | √ | √ | √ | X | √ | √ | X | √ | √ | S | S | S | S | S | I | I | **R** | S | S | S |
| 74 | UB30 |  |  |  | √ | √ | √ | √ | √ | √ | √ | √ | √ | X | √ | X | X | √ | √ | S | S | S | S | S | I | S | S | S | S | S |
| 75 | CW3b | 18/10/2015 | fish pond, Chi Wen secondary school (Negeri Sembilan) | N02°48.554 E102°24.212 | X | X | X | X | X | X | X | X | X | X | X | X | X | X | X | **R** | I | S | S | S | I | I | S | S | S | S |
| 76 | CW6b |  |  |  | X | X | X | X | X | X | X | X | X | X | X | X | X | X | X | **R** | **R** | S | S | I | I | I | S | S | S | S |
| 77 | CW19 |  |  |  | X | X | X | X | X | X | X | X | X | X | X | X | X | X | X | **R** | **R** | S | S | I | I | I | S | S | S | S |
| 78 | TW9 | 18/10/2015 | well, chinese temple (Negeri Sembilan) | N02°48.087 E102°24.264 | √ | √ | √ | √ | √ | X | √ | √ | X | X | √ | √ | X | √ | √ | S | S | S | S | S | I | S | S | S | S | S |
| 79 | TW19 |  |  |  | √ | √ | √ | √ | √ | √ | √ | √ | X | X | √ | √ | X | √ | √ | S | S | S | S | S | I | S | S | S | S | S |
| 80 | TW20 |  |  |  | √ | √ | √ | √ | √ | √ | √ | √ | X | X | √ | √ | X | √ | √ | S | S | S | S | S | I | S | S | S | S | S |
| 81 | TW23 |  |  |  | √ | √ | √ | √ | √ | √ | √ | √ | X | X | √ | √ | X | √ | √ | S | S | S | S | S | I | S | S | S | S | S |
| 82 | PD1 | 18/10/2015 | paddy field (Negeri Sembilan) | N02°49.667 E102°20.056 | √ | √ | √ | √ | √ | √ | √ | √ | X | X | √ | X | X | √ | √ | S | S | S | S | S | I | S | S | S | S | S |
| 83 | PD7 |  |  |  | √ | √ | √ | √ | √ | √ | √ | √ | X | X | √ | X | X | √ | √ | S | S | S | S | S | I | I | S | S | S | S |
| 84 | PD18 |  |  |  | √ | √ | √ | √ | √ | √ | √ | √ | X | X | √ | X | X | √ | √ | S | S | S | S | S | I | S | S | S | S | S |
| 85 | LP1 | 11/01/2015 | pool, Layang-layang recreational park (Kuala Lumpur) | N03°13.634 E101°38.935 | √ | √ | √ | √ | √ | √ | √ | √ | √ | X | √ | √ | √ | √ | √ | S | S | S | S | S | I | S | S | S | S | S |
| 86 | LP4 |  |  |  | √ | √ | √ | √ | √ | √ | √ | √ | √ | X | √ | X | X | √ | √ | S | S | S | S | S | S | S | S | S | S | S |
| 87 | LP6 |  |  |  | √ | √ | √ | √ | √ | √ | √ | √ | √ | X | √ | X | X | √ | √ | S | S | S | S | S | I | S | S | S | S | S |
| 88 | LP7 |  |  |  | √ | √ | √ | √ | √ | √ | √ | √ | X | X | √ | √ | X | √ | √ | S | S | S | S | S | I | S | S | S | S | S |
| 89 | LP10 |  |  |  | √ | √ | √ | √ | √ | X | √ | √ | X | X | √ | √ | X | √ | √ | S | S | S | S | S | I | S | S | S | S | S |
| 90 | LP15 |  |  |  | √ | √ | √ | √ | √ | √ | √ | √ | X | X | √ | √ | X | √ | √ | S | S | S | S | S | I | S | S | S | S | S |
| 91 | LP16 |  |  |  | √ | √ | √ | √ | √ | √ | √ | √ | √ | X | √ | √ | X | √ | √ | S | S | S | S | S | I | S | S | S | S | S |
| 92 | LP18 |  |  |  | √ | √ | √ | √ | √ | √ | √ | √ | √ | X | √ | √ | X | √ | √ | S | S | S | S | S | I | S | S | S | S | S |
| 93 | LP21 |  |  |  | √ | √ | √ | √ | √ | √ | √ | √ | √ | X | √ | √ | X | √ | √ | S | S | S | S | S | I | S | S | S | S | S |
| 94 | LP25 |  |  |  | √ | √ | √ | X | √ | √ | √ | X | √ | √ | √ | √ | X | √ | X | S | S | S | S | S | I | I | S | S | S | S |
| 95 | LP29 |  |  |  | √ | √ | √ | √ | √ | √ | √ | √ | √ | X | √ | X | X | √ | √ | S | S | S | S | S | I | **R** | S | S | S | S |
| 96 | LP32 |  |  |  | √ | √ | √ | √ | √ | √ | √ | √ | √ | X | √ | X | X | √ | √ | S | S | S | S | S | I | S | S | S | S | S |
| 97 | TL1 | 11/01/2015 | lake, TTDI recreational park (Petaling Jaya) | N03°08.834 E101°37.945 | √ | √ | √ | √ | √ | √ | √ | √ | √ | X | √ | √ | X | √ | √ | S | S | S | S | S | I | S | S | S | S | S |
| 98 | TL3 |  |  |  | √ | √ | √ | √ | √ | √ | √ | √ | √ | X | √ | X | X | √ | √ | S | S | S | S | S | I | S | S | S | S | S |
| 99 | TL4 |  |  |  | √ | √ | √ | √ | √ | √ | √ | √ | √ | X | √ | X | X | √ | √ | S | S | S | S | S | I | S | S | S | S | S |
| 100 | TL6 |  |  |  | √ | √ | √ | √ | √ | √ | √ | √ | √ | X | √ | X | X | √ | √ | S | S | S | S | S | I | S | S | S | S | S |
| 101 | TL8 |  |  |  | √ | √ | √ | √ | √ | √ | √ | √ | √ | X | √ | √ | X | √ | √ | S | S | S | S | S | I | S | S | S | S | S |
| 102 | TL9 |  |  |  | √ | √ | √ | √ | √ | √ | √ | √ | √ | X | √ | √ | X | √ | √ | S | S | S | S | S | I | S | S | S | S | S |
| 103 | TL10 |  |  |  | √ | √ | √ | √ | √ | X | √ | √ | √ | X | √ | √ | X | √ | √ | S | S | S | S | S | I | S | S | S | S | S |
| 104 | TL11 |  |  |  | √ | √ | √ | √ | √ | X | √ | √ | √ | X | √ | X | X | √ | √ | S | S | S | S | S | I | S | S | S | S | S |
| 105 | TL12 |  |  |  | √ | √ | √ | √ | √ | √ | √ | √ | √ | X | √ | √ | X | √ | √ | S | S | S | S | S | I | S | S | S | S | S |
| 106 | TL14 |  |  |  | √ | √ | √ | X | X | √ | X | √ | √ | X | X | X | √ | √ | √ | S | S | S | S | S | I | S | S | S | S | S |
| 107 | TL21 |  |  |  | √ | √ | √ | √ | √ | √ | √ | √ | √ | X | √ | X | X | √ | √ | S | S | S | S | S | I | S | S | S | S | S |
| 108 | TL22 |  |  |  | √ | √ | √ | √ | √ | √ | √ | √ | √ | X | √ | √ | X | √ | √ | S | S | S | S | S | I | S | S | S | S | S |
| 109 | TL23 |  |  |  | √ | √ | √ | √ | √ | √ | √ | √ | √ | X | √ | X | X | √ | √ | S | S | S | S | S | I | S | S | S | S | S |
| 110 | TL24 |  |  |  | √ | √ | √ | √ | √ | √ | √ | √ | √ | X | √ | X | X | √ | √ | S | S | S | S | S | I | S | S | S | S | S |
| 111 | TL27 |  |  |  | √ | √ | √ | √ | √ | √ | √ | √ | √ | X | √ | √ | X | √ | √ | S | S | S | S | S | I | S | S | S | S | S |
| 112 | LL1 | 11/01/2015 | lake, Layang-layang recreational park (Kuala Lumpur) | N03°13.634 E101°38.935 | √ | √ | √ | √ | √ | X | √ | √ | √ | X | √ | √ | X | √ | √ | S | S | S | S | S | I | S | S | S | S | S |
| 113 | LL3 |  |  |  | √ | √ | √ | X | √ | √ | √ | √ | √ | √ | √ | √ | X | √ | √ | S | S | S | S | S | I | S | S | S | S | S |
| 114 | LL10 |  |  |  | √ | √ | √ | √ | √ | √ | √ | √ | √ | X | √ | X | X | √ | √ | S | S | S | S | S | I | S | S | S | S | S |

√ indicates presence of virulence genes; x indicates absence of virulence genes; S indicates susceptible; I indicates intermediate; R indicates resistant (according to CLSI guidelines M100-S26).

***P. aeruginosa* clinical strains from Johor Bahru, Malaysia (*n* = 53)**

|  |  |  | **Virulence genes** | | | | | | | | | | | | | | | **Antimicrobial susceptibility** | | | | | | | | | | |
| --- | --- | --- | --- | --- | --- | --- | --- | --- | --- | --- | --- | --- | --- | --- | --- | --- | --- | --- | --- | --- | --- | --- | --- | --- | --- | --- | --- | --- |
| **Strain** | **Year received** | **Source of specimen** | ***apr*** | ***lasB*** | ***phzI*** | ***phzII*** | ***phzH*** | ***phzM*** | ***phzS*** | ***exoS*** | ***exoT*** | ***exoU*** | ***exoY*** | ***pvdA*** | ***pilB*** | ***lecA*** | ***lecB*** | **MEM** | **DOR** | **IMI** | **CAZ** | **PTZ** | **TIM** | **PRL** | **CIP** | **AK** | **CN** | **NET** |
| J1 | 2/3/2015 | Urine | √ | √ | √ | √ | √ | √ | √ | √ | x | x | √ | √ | x | √ | √ | S | S | S | S | S | I | S | S | S | S | S |
| J2 | 2/8/2015 | Urine | √ | √ | √ | √ | √ | √ | √ | √ | x | x | √ | √ | x | √ | √ | S | S | S | S | S | I | S | S | S | S | S |
| J3 | 26/1/2015 | Tissue (leg) | x | x | x | x | x | x | x | x | x | x | x | x | x | x | x | **R** | **R** | **R** | **R** | **R** | **R** | **R** | S | S | S | S |
| J4 | 2/9/2015 | Bronchial aspirate | √ | √ | √ | √ | √ | √ | √ | √ | x | x | √ | √ | x | √ | √ | S | S | S | S | S | S | S | S | S | S | S |
| J5 | 4/12/2015 | Blood | √ | √ | √ | √ | √ | √ | √ | √ | x | x | √ | √ | x | √ | √ | S | S | S | S | S | S | S | S | S | S | S |
| J6 | 2/9/2015 | Sputum | √ | √ | √ | √ | √ | √ | √ | √ | x | x | √ | √ | x | √ | √ | S | S | S | S | S | I | S | S | S | S | S |
| J7 | 2/9/2015 | Bronchial aspirate | √ | √ | √ | √ | √ | √ | √ | √ | x | x | √ | √ | x | √ | √ | S | S | S | S | S | I | S | S | S | S | S |
| J8 | 4/10/2015 | Urine | √ | √ | √ | √ | √ | √ | √ | √ | x | x | √ | √ | x | √ | √ | S | S | S | S | S | I | I | S | S | S | S |
| J9 | 2/10/2015 | Tissue | √ | √ | √ | √ | √ | √ | √ | x | x | √ | √ | √ | x | √ | x | S | S | S | S | S | I | S | S | S | S | S |
| J10 | 2/9/2015 | Slough | √ | √ | √ | √ | √ | √ | √ | x | x | √ | √ | √ | √ | √ | x | S | S | S | S | S | I | S | S | S | S | S |
| J11 | 2/9/2015 | Tissue | x | √ | √ | √ | √ | √ | √ | x | x | √ | √ | √ | x | √ | x | **R** | **R** | **R** | **R** | **R** | **R** | **R** | **R** | **R** | **R** | **R** |
| J12 | 13/2/2015 | Tissue | √ | √ | √ | √ | √ | √ | √ | x | x | √ | √ | √ | x | √ | x | S | S | S | S | S | I | S | S | S | S | S |
| J13 | 4/3/2015 | Urine | √ | √ | √ | √ | √ | √ | √ | √ | x | x | √ | √ | x | √ | √ | S | S | S | S | S | I | S | S | S | S | S |
| J14 | 14/4/2015 | Urine | √ | √ | √ | √ | √ | √ | √ | √ | x | x | √ | √ | x | √ | √ | S | S | S | S | S | I | S | S | S | S | S |
| J15 | 2/8/2015 | Urine | √ | √ | √ | √ | √ | √ | √ | √ | x | x | √ | √ | x | √ | √ | S | S | S | S | S | I | S | S | S | S | S |
| J16 | 4/11/2015 | Blood | √ | √ | √ | √ | √ | √ | √ | √ | x | x | √ | √ | x | √ | √ | S | S | S | S | S | I | S | S | S | S | S |
| J17 | 4/11/2015 | Bronchial aspirate | √ | √ | √ | √ | √ | x | √ | √ | x | x | √ | x | x | √ | √ | S | S | S | S | S | I | S | S | S | S | S |
| J18 | 4/10/2015 | Urine | √ | √ | √ | √ | √ | √ | √ | √ | x | x | √ | x | x | √ | √ | S | S | S | S | S | I | S | S | S | S | S |
| J19 | 27/1/2015 | Tissue | √ | √ | √ | √ | √ | x | √ | √ | x | x | √ | x | x | √ | √ | S | S | S | S | S | I | S | S | S | S | S |
| J20 | 14/4/2015 | Urine | √ | √ | √ | √ | √ | x | √ | x | x | √ | √ | √ | x | √ | x | **R** | **R** | **R** | **R** | **R** | **R** | **R** | **R** | **R** | **R** | **R** |
| J21 | 5/3/2015 | CSF | √ | √ | √ | √ | √ | x | √ | √ | x | x | √ | x | x | √ | √ | S | S | S | S | S | I | S | S | S | S | S |
| J22 | 26/4/2015 | Pus | √ | √ | √ | √ | √ | x | √ | √ | x | x | √ | √ | x | √ | x | S | S | S | S | S | I | S | S | S | S | S |
| J23 | 2/8/2015 | Urine | √ | √ | √ | √ | √ | x | √ | √ | x | x | √ | √ | x | √ | √ | S | S | S | S | S | I | S | S | S | S | S |
| J24 | 25/4/2015 | Urine | √ | √ | √ | √ | √ | x | √ | x | x | √ | √ | √ | √ | √ | x | S | S | S | S | S | I | S | S | S | S | S |
| J25 | 22/4/2015 | Blood | √ | √ | √ | √ | √ | x | √ | x | x | √ | √ | √ | x | √ | x | **R** | **R** | I | **R** | I | **R** | I | **R** | S | **R** | **R** |
| J26 | 20/4/2015 | Bronchial aspirate | √ | √ | √ | √ | √ | x | √ | √ | x | x | √ | √ | x | √ | √ | S | S | S | S | I | **R** | **R** | S | S | S | S |
| J27 | 4/10/2015 | Urine | √ | √ | √ | √ | √ | x | √ | √ | x | x | √ | √ | x | √ | √ | S | S | S | S | S | S | S | S | S | S | S |
| J28 | 20/4/2015 | Bronchial aspirate | √ | √ | √ | √ | √ | √ | √ | √ | x | x | √ | x | x | √ | √ | S | S | S | S | I | **R** | I | S | S | S | S |
| J29 | 25/4/2015 | Urine | √ | √ | √ | √ | √ | √ | √ | √ | x | x | √ | x | x | √ | √ | S | S | S | S | S | I | S | S | S | S | S |
| J30 | 29/5/2015 | Tissue | √ | √ | √ | √ | √ | √ | √ | √ | x | x | √ | √ | x | √ | √ | S | S | S | S | S | I | S | S | S | S | S |
| J31 | 23/5/2015 | Tissue | √ | √ | √ | √ | √ | √ | √ | x | x | √ | √ | √ | x | √ | x | S | S | S | S | S | **R** | S | S | S | S | S |
| J32 | 24/5/2015 | Bronchial aspirate | √ | √ | √ | √ | √ | √ | √ | √ | x | x | √ | √ | √ | √ | √ | S | S | S | S | S | I | S | S | S | S | S |
| J33 | 24/5/2015 | Pus | √ | √ | √ | √ | √ | √ | √ | x | x | √ | √ | √ | x | √ | x | S | S | S | S | I | **R** | **R** | S | S | S | S |
| J34 | 20/4/2015 | Bronchial aspirate | √ | √ | √ | √ | √ | √ | √ | √ | x | x | √ | x | x | √ | √ | S | S | S | S | S | I | S | S | S | S | S |
| J35 | 25/5/2015 | Bronchial aspirate | √ | √ | √ | √ | √ | √ | √ | √ | x | x | √ | √ | x | √ | √ | S | S | S | S | S | I | S | S | S | S | S |
| J36 | 14/2/2015 | Tissue | √ | √ | √ | √ | √ | √ | √ | √ | x | x | √ | √ | x | √ | √ | S | S | S | S | S | I | S | S | **R** | **R** | **R** |
| J37 | 3/8/2015 | Bronchial aspirate | x | √ | √ | √ | √ | √ | √ | √ | x | x | √ | √ | x | √ | √ | S | S | S | S | S | I | S | S | S | S | S |
| J38 | 3/3/2015 | Tissue | √ | √ | √ | √ | √ | x | √ | √ | x | x | √ | √ | x | √ | √ | S | S | S | **R** | **R** | **R** | **R** | S | S | S | S |
| J39 | 3/3/2015 | Tissue | √ | √ | √ | √ | √ | √ | √ | √ | x | x | √ | √ | x | √ | √ | S | S | S | S | S | I | S | S | S | S | S |
| J40 | 30/3/2015 | Tissue | √ | √ | √ | x | √ | √ | √ | x | x | √ | √ | x | x | √ | x | S | S | S | S | S | I | S | S | S | S | S |
| J41 | 25/5/2015 | Bronchial aspirate | √ | √ | √ | √ | √ | √ | √ | √ | x | x | √ | √ | x | √ | √ | S | S | S | S | S | I | S | S | S | S | S |
| J42 | 28/5/2015 | Urine | √ | √ | √ | √ | √ | √ | √ | √ | x | x | √ | x | √ | √ | √ | S | S | S | S | S | I | S | S | S | S | S |
| J43 | 14/2/2015 | Tissue | √ | √ | √ | √ | √ | x | √ | √ | x | √ | √ | x | x | √ | √ | S | S | S | S | S | I | S | S | S | S | S |
| J44 | 15/2/2015 | Urine | √ | √ | √ | √ | √ | x | √ | √ | x | x | √ | √ | x | √ | √ | S | S | S | S | S | I | S | S | S | S | S |
| J45 | 29/5/2015 | Urine | x | x | x | x | x | x | x | x | x | x | x | x | x | x | x | S | S | S | S | S | **R** | S | S | S | S | S |
| J46 | 21/2/2015 | Bronchial aspirate | √ | √ | √ | √ | √ | x | √ | √ | x | x | √ | x | x | √ | √ | S | S | S | S | S | I | S | S | S | S | S |
| J47 | 3/6/2015 | Bronchial aspirate | √ | √ | √ | √ | √ | x | √ | √ | x | x | √ | √ | x | √ | √ | S | I | **R** | S | S | I | S | S | S | S | S |
| J48 | 26/5/2015 | Tissue (toe) | √ | √ | √ | √ | √ | x | √ | √ | x | x | √ | √ | x | √ | √ | S | S | S | S | S | I | S | S | S | S | S |
| J49 | 14/2/2015 | Bronchial aspirate | √ | √ | √ | √ | √ | x | √ | √ | x | x | √ | x | x | √ | √ | S | S | S | S | S | I | S | S | S | S | S |
| J50 | 27/5/2015 | Blood | √ | √ | √ | √ | √ | x | √ | x | x | √ | √ | x | x | √ | x | S | S | S | S | S | I | S | S | S | S | S |
| J51 | 28/5/2015 | Urine | √ | √ | √ | √ | √ | √ | √ | √ | x | x | √ | x | x | √ | √ | S | S | S | S | S | I | I | S | S | S | S |
| J52 | 27/5/2015 | Urine | √ | √ | √ | √ | √ | √ | √ | x | x | √ | √ | √ | √ | √ | x | S | S | S | S | S | I | S | S | S | S | S |
| J53 | 29/5/2015 | Bronchial aspirate | √ | √ | √ | √ | √ | x | √ | √ | x | x | √ | √ | x | √ | x | S | S | S | S | S | S | S | S | S | S | S |

√ indicates presence of virulence genes; x indicates absence of virulence genes; S indicates susceptible; I indicates intermediate; R indicates resistant (according to CLSI guidelines M100-S26).

***P. aeruginosa* archived strains from isolation period 1977 to 1985 (*n* = 52)**

|  |  |  | **Virulence genes** | | | | | | | | | | | | | | | **Antimicrobial susceptibility** | | | | | | | | | | |
| --- | --- | --- | --- | --- | --- | --- | --- | --- | --- | --- | --- | --- | --- | --- | --- | --- | --- | --- | --- | --- | --- | --- | --- | --- | --- | --- | --- | --- |
| **Strain** | **Year received** | **Source of specimen** | ***apr*** | ***lasB*** | ***phzI*** | ***phzII*** | ***phzH*** | ***phzM*** | ***phzS*** | ***exoS*** | ***exoT*** | ***exoU*** | ***exoY*** | ***pvdA*** | ***pilB*** | ***lecA*** | ***lecB*** | **MEM** | **DOR** | **IMI** | **CAZ** | **PTZ** | **TIM** | **PRL** | **CIP** | **AK** | **CN** | **NET** |
| 1 | 9/9/1982 | Blood | √ | √ | √ | √ | √ | √ | √ | x | x | √ | x | √ | x | √ | x | **R** | S | S | S | **R** | **R** | **R** | S | S | S | S |
| 3 | 26/1/1978 | - | √ | √ | √ | √ | √ | √ | √ | √ | x | x | √ | √ | x | √ | √ | S | S | S | S | S | I | S | S | S | S | S |
| 4 | 20/4/1979 | Urine | √ | √ | √ | √ | √ | √ | √ | x | x | √ | √ | √ | x | √ | x | S | S | S | S | S | I | S | S | S | **R** | **R** |
| 5 | 12/3/1980 | Urine | √ | √ | √ | √ | √ | √ | √ | x | x | √ | x | x | x | √ | x | S | S | I | S | S | **R** | S | S | S | S | S |
| 6 | 15/8/1979 | Nasal swab | √ | √ | √ | x | √ | √ | √ | √ | x | x | x | √ | x | x | √ | S | S | S | S | S | I | S | S | S | S | S |
| 7 | 29/11/1976 | - | √ | √ | √ | x | √ | √ | √ | x | x | √ | √ | x | x | √ | x | S | S | S | S | **R** | **R** | **R** | S | S | **R** | **R** |
| 9 | 26/1/1978 | - | x | x | x | x | x | x | x | x | x | x | x | x | √ | x | x | S | S | S | S | S | I | S | S | S | S | S |
| 10 | 20/4/1979 | Ear swab | √ | √ | √ | √ | √ | √ | √ | x | x | √ | √ | x | x | √ | x | S | S | S | S | S | I | S | S | S | S | S |
| 11 | 10/7/1981 | Urine | √ | √ | x | x | √ | x | √ | x | x | √ | √ | x | x | √ | x | S | S | S | S | S | S | S | S | S | S | S |
| 12 | 28/6/1979 | Urine | √ | √ | √ | √ | √ | √ | √ | √ | x | √ | √ | √ | x | √ | √ | S | S | S | S | I | **R** | **R** | S | S | **R** | **R** |
| 13 | 1/12/1980 | Ear swab | x | x | x | x | x | x | x | x | x | x | x | x | x | x | x | **R** | **R** | S | S | S | **R** | S | S | S | S | S |
| 14 | 10/3/1980 | Ear swab | x | x | x | x | x | x | x | x | x | x | x | x | x | x | x | **R** | **R** | S | S | S | I | S | S | S | S | S |
| 15 | 26/1/1978 | - | x | x | x | x | x | x | x | x | x | x | x | x | √ | x | x | S | S | S | S | S | I | S | S | S | S | S |
| 17 | 15/8/1979 | Urine | √ | √ | √ | √ | √ | √ | √ | √ | x | x | √ | √ | x | √ | √ | S | S | S | S | S | I | S | S | S | S | S |
| 18 | 9/3/1978 | Blood | √ | √ | √ | √ | √ | √ | √ | √ | x | x | √ | √ | x | √ | √ | S | S | S | S | S | I | S | S | S | S | S |
| 19 | 26/1/1978 | - | √ | √ | √ | √ | √ | √ | √ | √ | x | x | √ | √ | x | √ | √ | S | S | S | S | S | S | S | S | S | S | S |
| 20 | 25/8/1978 | Urine | √ | √ | √ | √ | √ | √ | √ | √ | x | x | √ | √ | x | √ | √ | S | S | S | S | S | I | S | S | S | S | S |
| 21 | 26/1/1978 | - | √ | √ | √ | √ | √ | √ | √ | √ | x | x | √ | x | x | √ | √ | S | S | S | S | S | I | S | S | S | S | S |
| 22 | 25/8/1978 | Ear swab | x | x | x | x | x | x | x | x | x | x | x | x | x | x | x | **R** | **R** | S | S | S | I | S | S | S | S | S |
| 23 | 12/10/1978 | Wound swab | √ | √ | √ | √ | √ | √ | √ | x | x | x | x | x | x | √ | x | S | S | S | S | S | I | S | S | S | S | S |
| 24 | 25/8/1978 | Wound swab | √ | √ | √ | √ | √ | √ | √ | √ | x | x | √ | √ | x | √ | √ | **R** | S | **R** | S | S | **R** | S | S | S | S | S |
| 25 | 24/2/1978 | - | √ | √ | √ | √ | √ | √ | √ | x | x | √ | √ | √ | x | √ | x | S | S | S | S | S | I | S | S | S | S | S |
| 26 | 11/8/1978 | Urine | √ | √ | √ | √ | √ | √ | √ | x | x | x | √ | x | x | √ | x | S | S | S | S | S | I | S | S | S | S | I |
| 27 | 23/12/1977 | Eye discharge | √ | √ | √ | √ | √ | √ | √ | x | x | √ | √ | x | x | √ | x | S | S | S | S | S | I | S | S | S | S | S |
| 29 | 23/12/1977 | Pus | √ | √ | √ | √ | √ | √ | √ | √ | x | x | √ | x | x | √ | √ | S | S | S | S | S | I | S | S | S | S | S |
| 30 | 26/1/1978 | - | x | √ | √ | √ | √ | √ | √ | x | x | √ | √ | x | x | √ | x | S | S | S | S | S | I | S | S | S | S | S |
| 31 | 27/9/1977 | Pus (bronchi) | √ | √ | √ | √ | √ | √ | √ | √ | x | x | √ | x | x | √ | √ | S | S | S | S | S | S | S | S | S | S | S |
| 32 | 23/12/1977 | Pus (ulcer) | x | x | x | x | x | x | x | x | x | x | x | x | x | x | x | S | S | S | S | S | I | S | S | S | S | S |
| 34 | 23/12/1977 | Peritoneal fluid | x | x | x | x | x | x | x | x | x | x | x | x | x | x | x | S | S | S | S | S | S | S | S | S | S | S |
| 35 | 23/12/1977 | Urine | √ | √ | √ | √ | √ | √ | √ | x | x | √ | √ | x | x | √ | x | S | S | S | S | S | **R** | I | S | S | S | S |
| 37 | 1/12/1980 | Urine | √ | √ | √ | √ | √ | √ | √ | √ | x | x | √ | x | x | √ | √ | S | S | S | S | S | S | S | S | S | S | S |
| 38 | 13/3/1978 | Urine | √ | x | √ | x | x | √ | x | √ | x | x | x | √ | x | √ | √ | S | S | S | S | S | I | S | S | S | S | S |
| 39 | 9/11/1984 | Urine | √ | √ | √ | √ | √ | √ | √ | √ | x | x | √ | √ | x | √ | √ | S | S | I | S | **R** | **R** | **R** | S | **R** | **R** | **R** |
| 40 | 17/1/1985 | Urine | √ | √ | √ | √ | √ | x | √ | √ | x | x | √ | x | √ | √ | √ | S | S | S | S | S | I | S | S | S | S | S |
| x5 | 15/3/1978 | - | √ | √ | √ | √ | √ | √ | √ | x | x | √ | √ | √ | √ | √ | x | S | S | S | S | S | I | I | S | S | S | S |
| x9 | 20/3/1979 | Ear swab | x | x | x | x | x | x | x | x | x | x | x | x | x | x | x | **R** | **R** | S | S | S | I | S | S | S | S | S |
| x11 | 10/10/1980 | Ear drainage | √ | √ | √ | √ | √ | √ | √ | √ | x | x | √ | x | x | √ | √ | S | S | S | S | S | I | S | S | S | S | S |
| x16 | 1/12/1980 | Ear swab | √ | √ | √ | √ | √ | √ | √ | √ | x | √ | √ | √ | x | √ | √ | S | S | S | S | S | I | S | S | S | S | S |
| x24 | 10/11/1982 | Urine | √ | √ | √ | √ | √ | √ | √ | x | x | √ | √ | √ | x | √ | x | S | S | S | S | S | I | S | S | S | S | S |
| x71 | 21/6/1983 | Ear swab | √ | √ | √ | √ | √ | √ | √ | x | x | √ | x | x | x | √ | x | S | S | S | S | S | I | S | S | S | S | S |
| x93 | 18/1/1983 | Ear swab | √ | √ | √ | √ | √ | √ | √ | √ | x | x | √ | √ | x | √ | √ | S | S | S | S | S | I | S | S | S | S | S |
| x106 | 22/6/1983 | Ear swab | √ | √ | √ | √ | √ | √ | √ | √ | x | x | √ | √ | x | √ | √ | S | S | S | S | S | I | S | S | S | S | S |
| x117 | 10/2/1983 | Ear swab | x | x | x | x | x | x | x | x | x | x | x | x | x | x | x | **R** | **R** | S | S | S | **R** | S | S | S | S | S |
| x121 | 9/11/1984 | Ear swab | x | x | x | x | x | x | x | x | x | x | x | x | x | x | x | **R** | **R** | S | S | S | **R** | S | S | S | S | S |
| x155 | 9/11/1984 | Ear swab | √ | √ | √ | √ | √ | √ | √ | √ | x | x | √ | √ | x | √ | √ | S | S | S | S | S | I | S | S | S | S | S |
| x188 | 15/1/1985 | Ear swab | √ | √ | √ | √ | √ | x | √ | √ | x | x | √ | √ | x | √ | √ | S | S | S | S | S | I | S | S | S | S | S |
| x196 | 16/1/1985 | Wound swab | √ | √ | √ | √ | √ | x | √ | √ | x | x | √ | √ | x | √ | √ | S | S | S | S | S | I | S | S | I | I | I |
| x199 | 16/1/1985 | Urine | √ | √ | √ | √ | √ | x | √ | x | x | √ | √ | x | x | √ | x | S | S | S | S | S | I | S | S | S | S | S |
| PP48 | 25/10/1983 | Ear swab | √ | √ | √ | √ | √ | x | √ | √ | x | x | √ | √ | x | √ | √ | S | S | S | S | S | I | S | S | S | S | S |
| PP57 | 22/2/1984 | Ear swab | √ | √ | √ | √ | √ | x | √ | √ | x | x | x | √ | x | √ | √ | S | S | S | S | S | I | S | S | S | S | S |
| PS3 | 8/9/1983 | Ear swab | √ | √ | √ | √ | √ | x | √ | √ | x | x | √ | √ | x | √ | √ | S | S | S | S | S | I | S | S | S | S | S |
| PS4 | 30/5/1984 | Tracheal secretion | √ | √ | √ | √ | √ | x | √ | √ | x | √ | √ | √ | x | √ | x | S | S | S | S | **R** | I | **R** | S | S | S | I |

√ indicates presence of virulence genes; x indicates absence of virulence genes; S indicates susceptible; I indicates intermediate; R indicates resistant (according to CLSI guidelines M100-S26).
