# Supplementary figures and images for: Antimicrobial susceptibility and virulence genes of clinical and environmental isolates of Pseudomonas aeruginosa
Source: PeerJ. 2019 Jan 22;7:e6217. doi: 10.7717/peerj.6217 (PMC6346980; doi:10.7717/peerj.6217)

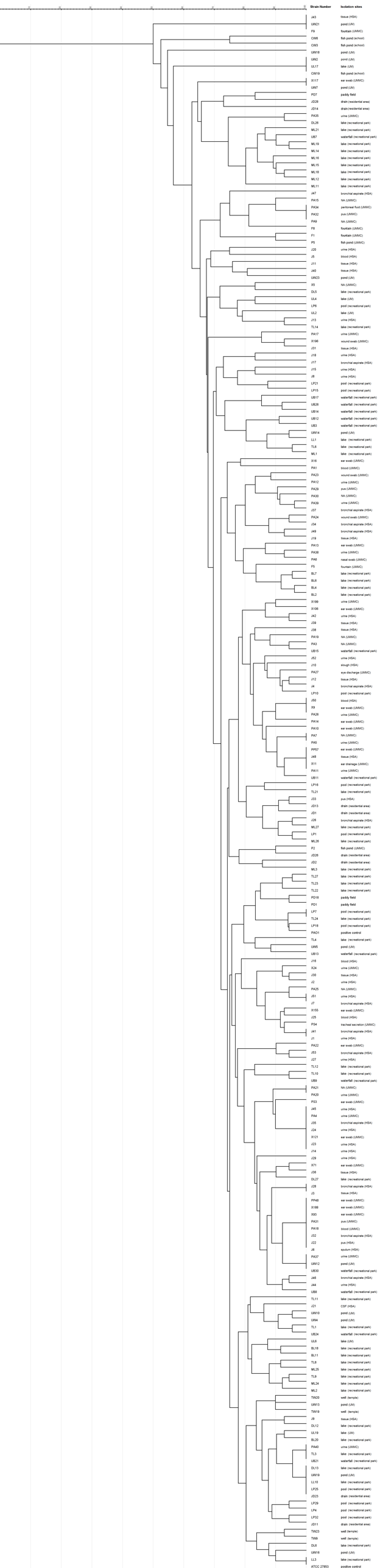

Supplement: Supplemental Information 2 [file peerj-07-6217-s002.pdf]
