# Supplementary material for: Antimicrobial susceptibility and virulence genes of clinical and environmental isolates of Pseudomonas aeruginosa
Source: PeerJ. 2019 Jan 22;7:e6217. doi: 10.7717/peerj.6217 (PMC6346980; doi:10.7717/peerj.6217)
Supplement: Supplemental Information 3 — Notes: NA source of specimen not available. Significant P-values are shown in bold. [file peerj-07-6217-s003.docx]

**Supplementary file 3: Detection of *P. aeruginosa* from isolation periods 1977 to 1985 and 2015 in various specimens**

|  | No. of clinical isolate (%) | |  |
| --- | --- | --- | --- |
| Specimen | **1977−1985** | **2015** | ***P*-value** |
| Urine | 14 (26.9) | 17 (32.1) | 0.5628 |
| Blood | 2 (3.8) | 4 (7.5) | 0.4140 |
| Bronchial aspirate | 0 (0) | 14 (26.4) | **0.0001** |
| Tracheal secretion | 1 (1.9) | 0 (0) | 0.3104 |
| Sputum | 0 (0) | 1 (1.9) | 0.3196 |
| Pus | 3 (5.8) | 2 (3.8) | 0.6312 |
| Tissue | 0 (0) | 13 (24.5) | **0.0001** |
| Swab (ear, nasal, wound) | 20 (38.5) | 0 (0) | **< 0.00001** |
| CSF | 0 (0) | 1 (1.9) | 0.3196 |
| Slough | 0 (0) | 1 (1.9) | 0.3196 |
| Peritoneal fluid | 1 (1.9) | 0 (0) | 0.3104 |
| Discharge/drainage (ear, eye) | 2 (3.8) | 0 (0) | 0.1494 |
| NA | 9 (17.4) | 0 (0) | **0.0015** |
| TOTAL | 52 (100) | 53 (100) |  |

Notes: NA source of specimen not available. Significant *P*-values are shown in bold.
