# Supplementary material for: Antimicrobial susceptibility and virulence genes of clinical and environmental isolates of Pseudomonas aeruginosa
Source: PeerJ. 2019 Jan 22;7:e6217. doi: 10.7717/peerj.6217 (PMC6346980; doi:10.7717/peerj.6217)

**Sequencing of virulence genes of *P. aeruginosa***

**Alkaline protease (*apr*)**

>1988147_apr_apr_F

CGAGCGTATACCAGGTAGACACTTCCTGCATGCCTATGCGCGGGGCGGGGACGAATTGGTCAATGGCCATCCGTCCTATACCGTCGACCAGGCGGCGGAGCAGATCCTCCGCGAACAGGCGTCTTGGCAGAAAGCGCCGGGCGACTCGGTGCTGACCCTGTCCTATTCGTTCCTGACCAAACCGAACGACTTCTTCAATACGCCGTGGAAGTATGTCAGCGATATCTACTCGCTGGGCAAGTTCAGCGCCTTTTCCGCGCAGCAGCAGGCCCAGGCCAAGTTGTCGCTGCAATCCTGGTCGGACGTCACCAATATCCACTTCGTCGACGCCGGCCAGGGCGATCAGGGCGACCTGACCTTCGGCAACTTTAGCAGTAGTGTCGGCGGTGCGGCGTTCGCCTTCCTGCCGGATGTACCGGATGCGCTCAAGGGGCAATCCTGGTACCTGATCAACAGCAGCTACAGCGCCAACGTCAATCCGGCCAACGGCAACTACGGACGCCAGACCCTGACCCACGAGATCGGCCATACCCTGGGCCTGAGCCACCCCGGCGACTACAACGCCGGCGAGGGCGATCCCACCTACGCCGACGCTACCTACGCCGAGGACACCCGCGCCTATTCGGTGATGAGCTACTGGGAAGAGCAGAACACCGGCCAGGACTTCAAGGGCGCCTATTCCTCGGCACCGCTGCTGGACGACATCGCGGCGATCCAGAAGCTCTACGGGGCCAACCTGACCACCCGCACCGGCGACACGGTGTACGGCTTCAACTCCAACACCGAGCGCGACTTCTACAGCGCCACCTCGTCCAGTTCCAAGCTGGTGTTCTCGGTGTGGGACGCCGGCGGCAACGACACCCTGGACTTCTCCGGCTTCAGCCAGAACCAGAAGATCAACCTCAACGAGAAGGCGCTGTCCGATGTCGGCGGGTTGAAGGGCAATGTGTCGATCGCTGCCGGGTCCGCGGGGAAAAAA

>1988148_apr_apr_R

TTGACTTCACCCGCCGACATCGGACAGCGCCTTCTCGTTGAGGTTGATCTTCTGGTTCTGGCTGAAGCCGGAGAAGTCCAGGGTGTCGTTGCCGCCGGCGTCCCACACCGAGAACACCAGCTTGGAACTGGACGAGGTGGCGCTGTAGAAGTCGCGCTCGGTGTTGGAGTTGAAGCCGTACACCGTGTCGCCGGTGCGGGTGGTCAGGTTGGCCCCGTAGAGCTTCTGGATCGCCGCGATGTCGTCCAGCAGCGGTGCCGAGGAATAGGCGCCCTTGAAGTCCTGGCCGGTGTTCTGCTCTTCCCAGTAGCTCATCACCGAATAGGCGCGGGTGTCCTCGGCGTAGGTAGCGTCGGCGTAGGTGGGATCGCCCTCGCCGGCGTTGTAGTCGCCGGGGTGGCTCAGGCCCAGGGTATGGCCGATCTCGTGGGTCAGGGTCTGGCGTCCGTAGTTGCCGTTGGCCGGATTGACGTTGGCGCTGTAGCTGCTGTTGATCAGGTACCAGGATTGCCCCTTGAGCGCATCCGGTACATCCGGCAGGAAGGCGAACGCCGCACCGCCGACACTACTGCTAAAGTTGCCGAAGGTCAGGTCGCCCTGATCGCCCTGGCCGGCGTCGACGAAGTGGATATTGGTGACGTCCGACCAGGATTGCAGCGACAACTTGGCCTGGGCCTGCTGCTGCGCGGAAAAGGCGCTGAACTTGCCCAGCGAGTAGATATCGCTGACATACTTCCACGGCGTATTGAAGAAGTCGTTCGGTTTGGTCAGGAACGAATAGGACAGGGTCAGCACCGAGTCGCCCGGCGCTTTCTGCCAAGACGCCTGTTCGCGGAGGATCTGCTCCGCCGCCTGGTCGACGGTATAGGACGGATGGCCATTGACCAATTCGTCCCCGCCCCGCGCATAGGCATGCAGGAAGTTGTCTACCTGGGTATACGCATCGCTACGACCTTTCAATGCAGAGATTTTGCTGGAACA


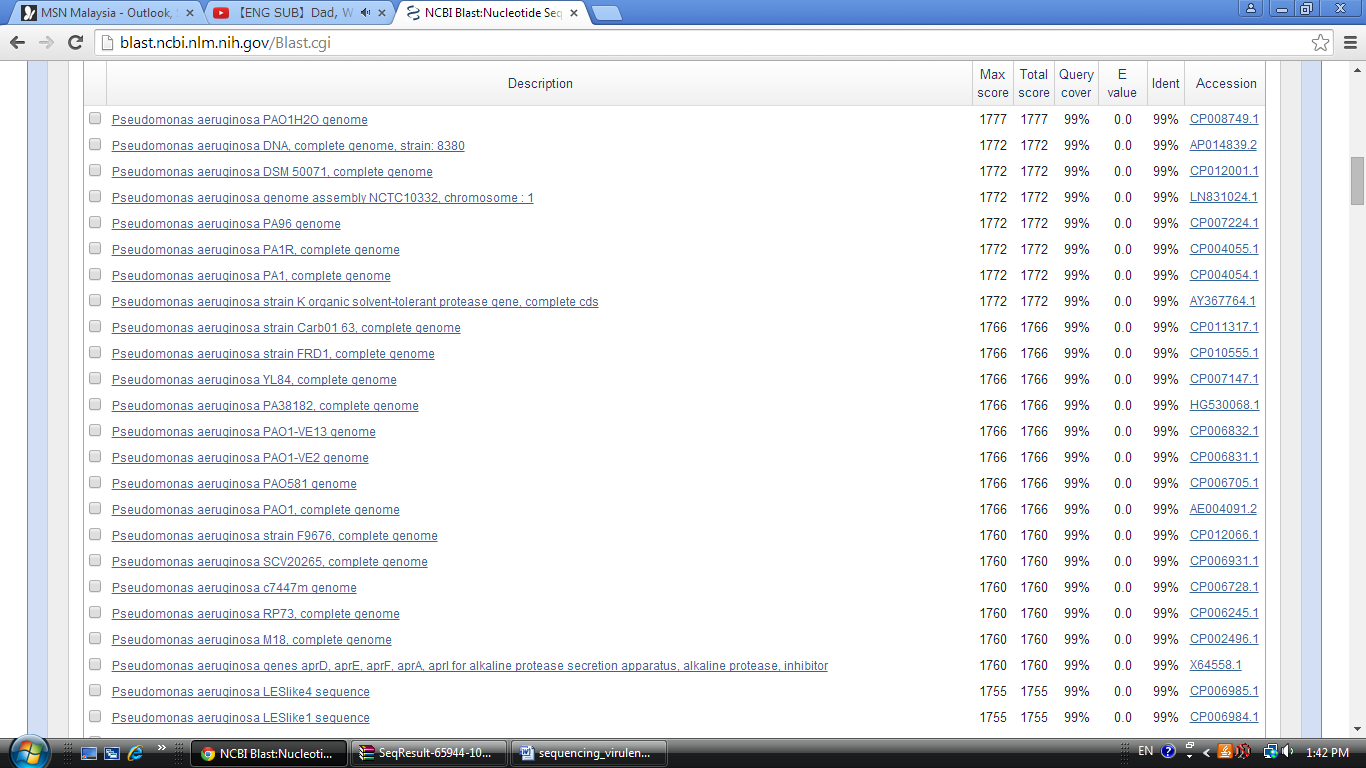


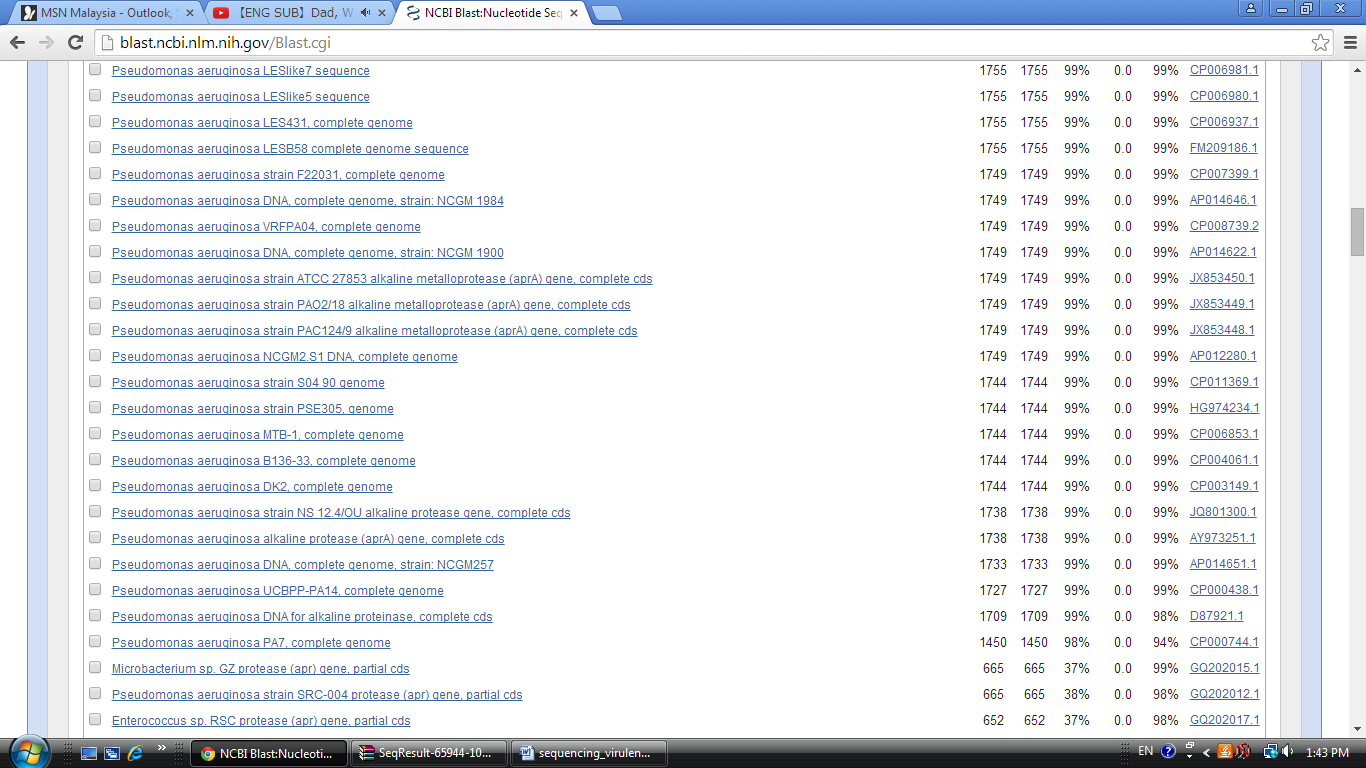


**Elastase B (*lasB*)**

>1988149_lasB_lasB_F

GTGGATGTGAGTCGATGCCGTTGTAGTACTGCGACGCGTTGTCGATGGATCGCCCGTCGCGGCTGGGCTGGTCCATGTAGCGCAGCGCACCGCTGCCCTTCTTGATGTCGTAGCCGATCAGGAAGTCGTTCTTGCCGCGCATATAGAACTCGGCAGCCTCGCCGGCCATGTCGGAGAACGCTTCGTTCATTCCGCCTGATTGCCCGCGGTAGATCAGCCCGGAGTTCTGCTCGGTGAAGCCGTGGCTGACCTCGTGGGCCGCCACGTCCAGCGACACCAGCGGATAGAACATGGTGGCGCCGTCGCCGAAGAGCATCGCCGTGCCGTCCCAGTAGGCGTTCTCCACGCTGCGCCCGTAGTGCACCTTCATGTACAGCTTGTGGGTCAGCGGGCTGGTGCCGAACCAGTCCCGGTACAGTTTGAACACCACGCCGCCGAAGAAATGCGCGTCGTTCAGCGGCGAATAGGCGCCGTTGACCTGCTTGTAGGTGTTGGTCGGGCAGGCGAAGCGGAACGGCGTGGTCTTGCTGTCGTCGGTGCTGCTGTTCATGTCGACGGTGATGACGTTGCCGTCGTCCATCTCGCAGCGGTCGTTGACGATCAGCGGACCGTAGTCGCTACCGTAGGTGTACTTGCCGATCTTCTGGTTGCCGCCGGGGCCGCCCGCCTCGGCGTGGGCCAGGCCTTCCCACTGATCGAGCACTTCGCCGGTCTTGGCGTCGATGACGAAATGCGGCCGCGACAGTCCCTCGCCGGGAATCAGGTAGGAGACGTTGTAGACCAGTTGGGCGATGTTGTTCTCGCCCAGGCGGATCACCAGTTCCACTTTGTCATTCTCGGTCTTGCGGCCCTGGGCCTTCAGGCTCTTGGCCTGGGCCAGCACCTGCTCGGCGGATACCGCCGCGGTGGTGCTGCCCGGCAGGTCGGCAGCGATGTTGGCGACGAAATGGCCGCTGCGCTGCGCCGCCACGCTCTTGCCGGGACCCTTGACTTCGGTGATGGCTTCGCCGACCACCCGTACGCCGTTGTGGAATTGCTCGTAGCGGGTGACCTGCTTGCCGTTGGGCAGGGTCGTGCTGCGGATCGCTTTCAGTTCGTCGGCACCGCCAGCGCCAACCGCGGCTTGCAAGGTGACCGGGCCGGNCCCGCCCTGGGCAACCTTGCTGGGATTTGTGGAACACACTCCAATATAAA

>1988150_lasB_lasB_R

ACCGTCATGACGCGACGGTCGGTCACCTTGCAGCCGCGGTCGGCGCTGGCGGTGCCGACGAACTGAAAGCGATCCGCAGCACGACCCTGCCCAACGGCAAGCAGGTCACCCGCTACGAGCAATTCCACAACGGCGTACGGGTGGTCGGCGAAGCCATCACCGAAGTCAAGGGTCCCGGCAAGAGCGTGGCGGCGCAGCGCAGCGGCCATTTCGTCGCCAACATCGCTGCCGACCTGCCGGGCAGCACCACCGCGGCGGTATCCGCCGAGCAGGTGCTGGCCCAGGCCAAGAGCCTGAAGGCCCAGGGCCGCAAGACCGAGAATGACAAAGTGGAACTGGTGATCCGCCTGGGCGAGAACAACATCGCCCAACTGGTCTACAACGTCTCCTACCTGATTCCCGGCGAGGGACTGTCGCGGCCGCATTTCGTCATCGACGCCAAGACCGGCGAAGTGCTCGATCAGTGGGAAGGCCTGGCCCACGCCGAGGCGGGCGGCCCCGGCGGCAACCAGAAGATCGGCAAGTACACCTACGGTAGCGACTACGGTCCGCTGATCGTCAACGACCGCTGCGAGATGGACGACGGCAACGTCATCACCGTCGACATGAACAGCAGCACCGACGACAGCAAGACCACGCCGTTCCGCTTCGCCTGCCCGACCAACACCTACAAGCAGGTCAACGGCGCCTATTCGCCGCTGAACGACGCGCATTTCTTCGGCGGCGTGGTGTTCAAACTGTACCGGGACTGGTTCGGCACCAGCCCGCTGACCCACAAGCTGTACATGAAGGTGCACTACGGGCGCAGCGTGGAGAACGCCTACTGGGACGGCACGGCGATGCTCTTCGGCGACGGCGCCACCATGTTCTATCCGCTGGTGTCGCTGGACGTGGCGGCCCACGAGGTCAGCCACGGCTTCACCGAGCAGAACTCCGGGCTGATCTACCGCGGGCAATCAGGCGGAATGAACGAAGCGTTCTCCGACATGGCCGGCGAGGCTGCCGAGTTCTATATGCGCGGCAAGAACGACTTCCTGATCGGCTACGACATCAAGAAGGGCAGCGGTGCGCTGCGCTACATGGACCAGCCCAGCCGCGACGGGCGATCCATCGACAACGCGTCGCAGTACTACAACGGCATCGACGTGCACCATTCCAGCGGGGTGACAACGGGGGGGCTTCCCNACGTTAAAAC


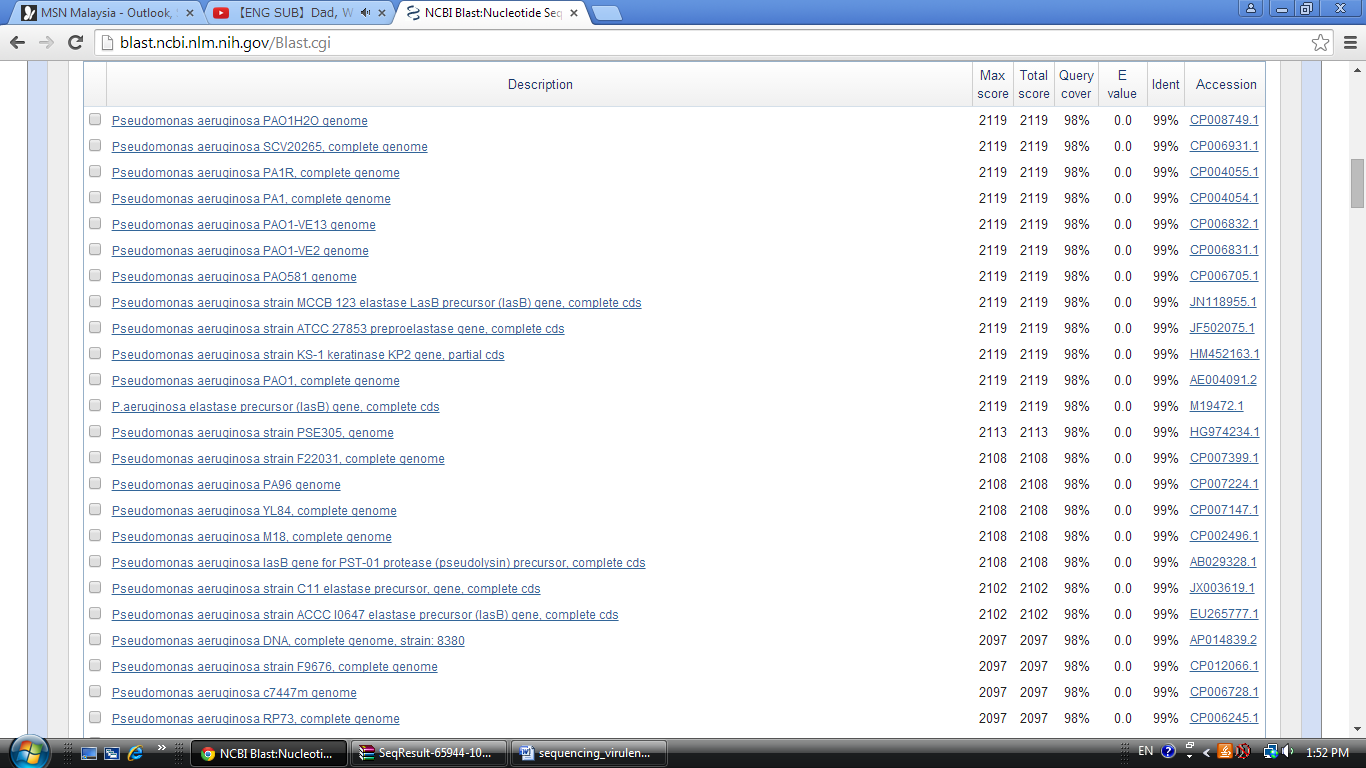


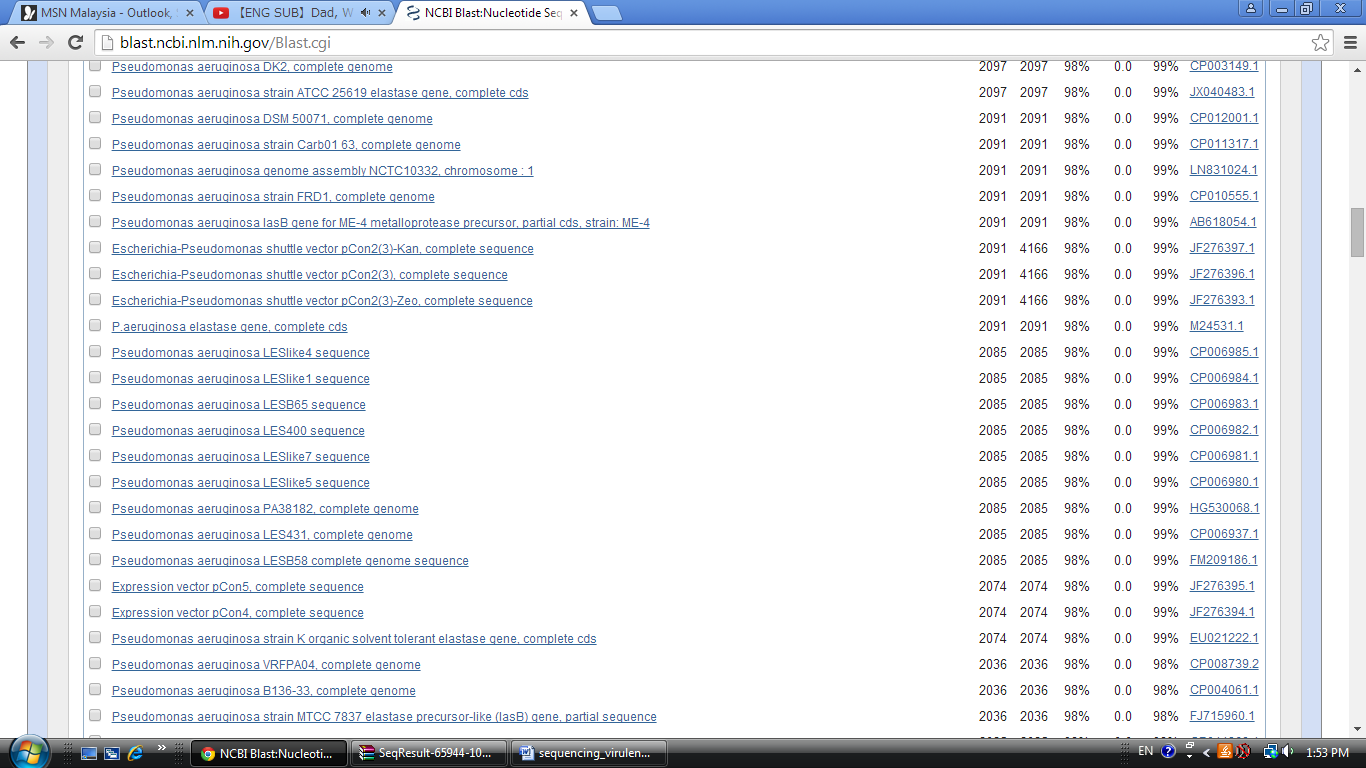


**Phenazine operon (*phzI*)**

>1988151_phzI_phzI_F

AGGAATCTTGACTACAGATCTTGTAGTTGAGCCGGTACGAGCGTTCTGTGTTTTATGCAATCCACATCAGCGACCAGGGATGCTGGCTATTTGAAACACTTCACGGAATGACGCTGAAAGTCTTCGCGACCTCGTCTGTCGCACCTTAACGAAAGCATTGCGAATCCATTACCGACAGGTTTCCAAAAGAAACCCGGGATGAAACTCCTATTGCCTTTCGAAAATTGGAAACGACAGGCGAACATATGTAACGCGAAATTTCACCCTACGTATAAACAATGCGCCCAGCGAATATCGCTCCCTTACCGAGCGACGAACTCCTGCGCGCCAGCGAATAACCGATGCCGCGAGGGAAAAGTTTCTCCGA

>1988152_phzI_phzI_R

ATCGCTGGCGCGCAGGAGTTCGTCGCTCGGTAAGGGAGCGATATTCGCTGGGCGCATTGTTTATACGTAGGGTGAAATTTCGCGTTACATATGTTCGCCTGTCGTTTCCAATTTTCGAAAGGCAATAGGAGTTTCATCCCGGGTTTCTTTTGGAAACCTGTCGGTAATGGATTCGCAATGCTTTCGTTAAGGTGCGACAGACGAGGTCGCGAAGACTTTCAGCGTCATTCCGTGAAGTGTTTCAAATAGCCAGCATCCCTGGTCGCTGATGTGGATTGCATAAAACACAGAACGCTCGTACCGGCTCAACTACAAGATCTGGTAGGTGCCAGACAGGGTATGCGGGATTGCTAAGCTGATGA


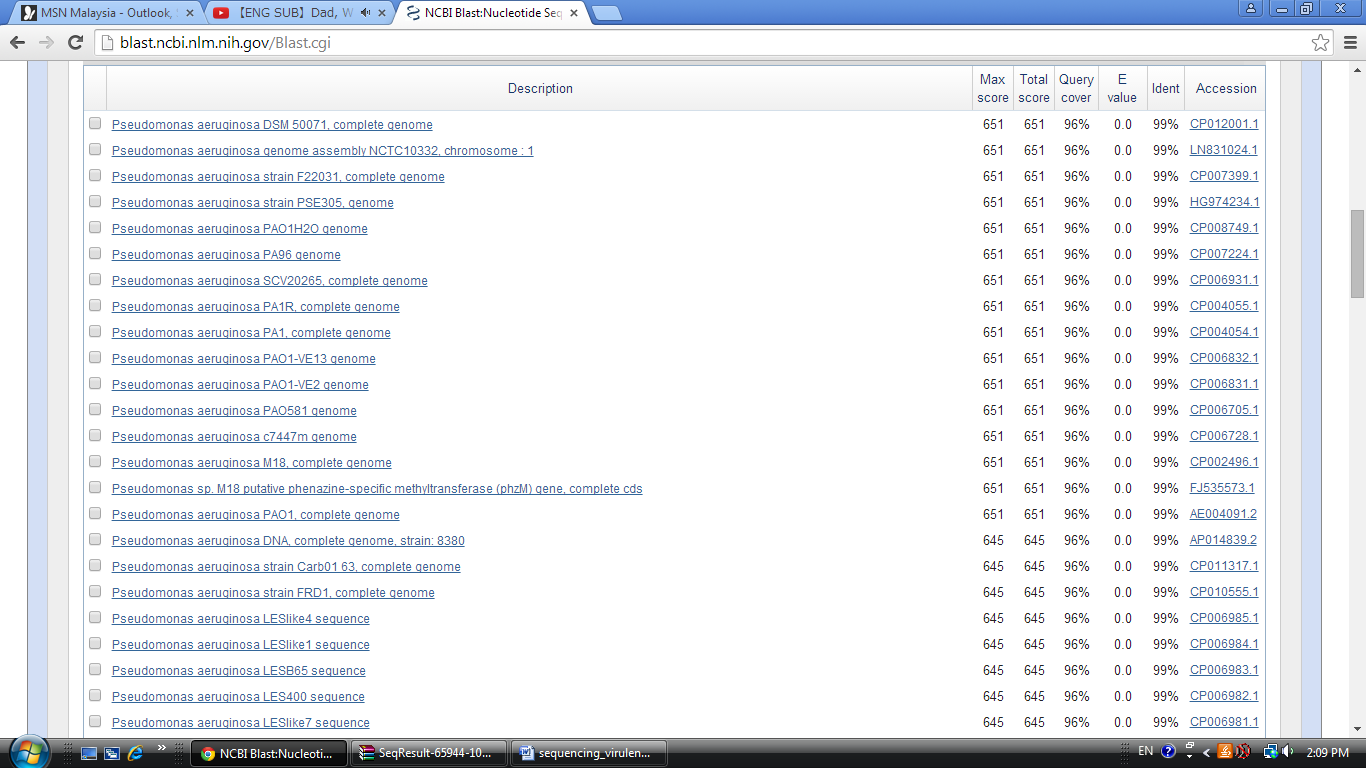


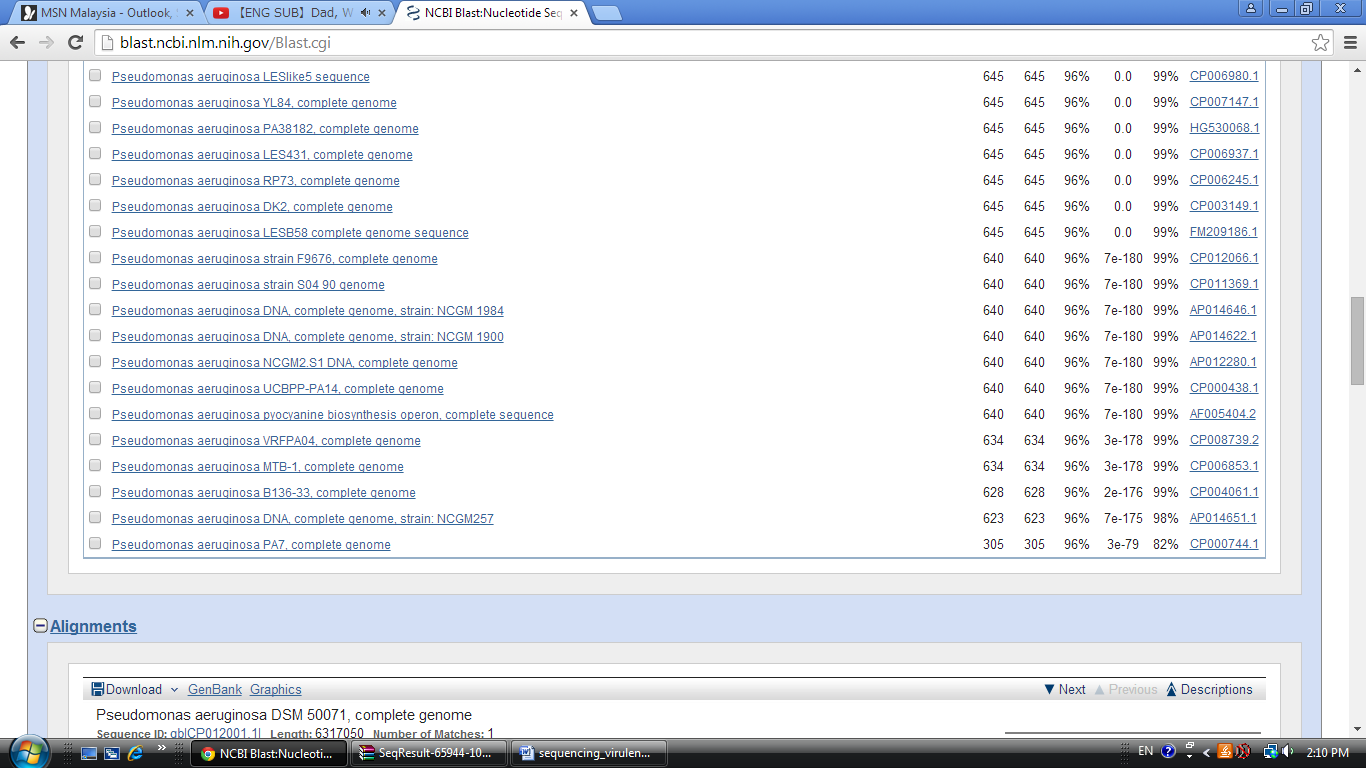


**Phanazine operon (*phzII*)**

>1988153_phzII_phzII_F

GTCCACGATGGGAGCGTGCGAGAGACATGAGAAAGACCGCCGTGAGGCCCATCGGAGAGCCGTTCTACGGTTTCCGCAAAGATCCGGGGCGCCGTCCCCTCCAGCCCAGCGCAGTTCCTGCGCGGCGCCTCGTGTCCGTGCTCATCGAGAAGTTCTCTTCAGCCTCGTTTCGTCGTCGCCCGGCGGGCGGCGAATGGGCTCGACCTCGTCCGGAACACCCGCACAGGGCCGGTGGCGATATGTACTTCCAGGTCCGGCTTGATAAAGGGAATTGTCATGAGTGGATAAGACGGAAACAAAAAAGAATAAAAACGCTGAAGAACCGAATCCTGCCGGGATCGATTGTTGACTGGTGAAGCTGGCATGCATGATGAGAGAGAGGGATATCTCGAGATTTTGTCAAGAATAACAACCGAGGAAGAGTTCTTCTCCCTGGTTCTCGAGATATGCGGTAATTATGGATTCGAATTCTTTTCATTCGGTGCGCGGGCGCCTTTCCCGCTGACCGCACCTAAATATCATTTCCTGTCCAATTACCCAGGGGAATGGAAAAGCAGATATATCTCCGAAGACTACACATCCATCGACCCGATCGTGCGCCATGGTCTCCTGGAATACACCCCGCTGATCTGGAATGGCGAAGACTTCCAGGAGAACCGTTTCTTCTGGGAGGAAGCGCTGCATCACGGTATCCGTCACAGCTGGTCCGATCCTCGGTGCCGCGACAAGTACGGGCATGATCAGCATGGCTGTCCCCTGGGTGCGTTTCCAGCGAAGAGCATCGACCGCCACGGGAAATCCTGGCAGAAGGAATCCTATCCTGCTTCTGGATCACCAGTCATGCTTGCAGGGCTACCTTCGGAGGACCTGGCTGGACGCCACTGCATCCGTCCCTGGAAAGCAAATGTGGCGCCCTGAACCGCCCAGGGGAAACCCGAGAATGACTCAAAGTGGCACCAGCGGGTGGGGCCAAGAACCTTACTGGCGTAGATTCGGGCCTTGATCCCTGGTCTAATCGAACCAGGCGCAACGGGAGAAAGTTCCATAACTGG

>1988154_phzII_phzII_R

GGTGCTATGATCGAAGGATCAGGCCGATCTCGCCGTAGGTCTTGCCCACCGCGGTCCACTTGAGCATCTCGGTTTCCCTGGCGGTCAGGCGCACATTGCTTTCCGGGACGATGCGCGGCGCCAGCAGGTCGCCGAAGGTAGCCTGCAGCATGCTGGTGATCCAGAGCAGGAAGGATTCCTTCTCCAGGATTTCCGTGGCGGCGATGCTCTCGCTGGAACGCACCAGGGACAGCATGCTGATCAGCCCGTACTTGCCGCGGACCGGGATCGACCAGCCGTGACGGATGCCGTGATGCAGCGCTTCCTCCCAGAAGAAACGGTTCTCCTGGAAGTCTTCGCCATTCCAGATCAGCGGGGTGTATTCCAGGAGACCATGGCGCACGATCGGGTCGATGGATGTGTAGTCTTCGGAGATATATCTGCTTTTCCATTCCCCTGGGTAATTGGACAGGAAATGATATTTAGGTGCGGTCAGCGGGAAAGGCGCCCGCGCACCGAATGAAAAGAATTCGAATCCATAATTACCGCATATCTCGAGAACCAGGGAGAAGAACTCTTCCTCGGTTGTTATTCTTGACAAAATCTCGAGATATCCCTCTCTCTCATCATGCATGCCAGCTTCACCAGTCAACAATCGATCCCGGCAGGATTCGGTTCTTCAGCGTTTTTATTCTTTTTTGTTTCCGTCTTATCCACTCATGACAATTCCCTTTATCAAGCCGGACCTGGAAGTACATATCGCCACCGGCCCTGTGCGGGTGTTCCGGACGAGGTCGAGCCCATTCGCCGCCCGCCGGGCGACGACGAAACGAGGCTGAAGAGAACTTCTCGATGAGCACGGACACGAGGCGCCGCGCAGGAACTGCGCTGGGCTGGAGGGGACGGCGCCCCGGATCTTTGCGGAAACCGTAGAACGGCTCTCCGATGGGCCTCACGGCGGTCTTTCTCATTGTTCTTCTCGCACGCTCCATCGTCGTCGGGAGAGCCTCCCACACAAAAAC


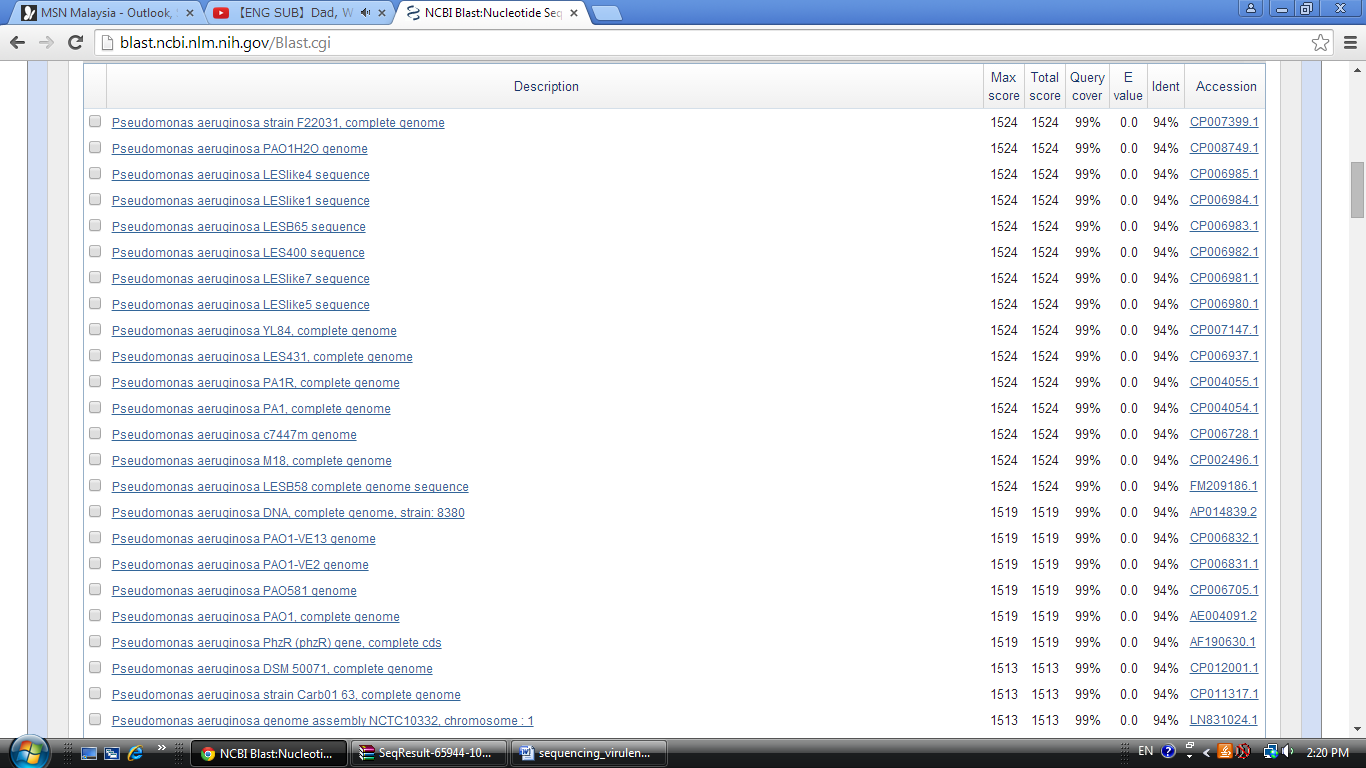


**Phenazine modifying enzyme (*phzH*)**

>1988155_phzH_phzH_F

AGGAGATTGCTGCGATCTTCGCCATGACCGATACGCTCGCCTTGCGCGGGCCGGATGCCGAGGGCATCTGGAAGCACCGCAACGCCCTGCTGGGTCACCGGCGGCTGGCGGTCATCGACCTCAGCGGCGGCGTGCAGCCGATGTCCTATCGCTTTCCCACCGGCCAGGAGGTCACCCTCGTCTACACCGGCGAGGTGTACAACCACGATGCCCTGCGCGAGCGGTTGCGCCGGGCCGGACATGAGTTCCGCACCCGCAGCGATACCGAGGTGGTCCTGCACGCCTATCTGCAATGGGGCGAGCGTTGTTGCGAGTACCTGACCGGGATGTTCGCCTTCGCCGTCTTCGATGGCCGCGACGGCCACCTGCTGCTGGTGCGCGACCGCCTGGGCATCAAGCCGCTGTATTACGCGCGGCACCGCGAGGGACTGCTGTTCGGCTCGGAGATCAAGTCCATCCTGGCGCATCCGGAATTCGCCGCCAGGCTCGACGCGGTCGGCCTGGTCGACCTCCTGACGCTGTCCCGGGGCACTTCGCAGACGCCGTTCCGCGAGGTCCAGGAACTGCTGCCCGGCCACCTGCTGTCCTGGCGTCCCAATTCCCAGGCGAAGTTGCGCCGCTATTGGGAGGTACGCCGCCAGGAGCATGCCGACGACCTGCAGAGCACCGTGCAGCGCACCCGCGAACTGGTCACCCGCGCCCTGGGGGCGCAATTGCACGCCGACGTTCCGGTGTGTTCGCTGCTATCGGGTGGGCTCGATTCGACCGCCCTGACCGGCATCGCCCAGCGCATCGCGAAGGCGGAGCACGGCGGCGACATCAATTCGTTCTCGGTGGACTTCGTCGGCCAGGCCGAGCAGTTCCGCAGCGACGACCTGCGTCCCGACCAGGACCAGCCGTTCGCCCTGCTGGCCGCGCAGTACATCGGCAGCCGTCATCGCACCGTGCTCATCGACAATGCCGAACTGGTCTGCGAACGAGCGCGCGAAGAGGTATTCCGGGCCAAGGACGTACCTTCCACCTTCGGCGACATGGATACCTCGCTGCACCTGATGTTCGGCGAGATCCGCCGGCATTCCACGGTGGCCATCTCCGGTGAAGGCGCCGATGAACTGTTCGGTGGCTACGGCTGGTTCCCCGATCCCCAGGCGGTGGCTGCGGGCCGCTTCCCCTGGGCCTCCAGGGGGCGCCTGCCGGCCGGGTTTATTGAAGGCGGGTTTCAACGGCNGCTGGGGATTCCCTCCAGTAACAGCAAGGCCAACNTCCAACAAAGGGTTTGGCCCAGGTCTAAACCTNGGGCCGGAAATTCCCTGAGNAACCGGTGATGGCCTAGNTTTCGCCCCTGNNCTTTAAANCCTNGGAAGGGCTGGCGNTCCCTANACAGATGATCCCCTGTAAATTGTCGAGGNGCCTTGAGAGTGNGGGTGCCCAACTCTACATTGATATGGTGGTAAATNNTACAAATATGCCCNGGTAGCATATAACCATGGGAACACGAATATATGGCCCCTCCACAGGCGCTTCCCGCATATTGCTCCTA

>1988156_phzH_phzH_R

GGTCTCGATCGGCGGCAGGACTCTCGCGAAACGATGCCGAACACCGGGTTCGCCGCGTCCTCCAGCAGGCGCCGCACGCTCCCGCGCAGGAAACGCTCGTAGCCGAGGTTGGCAGAAGTCGGATAAGGGCTCTTGCGGCGCTTGAGCACGGCTTCCGGGACATAGTCGGCGCAGGCCCGCTTGAGCAGCCACTTCTCCTCGCCGTCCCGGCTCTTGATCGACCAGGGCACGTTGTAGACGTACTCCACCAGCTCATGGTCGGTGTAGGGCACCCGCACCTCCAGGCCGTTGCACATGCTCAGGCGATCCTTGCGTTCGAGCAGCAGCACCATCCAGCGCTTCAGATGCAGGTGGCTGAACTCGCGCATCCGCCGCTCCTCCGGGCTGTCGCCGGCCAGGTGTTCGACCTGGCGCAGCCCATCGTCGTAGCTGGCCTGCTGGTACTGGAGGAGATCGCAGCGGCGGTTGAAACCGGCGTCGATGAAGCCGGCCGGCAGGCGCACCCTGGAGGCCCAGGGGAAGCGCGCCGCAGCCACCGCCTGCGGATCGCGGAACCAGCCGTAGCCACCGAACAGCTCATCGGCGCCTTCACCGGAGATGGCCACCGTGGAATGCCGGCGGATCTCGCCGAACATCAGGTGCAGCGAGGTATCCATGTCGCCGAAGGTGGAAGGTACGTCCTTGGCCCGGAATACCTCTTCGCGCGCTCGTTCGCAGACCAGTTCGGCATTGTCGATGAGCACGGTGCGATGACGGCTGCCGATGTACTGCGCGGCCAGCAGGGCGAACGGCTGGTCCTGGTCGGGACGCAGGTCGTCGCTGCGGAACTGCTCGGCCTGGCCGACGAAGTCCACCGAGAACGAATTGATGTCGCCGCCGTGCTCCGCCTTCGCGATGCGCTGGGCGATGCCGGTCAGGGCGGTCGAATCGAGCCCACCCGATAGCAGCGAACACACCGGAACGTCGGCGTGCAATTGCGCCCCCAGGGCGCGGGTGACCAGTTCGCGGGTGCGCTGCACGGTGCTCTGCAGGTCGTCGGCATGCTCCTGGCGGCGTACCTCCCAATAGCGGCGCAACTTCGCCTGGGAATTGGGACGCCAGGACAGCAGGTGGCCGGGCAGCAGTTCCTGGACCTCCCGGAACGGCGTCTGCCAAATGCCCCGGGACAACCTTCAGGAGGTCGACCAAGGCCAACCGGTTCCAGCCTGGCGGCNAATTCCGGATGGCCCAGGATGGACTTGATCTCCGAGCCGAAAAGAGTTCCTTCCGGGGGCCGCCGGTAATACATGGGCTGTATGCCCAGGCGGTCCGCACTAAAAAAAGGTGGGCCGTCCGGGCCCTCAAAAAAGGGAAAGGGATAATTCCNG


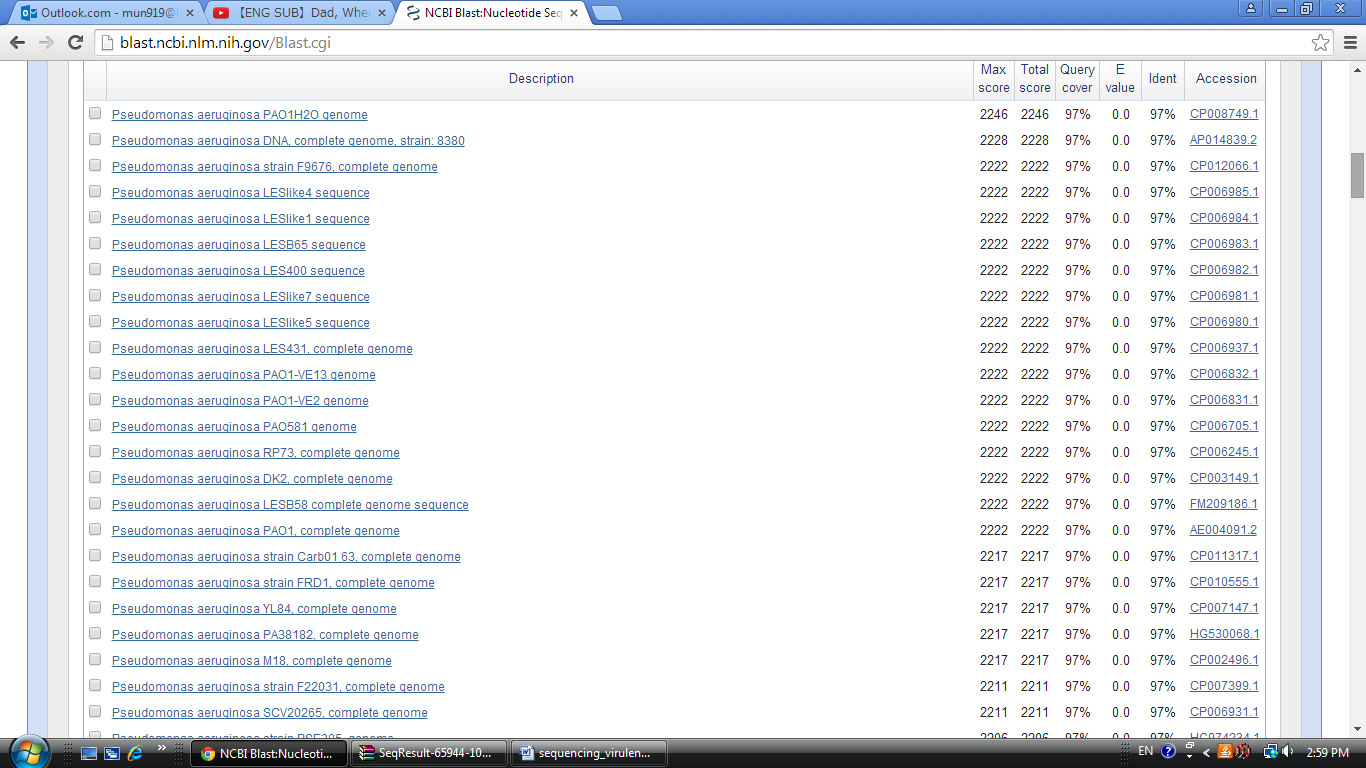


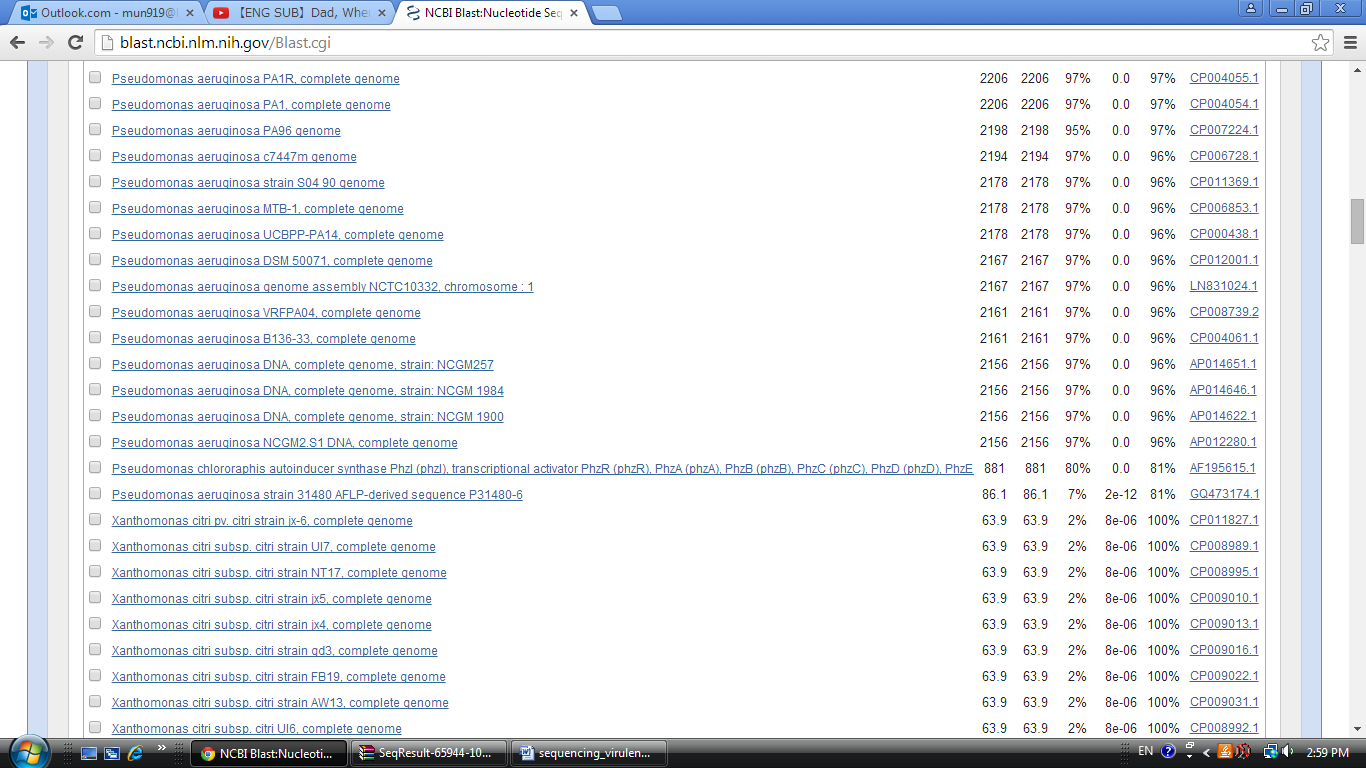


**Phenazine modifying enzyme (phzS)**

>1988157_phzS_phzS_F

TGGACGGAGCGAGGGCATCTGGAGCACCGCAACGCCCTGCTGGGTCACCGGCGGCTGGCGGTCATCGACCTCAGCGGCGGCGTGCAGCCGATGTCCTATCGCTTTCCCACCGGCCAGGAGGTCACCCTCGTCTACACCGGCGAGGTGTACAACCACGATGCCCTGCGCGAGCGGTTGCGCCGGGCCGGACATGAGTTCCGCACCCGCAGCGATACCGAGGTGGTCCTGCACGCCTATCTGCAATGGGGCGAGCGTTGTTGCGAGTACCTGACCGGGATGTTCGCCTTCGCCGTCTTCGATGGCCGCGACGGCCACCTGCTGCTGGTGCGCGACCGCCTGGGCATCAAGCCGCTGTATTACGCGCGGCACCGCGAGGGACTGCTGTTCGGCTCGGAGATCAAGTCCATCCTGGCGCATCCGGAATTCGCCGCCAGGCTCGACGCGGTCGGCCTGGTCGACCTCCTGACGCTGTCCCGGGGCACTTCGCAGACGCCGTTCCGCGAGGTCCAGGAACTGCTGCCCGGCCACCTGCTGTCCTGGCGTCCCAATTCCCAGGCGAAGTTGCGCCGCTATTGGGAGGTACGCCGCCAGGAGCATGCCGACGACCTGCAGAGCACCGTGCAGCGCACCCGCGAACTGGTCACCCGCGCCCTGGGGGCGCAATTGCACGCCGACGTTCCGGTGTGTTCGCTGCTATCGGGTGGGCTCGATTCGACCGCCCTGACCGGCATCGCCCAGCGCATCGCGAAGGCGGAGCACGGCGGCGACATCAATTC

GTTCTCGGTGGACTTCGTCGGCCAGGCCGAGCAGTTCCGCAGCGACGACCTGCGTCCCGACCAGGACCAGCCGTTCGCCCTGCTGGCCGCGCAGTACATCGGCAGCCGTCATCGCACCGTGCTCATCGACAATGCCGAACTGGTCTGCGAACGAGCGCGCGAAGAGGTATTCCGGGCCAAGGACGTACCTTCCACCTTCGGCGACATGGATACCTCGCTGCACCTGATGTTCCGGCGAGATCCGCCCGGCATTCCACGGTGGGCCATCTCCCGGTGAAAGGCGCCCAATGAAGCTGTTTGGGTGGGCTACCGGCTGGTTCCCGCGATCCCCAAGGCGGGGGGCTGCGGNCGCGCTTCCCCTGGGGCCTCCAGGGTGGCGTCTGGCCGGNCGGNTTTCATCGAACGCCGGGTTTCAACCGGCCGTTGCGGATNCCCTCCAGTACCCANANGGGCAGTTAACGACGAATGGGCTTGCCCCAGGTCAAAACACCTGGGCCCGGNAAACGTCCCGGAAGGAACGGCNGGAATGCNCGAATTTCAGCCCCCTTGAATCTGAAACCTCNTGAATGGGNCTCNCGNTCCCTAANCCANNGNATCCNNCTGGATATNTGTGCAAACGCCCNTGGANAGGTCGGTGGTCCCTNAACNCCCACACAAGGATCTGGGTGGANAAANCNCTCCAACATAGGNCCCCTGGGNCCAATAAAGAANCCTGNGTNACNGTGNTAAGATAAAATGTNNGTCGNNCATGNGNTGCTTNGCNNCCCATATNNTNTCNCNGGGAAGTGGNNGTTNNTAGNNNCTNCGGATNAGTCTTTTTACAGGTTTTTCGCGNACGCGGGNTGAATAATGCTATTGCTGTNAGAGTNAGTTGCCGGCC

>1988158_phzS_phzS_R

CGGTCTCAGTTGTGGCGGCTCACCTGGGTGTTGAAGTACCCCTCCGGATGCTCCAGTTCGGCGGCCAGGAACTCTCGCGAAACGATGCCGAACACCGGGTTCGCCGCGTCCTCCAGCAGGCGCCGCACGCTCCCGCGCAGGAAACGCTCGTAGCCGAGGTTGGCAGAAGTCGGATAAGGGCTCTTGCGGCGCTTGAGCACGGCTTCCGGGACATAGTCGGCGCAGGCCCGCTTGAGCAGCCACTTCTCCTCGCCGTCCCGGCTCTTGATCGACCAGGGCACGTTGTAGACGTACTCCACCAGCTCATGGTCGGTGTAGGGCACCCGCACCTCCAGGCCGTTGCACATGCTCAGGCGATCCTTGCGTTCGAGCAGCAGCACCATCCAGCGCTTCAGATGCAGGTGGCTGAACTCGCGCATCCGCCGCTCCTCCGGGCTGTCGCCGGCCAGGTGTTCGACCTGGCGCAGCCCATCGTCGTAGCTGGCCTGCTGGTACTGGAGGAGATCGCAGCGGCGGTTGAAACCGGCGTCGATGAAGCCGGCCGGCAGGCGCACCCTGGAGGCCCAGGGGAAGCGCGCCGCAGCCACCGCCTGCGGATCGCGGAACCAGCCGTAGCCACCGAACAGCTCATCGGCGCCTTCACCGGAGATGGCCACCGTGGAATGCCGGCGGATCTCGCCGAACATCAGGTGCAGCGAGGTATCCATGTCGCCGAAGGTGGAAGGTACGTCCTTGGCCCGGAATACCTCTTCGCGCGCTCGTTCGCAGACCAGTTCGGCATTGTCGATGAGCACGGTGCGATGACGGCTGCCGATGTACTGCGCGGCCAGCAGGGCGAACGGCTGGTCCTGGTCGGGACGCAGGTCGTCGCTGCGGAACTGCTCGGCCTGGCCGACGAAGTCCACCGAGAACGAATTGATGTCGCCGCCGTGCTCCGCCTTCGCGATGCGCTGGGCGATGCCGGTCAGGGCGGTCGAATCGAGCCCACCCGATAGCAGCGAACACACCGGAACGTCGGCGTGCAATTGCGCCCCCAGGGCGCGGGTGACCAGTTCGCGGGTGCGCTGCACGGTGCTCTGCAGGTCGTCGGCATGCTCCTGGGGGCGTACCTCCCAATAGCGGCGCAACTTCGCCTGGGAATTGGGACCCCAGGACAGCAGGTGGCCGGGCAGCAATTCCTGGACCTCCCGGAAACGGCTTCTGCAAATTGCCCCGGGACACGCTCCAGGAAGGTCAACCAGGCCTAACCGCCTTCAAGCCTGGNCGGCTAATTCCGGAAGGCCCCGGGATGGGACTTGTTCCCCGAGCGGAAAANGANNNCCTCCGGGNGGCCCCGCGGAAAAACAGGGGTTTGAGACCCAGGTNTGTCCGCACAACAAACATTGGGGCCTNCGGGCTCTCTTAATCGTGAAAGGGAAATTCCTTGGTGTGTNTCCTTAANNNCTCCTGCCCNTTGNNAAAATTGCTGCGGACACTCCAGGTNNNTCGTGGGTGTGGGAAATNATNNGTCGTGCCAGAANACCTCCTCCTGGGGATACGGTGGTCTACCTCCGGTGGTAAAAAAGTGTAGCCNGCCGTGNCGTGAAATAAACAAACCNCCNGCGCTCTNCG


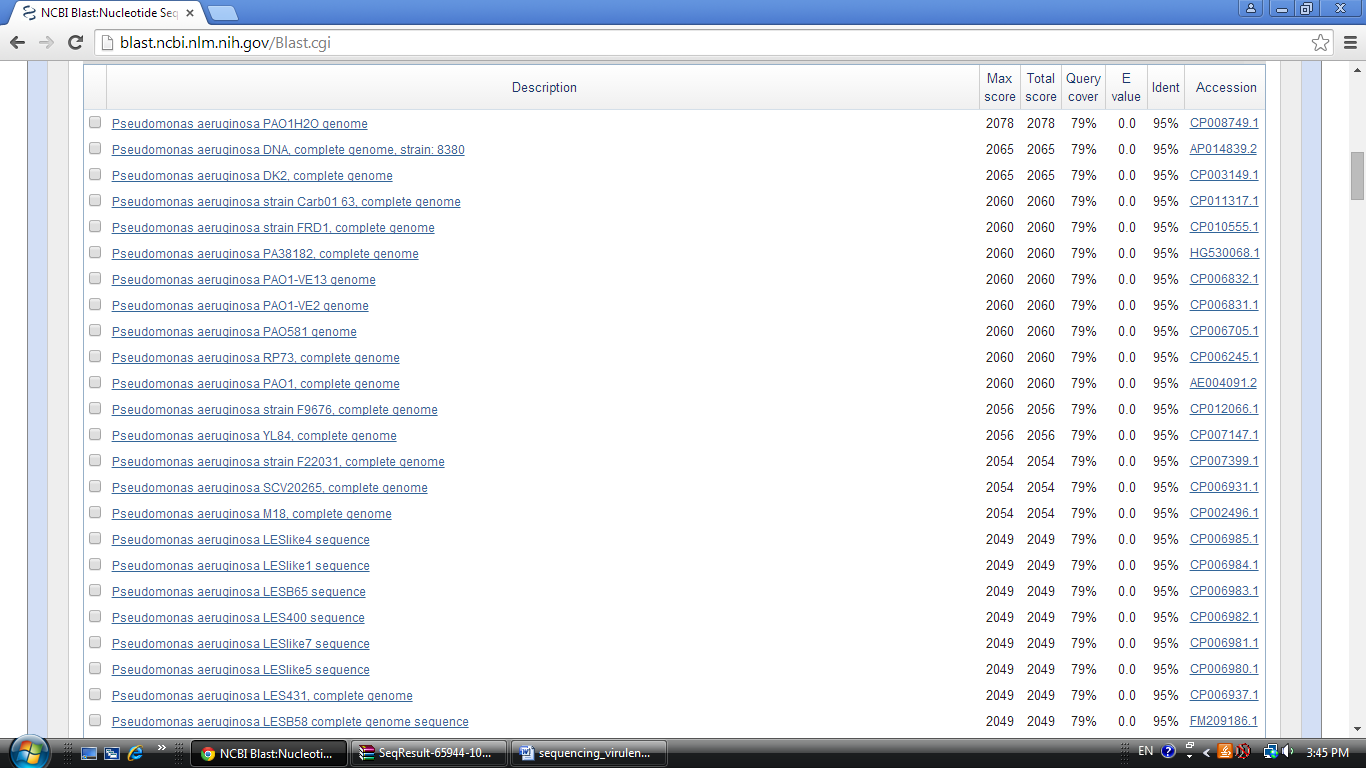

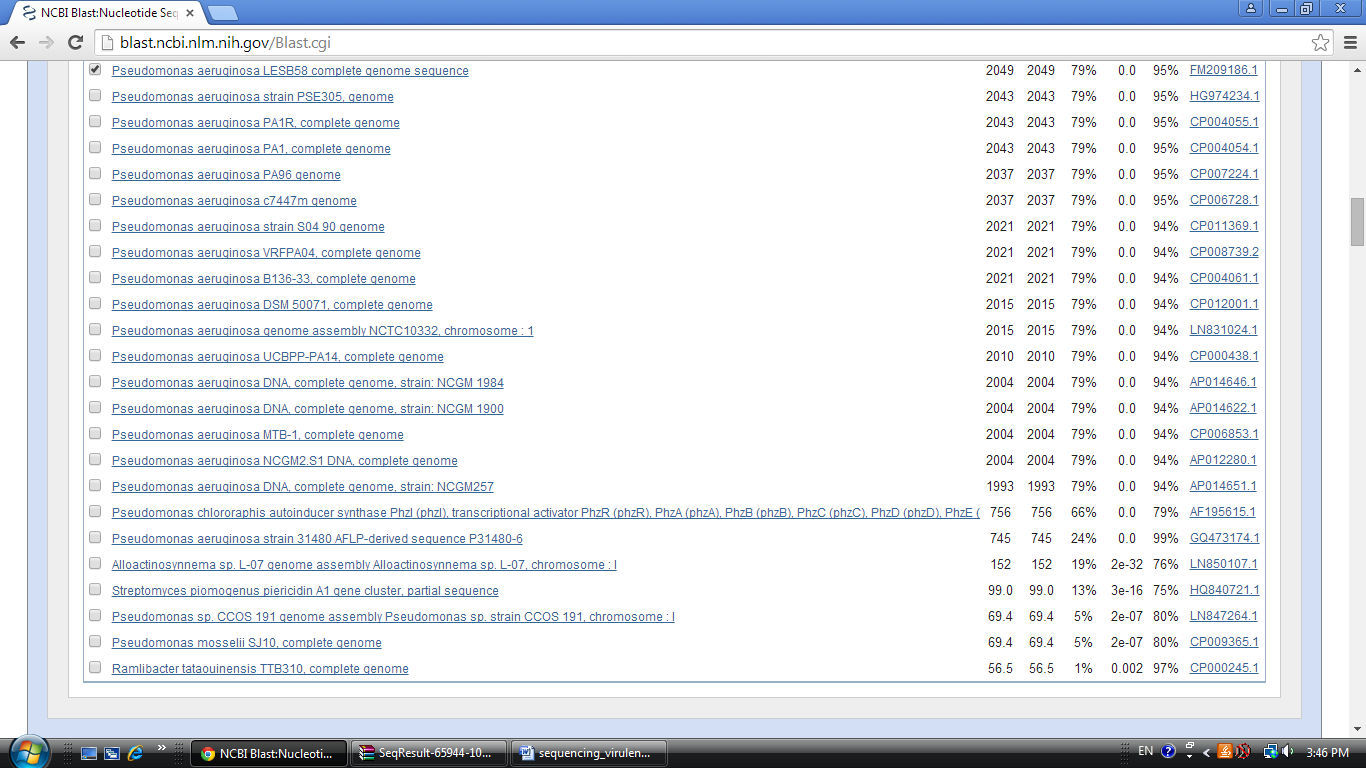


**Phenazine modifying enzyme (*phzM*)**

>1988165_phzM_phzM_F

TGCGCGCGGTCGGTTCGATGCCGAGCGCATCCATCGACTGATGCGCCTGCTGGTGGCCTTCGAGATCTTCCAGGGCGATACCCGCGACGGCTACGCCAATACCCCCACCAGCCACCTGCTGAGGGATGTCGAGGGCTCCTTCCGCGACATGGTGCTGTTCTACGGCGAGGAGTTCCACGCCGCCTGGACGCCCGCCTGCGAGGCGCTGCTCAGCGGTACCCCAGGCTTCGAGCTGGCGTTCGGCGAAGACTTCTACAGCTACCTGAAGCGCTGCCCGGATGCCGGCCGGCGCTTCCTGCTGGCGATGAAGGCGAGCAACCTGGCCTTCCACGAGATCCCCAGGCTCCTGGATTTCCGCGGGCGTAGCTTCGTCGACGTCGGTGGCGGTTCCGGCGAATTGACCAAGGCCATCCTGCAGGCCGAGCCCAGCGCCCGGGGCGTGATGCTCGACCGCGAGGGTTCCCTCGGCGTGGCCCGCGACAACCTCTCCAGCCTGTTGGCAGGGGAGCGCGTCAGCCTGGTGGGCGGCGACATGCTGCAAGAGGTGCCGTCCAACGGCGATATCTACCTGCTGTCGCGGATCATCGGCGATCTGGACGAAGCCGCCAGCCTGAGGTTGCTCGGCAATTGCCGCGAGGCGATGGCCGGCGACGGCCGGGTGGTGGTGATCGAGCGGACCATCTCGGCCAGCGAGCCGTCGCCGATGTCGGTGCTCTGGGACGTGCACCTGTTCATGGCCTGCGCTGGCCGTCACCGCACCACCGAGGAGGTGGTCGACCTGCTCGGGCGCGGCGGCTTCGCGGTGGAGCGGATCGTCGACCTGCCGATGAAAAACCCNCATA

>1988166_phzM_phzM_R

CGACNGCGCGAGCGCCGCGCCCGAGCAGGTCGACCACCTCCTCGGTGGTGCGGTGACGGCCAGCGCAGGCCATGAACAGGTGCACGTCCCAGAGCACCGACATCGGCGACGGCTCGCTGGCCGAGATGGTCCGCTCGATCACCACCACCCGGCCGTCGCCGGCCATCGCCTCGCGGCAATTGCCGAGCAACCTCAGGCTGGCGGCTTCGTCCAGATCGCCGATGATCCGCGACAGCAGGTAGATATCGCCGTTGGACGGCACCTCTTGCAGCATGTCGCCGCCCACCAGGCTGACGCGCTCCCCTGCCAACAGGCTGGAGAGGTTGTCGCGGGCCACGCCGAGGGAACCCTCGCGGTCGAGCATCACGCCCCGGGCGCTGGGCTCGGCCTGCAGGATGGCCTTGGTCAATTCGCCGGAACCGCCACCGACGTCGACGAAGCTACGCCCGCGGAAATCCAGGAGCCTGGGGATCTCGTGGAAGGCCAGGTTGCTCGCCTTCATCGCCAGCAGGAAGCGCCGGCCGGCATCCGGGCAGCGCTTCAGGTAGCTGTAGAAGTCTTCGCCGAACGCCAGCTCGAAGCCTGGGGTACCGCTGAGCAGCGCCTCGCAGGCGGGCGTCCAGGCGGCGTGGAACTCCTCGCCGTAGAACAGCACCATGTCGCGGAAGGAGCCCTCGACATCCCTCAGCAGGTGGCTGGTGGGGGTATTGGCGTAGCCGTCGCGGGTATCGCCCTGGAAGATCTCGAAGGCCACCAGCAGGCGCATCAGTCGATGGATGCGCTCGGCATCGGAACCGACCGCGGCGGCCAGCGTCTCGTCGCTGTCGACCGCGCTTCTCCATA


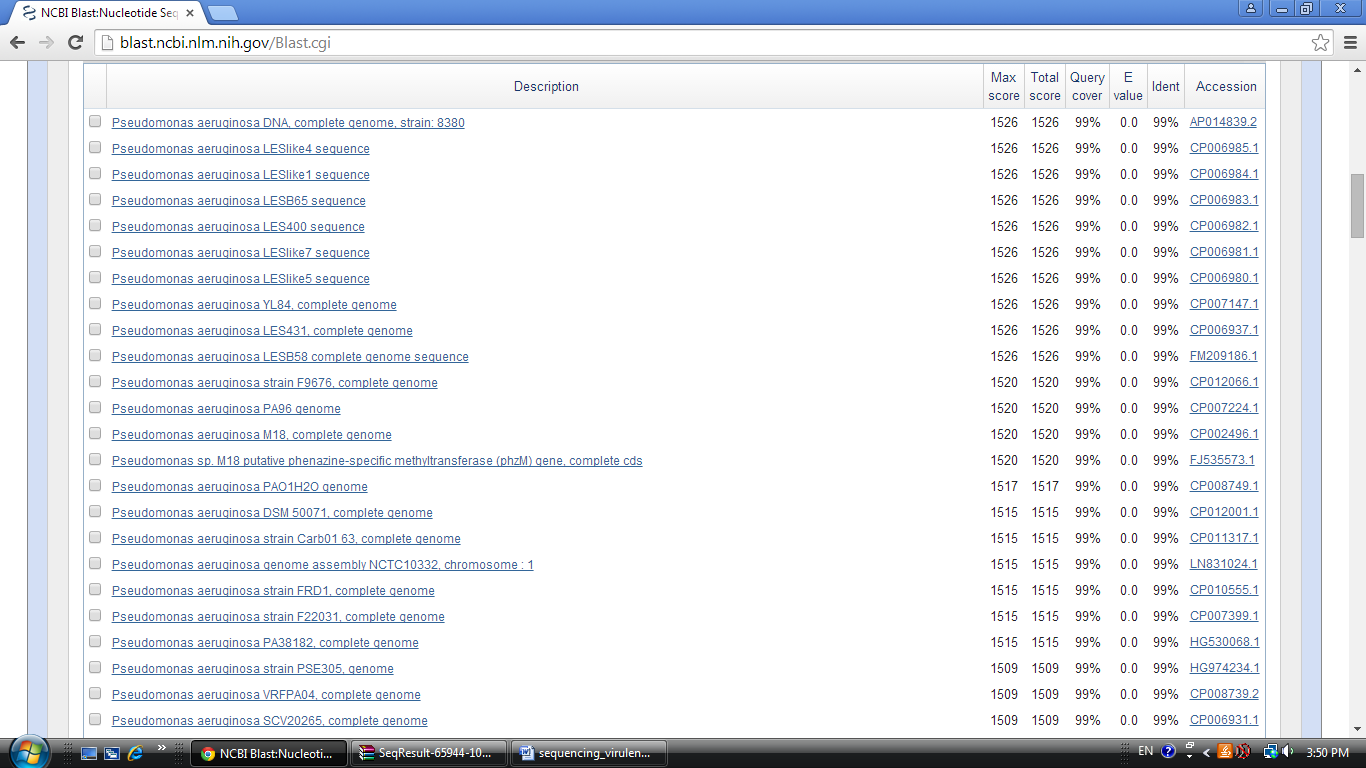


**T3SS effector (*exoS*)**

>1988159_exoS_exoS_F

GCATCTACGTTCCGGTTTGCTTGCCAGGTCGAGAGCATCGAGCAGTCCCTGGCTATGGCCACTCTGCTCCCCCAGGGCCGCCTCTTCGAGAACCCGGCGGGTCACTCCCTGCTCATCGCTGGCGCTCAGCAGCACGCGCATGTCGGTTTCTTTGTTATAGAGAATCTCTTTTTCATTCTTGTAGTTCGATATCCCGCTGACATCGATTCCGGACCTGCCGAACACGGTGGATATCGTGCCCTGCCCGAAGCTCCTCGCGACACCGGGGTTCAGGGAGGTGGAGAGATAGCCGTCGTA

>1988160_exoS_exoS_R

CGGGGATGCTTGCGAGTACTTCGGGCAGGGCACGATATCCACCGTGTTCGGCAGGTCCGGAATCGATGTCAGCGGGATATCGAACTACAAGAATGAAAAAGAGATTCTCTATAACAAAGAAACCGACATGCGCGTGCTGCTGAGCGCCAGCGATGAGCAGGGAGTGACCCGCCGGGTTCTCGAAGAGGCGGCCCTGGGGGAGCAGAGTGGCCATAGCCAGGGACTGCTCGATGCTCTCGACCTGGCAAGCAAACCGGAACGTTCAGGCGAGGTCCAGGAACAGGATGTACGCCTGAGGATA


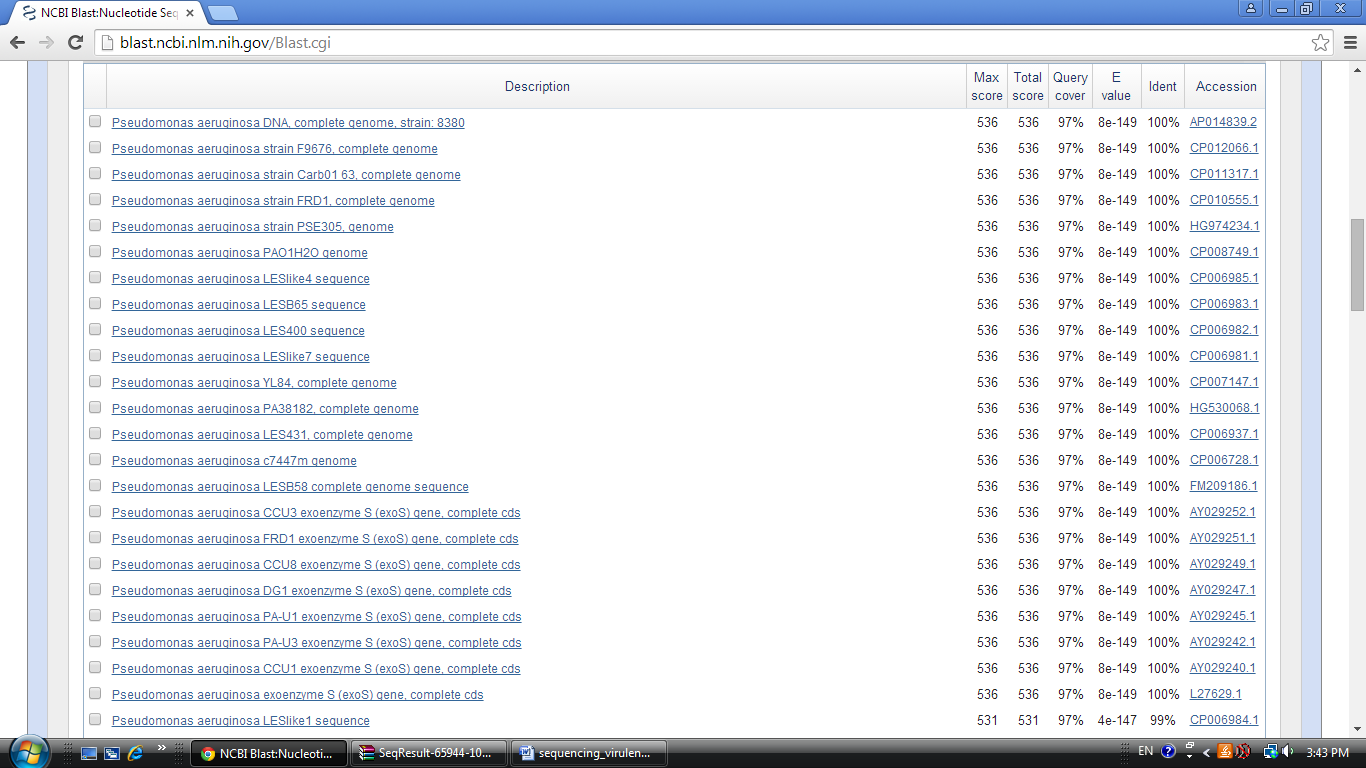


**T3SS effector (*exoU*)**

>1988161_exoU_exoU_F

CGATNNNAGCCTGTCGACCCCGTCGCAGGCAGCGCATAAGTCCGCCAGCTTGCGTCAGGAACCTTCAGGGCAAGGTCTCGGGGTTGCCCTAAAGAGCACGCCGGGAATACTTTCCGGGAAGTTGCCGGAAAGCGTTAGCGACGTGCGTTTCAGCAGTCCCCAAGGGCAAGGGGAGTCCCGTACTCTGACTGACTCGGCAGGGCCGCGGCAGATCACTCTGCGCCAGTTTGAGAACGGAGTCACCGAGCTACAGCTCAGTCGGCCACCATTGACCAGTCTGGTCCTAAGCGG

CGGTGGTGCCAAAGGTGCGGCATACCCGGGAGCAATGCTGGCGCTAGAAGAGAAAGGCATGCTCGATGGCATCCGCAGCATGTCCGGTTCGTCCGCTGGCGGCATCACCGCCGCCCTTTTGGCCTCAGGTATGAGCCCGGCGGCGTTCAAGACCCTTTCCGACAAGATGGATCTTATTTCGCTGCTCGACAGCTCGAACAAGAAGCTGAAGCTGTTCCAACACATTAGCAGCGAGATCGGCGCATCGCTGAAAAAGGGCTTGGGCAACAAGATCGGCGGCTTCTCTGAGTTGCTGCTCAATGTACTCCCACGCATAGATTCGCGGGCTGAGCCCCTAGAACGCCTATTGCGCGACGAGACACGCAAGGCCGTGCTCGGACAGATCGCTACGCATCCAGAGGNTTGCACGCCAGCCGACCGTTGCCGCCATCGCCAGCANATTGCATCCCGGCTCCGGAGTCACNTTTGGCGATCTAGATCGGNTGAGTGCTTACNTNTCCCAGATTAGNATGCTNAACATCACNAGTACT

>1990206_exoU_exoU_R

CGTGCAGCGGTTCAGTGCATCCACATCGTCAACGCCATCACGTGCCTCAGCCCAGAGCACAAGTCTACCGGCATCGTCCGCCGCAAGTTTCACCGGTGCCAAGGATGAGAATGGAGAGTTCACTTGTAACAATTGCAAGATCACACCCAGCGGTAACCCTGAAGGCACAAGTCCCAACTCGACGACGAAAAGCCAACCGCCCTCGAGGCGCTCCATAACCAGTTCCGGTCCCTCTGCCGGTTGCAGCCGCAACGTGGCATCGTTGCTCGAGGGAAGTCTCAAGCCCCACTGTGCCAGCCATGTATCAATCATGTGAACTCCTTATTCCGCCAAGCCTTAGCCATCTCAACGGTAGTCGAACTCAAGGGTTTGCCGAAACGCAGGAAGCCTCGTGCCGAGTAGTTGTCGACGATATCGTTCAGCGCTTGATTGATTTCCGCCGGACTGGTGGCATGCCGTAGCTGCTCCTCCGCCCGACGTAACAGAGCTACGTTGGAATCCGGCTGGCCAGGGCGATACAGAGAGGGGAAGATAACTTCCTTACGAATGTTCTCGGCAATAACCGCCACTTTGCGGCGCTGCGCCTCTGCTTACGCAGCGGCGACCACCGGCGACTGCACCGTCTGCTCGCGCATGGTATCGATTANCTGCTGATGATCAACGTCATCANCTGGTTCAACTCATCTGCCACCCTTGT


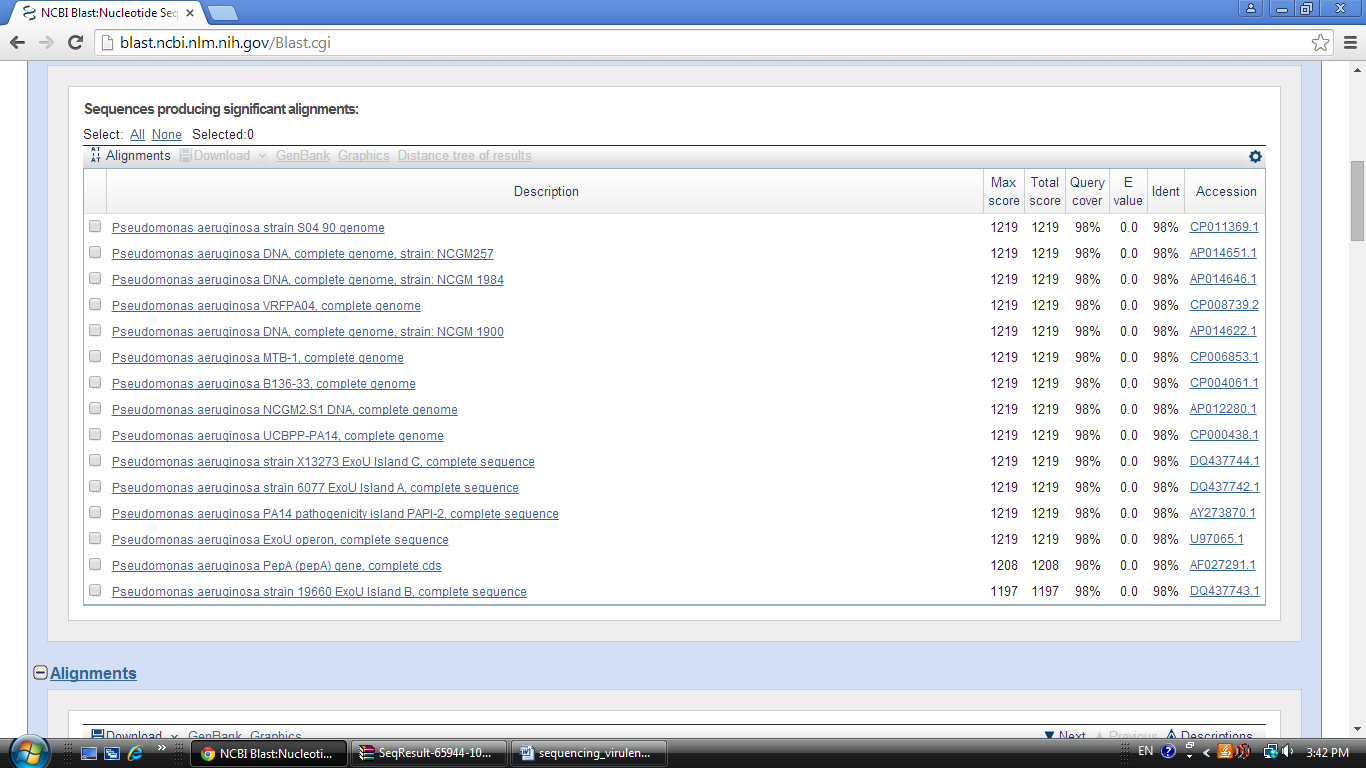


**T3SS effector (*exoY*)**

>1988163_exoY_exoY_F

CCACGGGCCGCCCGGACGCTACTGCGCCCTGCGGATATGCAGGCACGGGCTCTACAGGACCTGTTCGATGCGCAGGGGGTGGGGGTTCCCGTCGAACATGCCTTGCGCATGCAGGCGGTGGCCAGGCAGACGAATACCGTCTTTGGCATTCGCCCGGTTGAAAGAATCGTCACCACGCTGATCGAAGAGGGTTTCCCGACCAAGGGCTTCTCGGTGAAGGGGAAAAGCTCGAACTGGGGTCCGCAGGCGGGGTTCATCTGTGTCGACCAGCATCTCAGCAAGCGGGAAGACCGCGACACGGCGGAAATACGCAAGCTGAACCTGGCTGTGGCGAAGGGAATGGATGGCGGAGCCTATACCCAGACCGACCTGCGGATCTCTCGGCAACGCCTTGCGGAGCTGGTGCGGAATTTCGGCCTGGTGGCAGACGGTGTCGGGCCGGTTCGCCTCCTGACCGCGCAGGGCCCCAGCGGCAAACGCTATGAGTTCGAGGCCCGCCAGGAGGCGGACGGTCTCTACAGGATCAGCCGCCTGGGGCGGTCGGAAGCGGTGCAGGTACTGGCCAGCCCGGCTTGCGGGCTGGCGATGACCGCCGATTATGACCTCTTCCTGGTAGCGCCCTCGATCGAGGCGCATGGCAGTGGTGGTCTCGATGCAAGAAGGAATACCGCGGTCAGATACACCCCCCTCGGTGCAAAGGATCCCCTGAGCGAGGACGGATTCTATGGCAGGGAGGATATGGCCAGGGGAAACATCACTCCGCGCACGCGGCAACT

GGTGGACGCCCTCAATGACTGCCTGGGCCGGGGGGAGCACAGGGAGATGTTTCACCACAGCGACGATGCGGGCAACCCAGGCTCCCATATGGGTGACAACTTCCCGGCCACCTTCTACCTTCCCCGGGCCATGGAGCATCGGGTCGGAGAAGAGTCCGTTCGCTTCGACGAGGTCTGCGTGGTGGCGGATCGGAAGAGCTTTTCCTTGCTGGCGAGGGGGGCATCAAAA

>1988164_exoY_exoY_R

TCGATCGCACCACGCAGACCTCGTCGAAGCGAACGGACTCTTCTCCGACCCGATGCTCCATGGCCCGGGGAAGGTAGAAGGTGGCCGGGAAGTTGTCACCCATATGGGAGCCTGGGTTGCCCGCATCGTCGCTGTGGTGAAACATCTCCCTGTGCTCCCCCCGGCCCAGGCAGTCATTGAGGGCGTCCACCAGTTGCCGCGTGCGCGGAGTGATGTTTCCCCTGGCCATATCCTCCCTGCCATAGAATCCGTCCTCGCTCAGGGGATCCTTTGCACCGAGGGGGGTGTATCTGACCGCGGTATTCCTTCTTGCATCGAGACCACCACTGCCATGCGCCTCGATCGAGGGCGCTACCAGGAAGAGGTCATAATCGGCGGTCATCGCCAGCCCGCAAGCCGGGCTGGCCAGTACCTGCACCGCTTCCGACCGCCCCAGGCGGCTGATCCTGTAGAGACCGTCCGCCTCCTGGCGGGCCTCGAACTCATAGCGTTTGCCGCTGGGGCCCTGCGCGGTCAGGAGGCGAACCGGCCCGACACCGTCTGCCACCAGGCCGAAATTCCGCACCAGCTCCGCAAGGCGTTGCCGAGAGATCCGCAGGTCGGTCTGGGTATAGGCTCCGCCATCCATTCCCTTCGCCACAGCCAGGTTCAGCTTGCGTATTTCCGCCGTGTCGCGGTCTTCCCGCTTGCTGAGATGCTGGTCGACACAGATGAACCCCGCCTGCGGACCCCAGTTCGAGCTTTTCCCCTTCACCGAGAAGCCCTTGGTCGGGAAACCCTCTTCGATCAGCGTGGTGACGATTCTTTCAACCGGGCGAATGCCAAAGACGGTATTCGTCTGCCTGGCCACCGCCTGCATGCGCAAGGCATGTTCGACGGGAACCCCCACCCCCTGCGCATCGAACAGGTCCTGTAGAGCCCGTGCCTGCATATCCGCAGGGCGCAGTAGCGGTCCGGGCTGTGCCGTTGCGTTAGAAACCACCTGACAGAACCCCGTCCGATAAA


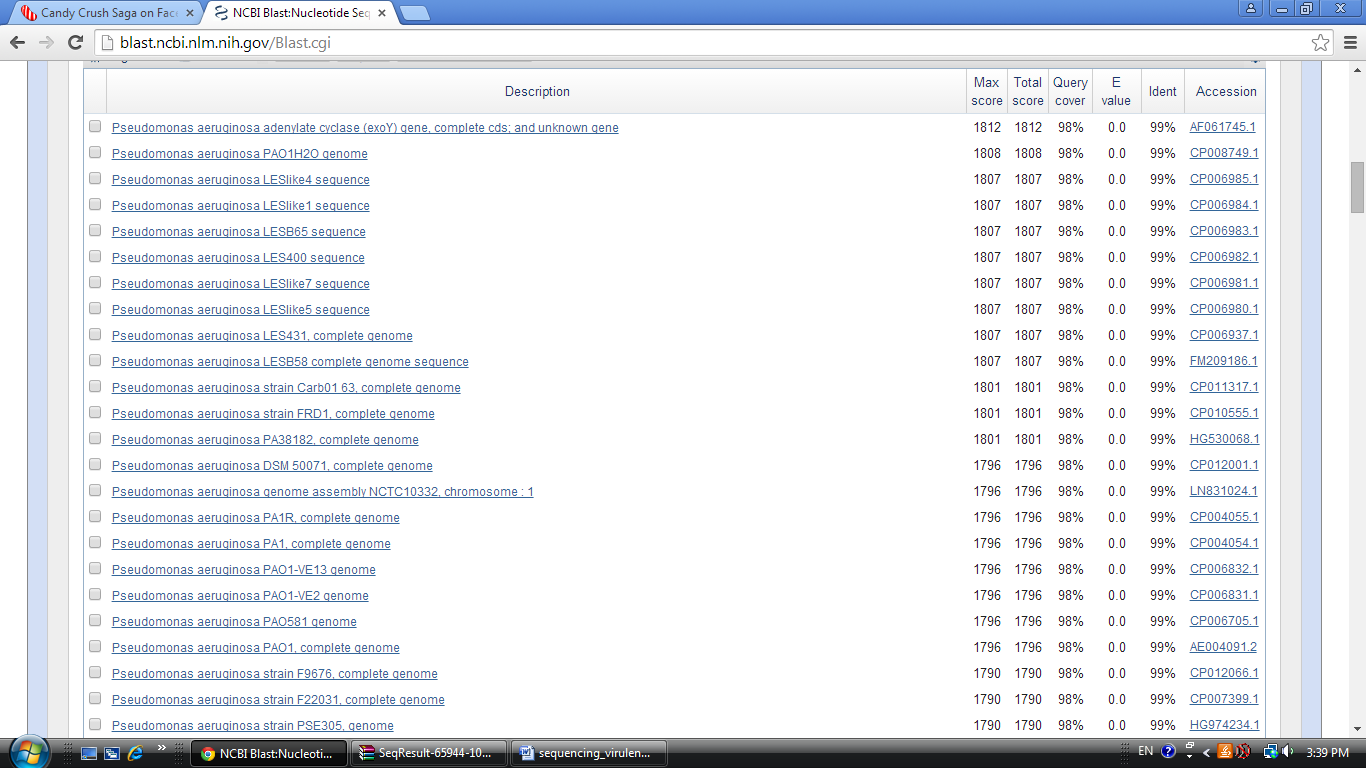


**T3SS effector (*exoT*)**

>3043613_exoT_PAO1_exoT_F

GGCCTGGCTTACTGAGCAGCCGTGGCCGGGCGCCTGGGACAGGTCGAGGCCCGCCAGGTGGCCACTCCCCGGGAGGCGCAACAACTGGCCCAGCGCCAGGAAGCACCGAAGGGCGAGGGCCTGCTCTCCCGCCTGGGGGCTGCCCTCGCGCGTCCCTTCGTGGCGATCATCGAGTGGCTGGGCAAACTGCTGGGGAGCCGTGCCCACGCCGCCACCCAGGCGCCGCTCTCCCGTCAGGACGCGCCGCCTGCCGCCAGTCTCTCTGCCGCCGAGATCAAGCAGATGATGCTGCAAAAGGCACTGCCCCTGACCTTGGGCGGACTTGGCAAGGCGAGCGAGCTGGCGACTTTGACAGCGGAGAGGCTGGCGAAGGATCACACGCGCCTGGCCAGCGGCGACGGCGCTCTGCGATCGCTGGCCACCGCCCTGGTCGGGATTCGCGATGGCAGCCGGATCGAGGCTTCCCGTACCCAGGCTGCCCGCCTGCTCGAACAGAGCGTTGGGGGGATCGCGCTGCAACAGTGGGGGACCGCGGGCGGTGCCGCCAGCCAGCATGTACTCAGCGCAAGCCCGGAGCAACTGCGCGAAATCGCCGTCCAACTGCATGCGGTAATGGACAAGGTCGCCCTGTTGCGCCACGCGGTAGAGAGCGAGGTAAAGGGCGAGCCTGTCGACAAGGCGCTGGCGGATGGCCTGGTGGAGCACTTCGGGCTGGAGGCGGAGCAGTACCTCGGCGAACACCCGGACGGGCCGTACAGCGATGCCGAGGTGATGGCGCTCGGTCTCTATACCAACGGCGAGTACCAGCACCTGAATCGGTCCCTGCGTCAGGGACGGGAGCTGGATGCGGGCCAGGCGTTGATCGACCGGGGCATGTCTGCCGCGTTCGAAAAGAGCGGACCGGCTGAACAGGTCGTGAAGACCTTCCGCGGCACCCAGGGCAGGGATGCCTTCGAGGCGGTGAAAGAGGGCCAGGTCGGCCACGACGCCGGCTATCTCTCCACCTCCCGGGACCCCGGCGTTGCCAGGAGCTTCGCGGGCCAGGGCACGATAACCACCCTGTTCGGCAGATCCGGGATCGATGTCAGCGAGATATCGATCGAGGGCGATAACAGAAAATTCCCTTTACACAAAAAAGTTTT

>3043614_exoT_PAO1_exoT_R

AGGGTCTTGATGGATTCTCGGCTGACTCGATCCCGGATCTGCCGAACAGGGTGGTTATCGTGCCCTGGCCCGCGAAGCTCCTGGCAACGCCGGGGTCCCGGGAGGTGGAGAGATAGCCGGCGTCGTGGCCGACCTGGCCCTCTTTCACCGCCTCGAAGGCATCCCTGCCCTGGGTGCCGCGGAAGGTCTTCACGACCTGTTCAGCCGGTCCGCTCTTTTCGAACGCGGCAGACATGCCCCGGTCGATCAACGCCTGGCCCGCATCCAGCTCCCGTCCCTGACGCAGGGACCGATTCAGGTGCTGGTACTCGCCGTTGGTATAGAGACCGAGCGCCATCACCTCGGCATCGCTGTACGGCCCGTCCGGGTGTTCGCCGAGGTACTGCTCCGCCTCCAGCCCGAAGTGCTCCACCAGGCCATCCGCCAGCGCCTTGTCGACAGGCTCGCCCTTTACCTCGCTCTCTACCGCGTGGCGCAACAGGGCGACCTTGTCCATTACCGCATGCAGTTGGACGGCGATTTCGCGCAGTTGCTCCGGGCTTGCGCTGAGTACATGCTGGCTGGCGGCACCGCCCGCGGTCCCCCACTGTTGCAGCGCGATCCCCCCAACGCTCTGTTCGAGCAGGCGGGCAGCCTGGGTACGGGAAGCCTCGATCCGGCTGCCATCGCGAATCCCGACCAGGGCGGTGGCCAGCGATCGCAGAGCGCCGTCGCCGCTGGCCAGGCGCGTGTGATCCTTCGCCAGCCTCTCCGCTGTCAAAGTCGCCAGCTCGCTCGCCTTGCCAAGTCCGCCCAAGGTCAGGGGCAGTGCCTTTTGCAGCATCATCTGCTTGATCTCGGCGGCAGAGAGACTGGCGGCAGGCGGCGCGTCCTGACGGGAGAGCGGCGCCTGGGTGGCGGCGTGGGCACGGCTCCCCAGCAGTTTGCCCAGCCACTCGATGATCGCCACGAAGGGACGCGCGAGGGCAGCCCCCAGGCGGGGAGAGCAGGCCCTCGCCCTTCGGTGCTTCCTGGCGCTGGGCCAGTTGTTGCGCCTCCCGGGGGAGTGGCCACCTGGCGGGCCTCGACCTGTCCCAGGCGCCCGGCCACGGCCTGGCTCAACTCACCACAAAGACGGTCGCTCTTAAAAAAAAAATGTGGAGCT


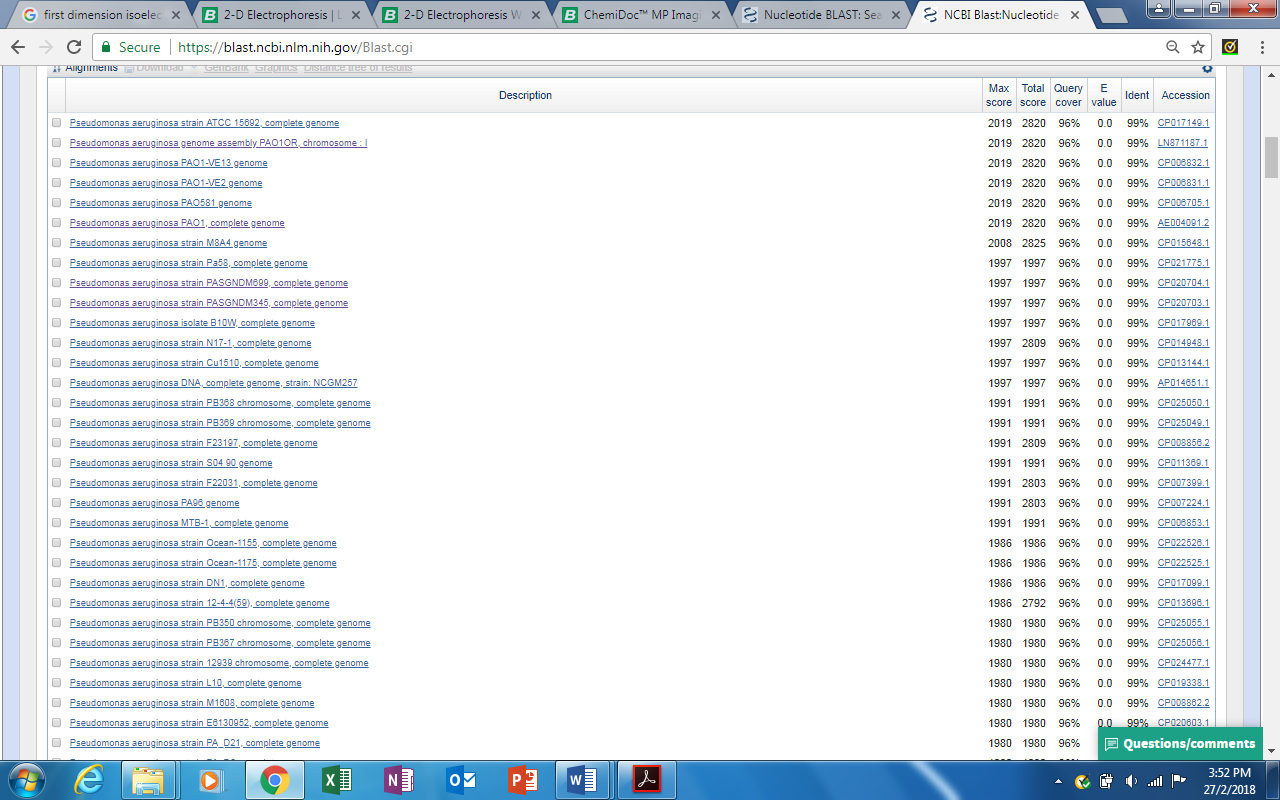


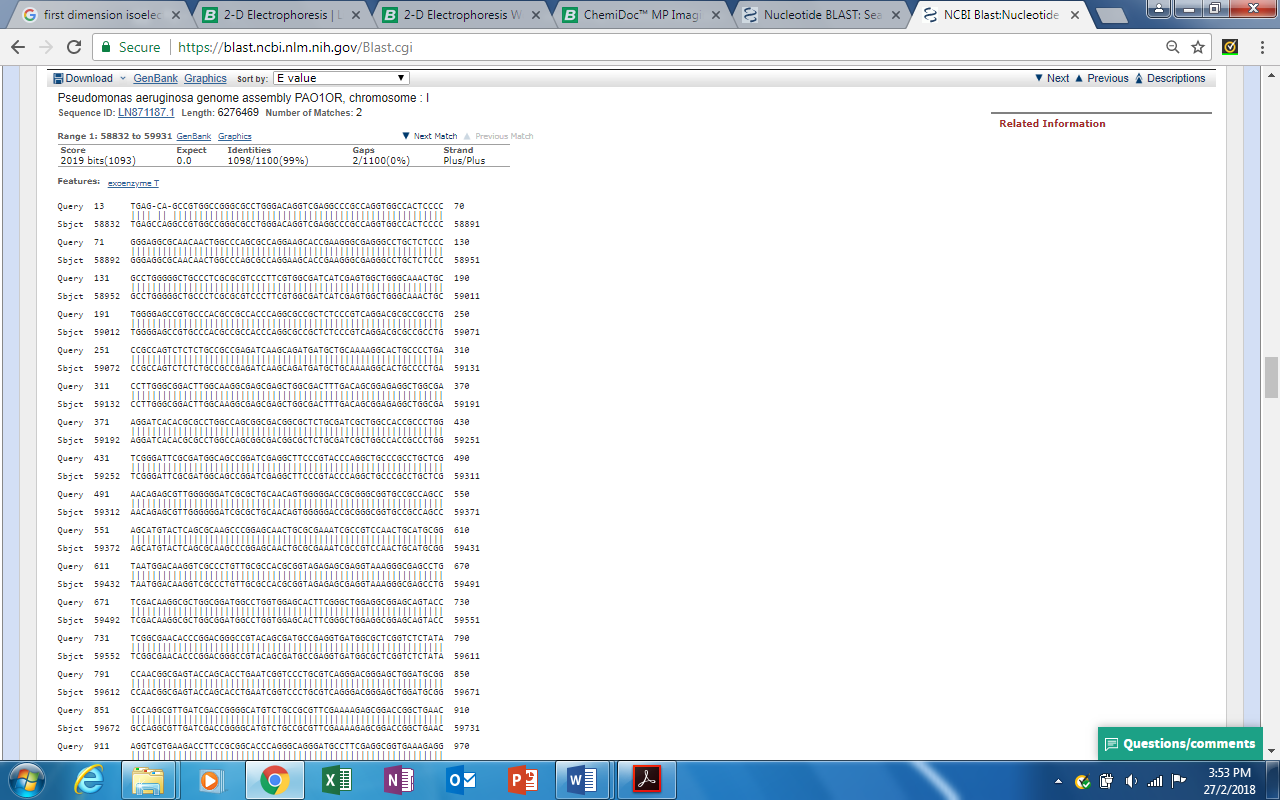


**Pyoverdine (*pvdA*)**

>1988167_pvdA_pvdA_F

ACTATCTATCGGTGTCGGCTTCGGCCCTTCCATATCGCCCTGGCGATTGCCCTCCAGGAACGGGCCCAGGCGCAGGGCGCCCTGGAAGTGCTGTTCCTGGACAAGCAGGGCGACTACCGCTGGCACGGCAACACCCTGGTGTCGCAGAGCGAGTTGCAGATCTCCTTCCTCAAGGACCTGGTGTCCCTGCGCAACCCCACCAGCCCGTATTCCTTCGTCAACTACCTGCACAAGCACGATCGCCTGGTCGACTTCATCAACCTGGGCACCTTCTATCCCTGCCGGATGGAGTTCAACGACTACCTGCGCTGGGTCGCCAGCCACTTCCAGGAGCAGAGCCGCTACGGCGAAGAGGTCCTGCGCATCGAGCCGATGCTGAGCGCCGGCCAGGTCGAGGCGCTGCGGGTGATCTCGCGCAACGCCGACGGCGAGGAACTGGTGCGCACCACCCGCGCCCTGGTGGTCAGTCCCGGCGGCACCCCGCGTATCCCGCAGGTGTTCCGTGCGCTCAAGGGCGACGGCCGGGTGTTCCACCACAGCCAGTACCTGGAGCACATGGCCAAGCAGCCCTGCAGCAGCGGCAAGCCGATGAAGATCGCCATCATCGGCGGCGGGCAGAGCGCGGCGGAGGCCTTCATCGACCTCAACGACAGCTACCCGTCGGTGCAGGCCGACATGATCCTGCGTGCCTCGGCGCTCAAGCCGGCGGACGATAGCCCGTTCGTCAACGAAGTGTTCGCGCCGAAGTTCACCGATCTCATCTACAGCCGCGAGCATGCCGAACGCGAGCGTTTGCTGCGCGAATACCACAACACCAACTATTCGGTGGTGGATACCGACCTGATCGAGCGCATCTACGGCGTCTTCTACCGCCAGAAAGTCTCCGGCATCCCGCGCCACGCCTTCCGTTGCATGACTACTGTGGAGCGCGCGACCGCCACCGCCCAGGGCATCGAGCTGGCGTTGCGCGACGCCGGTAGCGGCGAGCTAAGCGTAGAGATCTACGACGCAGTGATCCTGGCCACCGGCTATGAGCGCCAGTTGCACCGCCAACTGCTCGAACCGCTGGCGGAGTACCTCGGCGACCATGAGATCGGCCGCGACTACCGCCTGCAGACCGACAAGCGCTGCAAGGTGGCGATCTACGCGCAGGGTTTCAGCCAGGCCAGCCATGGCCTCAAGGAACCCCTGCTGTCGGGGCTGCCGGGACGGGCCAAGGAAAATCCCGGTCTCGTTCNGCCTACCGCCTGAAAATGGGANTTATTTTTGTGTTCTGCTACTCTTCTGCTCTTCTTTCTTCCTATCTGTCTACTATCTTATTCTACTATTGAGTTCCGATTCTAATTCTGTCTTAGTTACAGCTAGATTCATTACCGTCCTG

>1988168_pvdA_pvdA_R

GGGTCTGGGAGCACGGCAGCACCGACAGCAGGGTGTCGCTGAGGCCATGGCTGGCCTGGCTGAAGCCCTGCGCGTAGATCGCCACCTTGCAGCGCTCGTCGGTCTGCAGGCGGTAGTCGCGGCCGATCTCATGGTCGCCGAGGTACTCCGCCAGCGGTTCGAGCAGTTGGCGGTGCAACTGGCGCTCATAGCCGGTGGCCAGGATCACTGCGTCGTAGATCTCTACGCTTAGCTCGCCGCTACCGGCGTCGCGCAACGCCAGCTCGATGCCCTGGGCGGTGGCGGTCGCGCGCTCCACAGTAGTCATGCAACGGAAGGCGTGGCGCGGGATGCCGGAGACTTTCTGGCGGTAGAAGACGCCGTAGATGCGCTCGATCAGGTCGGTATCCACCACCGAATAGTTGGTGTTGTGGTATTCGCGCAGCAAACGCTCGCGTTCGGCATGCTCGCGGCTGTAGATGAGATCGGTGAACTTCGGCGCGAACACTTCGTTGACGAACGGGCTATCGTCCGCCGGCTTGAGCGCCGAGGCACGCAGGATCATGTCGGCCTGCACCGACGGGTAGCTGTCGTTGAGGTCGATGAAGGCCTCCGCCGCGCTCTGCCCGCCGCCGATGATGGCGATCTTCATCGGCTTGCCGCTGCTGCAGGGCTGCTTGGCCATGTGCTCCAGGTACTGGCTGTGGTGGAACACCCGGCCGTCGCCCTTGAGCGCACGGAACACCTGCGGGATACGCGGGGTGCCGCCGGGACTGACCACCAGGGCGCGGGTGGTG

CGCACCAGTTCCTCGCCGTCGGCGTTGCGCGAGATCACCCGCAGCGCCTCGACCTGGCCGGCGCTCAGCATCGGCTCGATGCGCAGGACCTCTTCGCCGTAGCGGCTCTGCTCCTGGAAGTGGCTGGCGACCCAGCGCAGGTAGTCGTTGAACTCCATCCGGCAGGGATAGAAGGTGCCCAGGTTGATGAAGTCGACCAGGCGATCGTGCTTGTGCAGGTAGTTGACGAAGGAATACGGGCTGGTGGGGTTGCGCAGGGACACCAGGTCCTTGAGGAAGGAGATCTGCAACTCGCTCTGCGACACCAGGGTGTTGCCGTGCCATCGGTAGTCGCCCTGCTTGTCCAGGAACAGAACTTCCAGGGTGCCCTGGTCCTGGACCCTTTCCTGGAGGGAAATCCCCAGGGCCAATTTGTGAGGGCCTAATCCTAACCCTTTTAATTCGGGAACTCGTGGGTGTATTCTTGCCTGAATCAATTTTTTTTAATGCGAAC


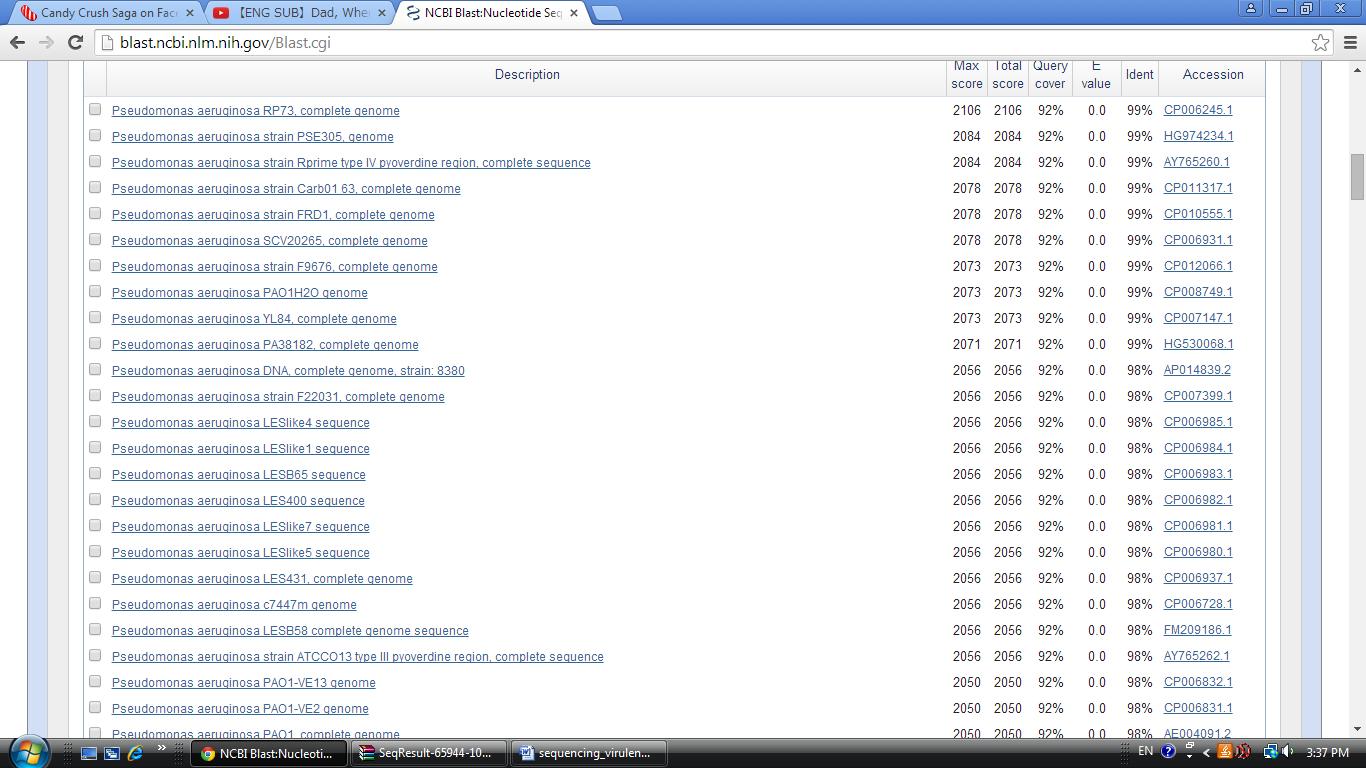


**Lectin (*lecA*)**

>1988169_lecA_lecA_u

CANGCACGAGCAGGCTTGCTGAAGGTAGGATCGCGAATCAGGGTTTTTCGCCTCTTTCGTTTATGAACAGGAATTCATATATCGGAGATCAATCATGGCTTGGAAAGGTGAGGTTCTGGCTAATAACGAAGCAGGGCAGGTAACGTCGATTATCTACAATCCGGGCGATGTCATTACCATCGTCGCCGCCGGTTGGGCCAGTTACGGACCTACCCAGAAATGGGGGCCGCAGGGCGATCGGGAGCATCCGGACCAAGGGCTGATCTGCCACGATGCGTTTTGTGGTGCGCTGGTCATGAAGATCGGCAACAGCGGAACCATTCCGGTCAATACCGGGTTGTTCCGTTGGGTTGCACCCAATAATGTCCAGGGTGCAATCACTCTTATCTACAACGACGTGCCCGGAACCTATGGCAATAACTCCGGCTCGTTCAGTGTCAATATTGGAAAGGATCAGTCCTGATAACTTGTCTCGGAAAAAAAGGGCCCGAATGGGCTCTTTTTTTAAATACAAATAAAGTGAAGTTGCCCGTGTGGCCGTTATGAACGGACAGGCAGCGCTTCGCAGTTGCGACTACCAATGACAAGGGTATCGAACTCCTGGGGCTGCCGCGGTCGTGGGAAACCGAGCGAGGGGGGGCGGGGAACTGCTTCAACACGCTTCGGTCTGAACGGGAATATCGATTCCTGACCCA

>1988170_lecA_lecAd

TTGGATTGCGTGTTGAGCAGTTCCCGCCCCCCCTCGCTCGGTTTCCCACGACCGCGGCAGCCCCAGGAGTTCGATACCCTTGTCATTGGTAGTCGCAACTGCGAAGCGCTGCCTGTCCGTTCATAACGGCCACACGGGCAACTTCACTTTATTTGTATTTAAAAAAAGAGCCCATTCGGGCCCTTTTTTTCCGAGACAAGTTATCAGGACTGATCCTTTCCAATATTGACACTGAACGAGCCGGAGTTATTGCCATAGGTTCCGGGCACGTCGTTGTAGATAAGAGTGATTGCACCCTGGACATTATTGGGTGCAACCCAACGGAACAACCCGGTATTGACCGGAATGGTTCCGCTGTTGCCGATCTTCATGACCAGCGCACCACAAAACGCATCGTGGCAGATCAGCCCTTGGTCCGGATGCTCCCGATCGCCCTGCGGCCCCCATTTCTGGGTAGGTCCGTAACTGGCCCAACCGGCGGCGACGATGGTAATGACATCGCCCGGATTGTAGATAATCGACGTTACCTGCCCTGCTTCGTTATTAGCCAGAACCTCACCTTTCCAAGCCATGATTGATCTCCGATATATGAATTCCTGTTCATAAACGAAAGAGGCGAAAAACCCTGATTCGCGATCCTTCCCTGTCAGCAAAGCACAGCAACGAGGAAGTACCGCCTACCAATTCATTGCAGGAGA


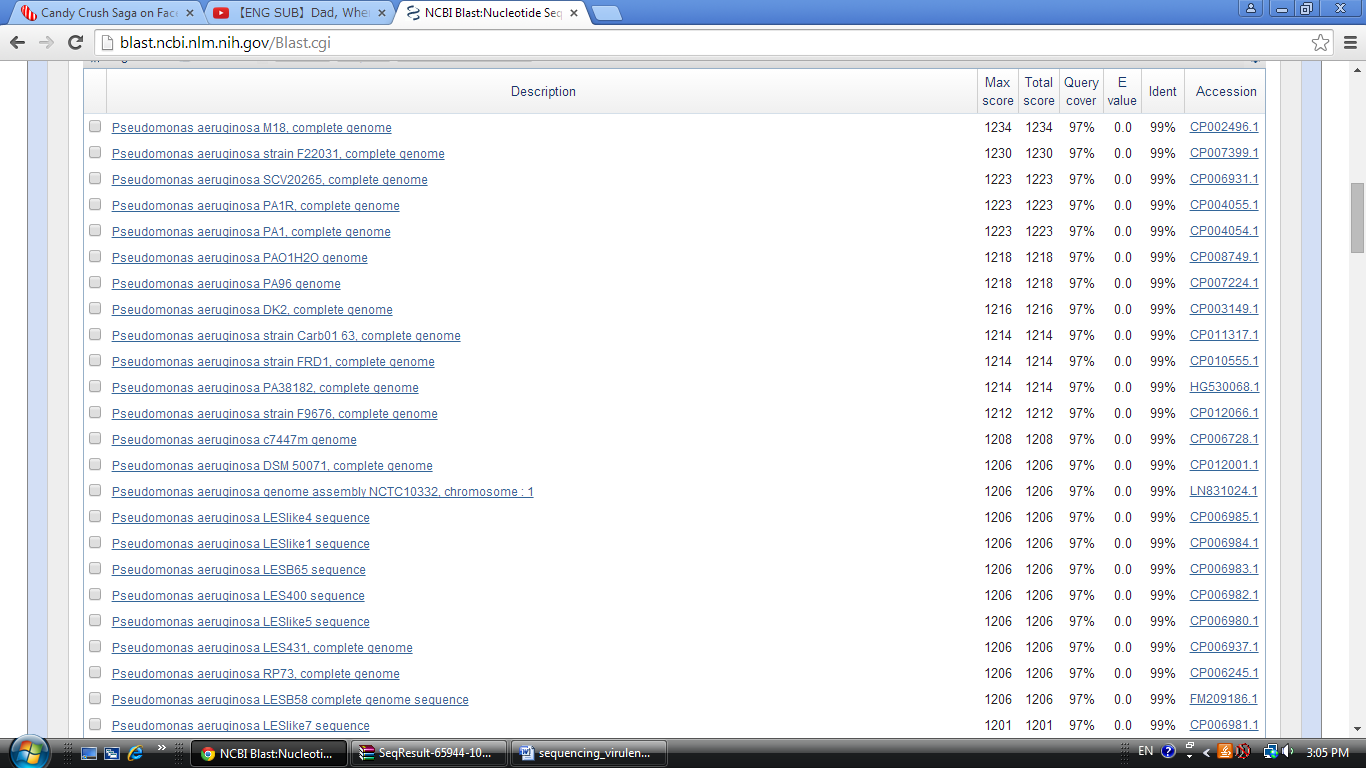


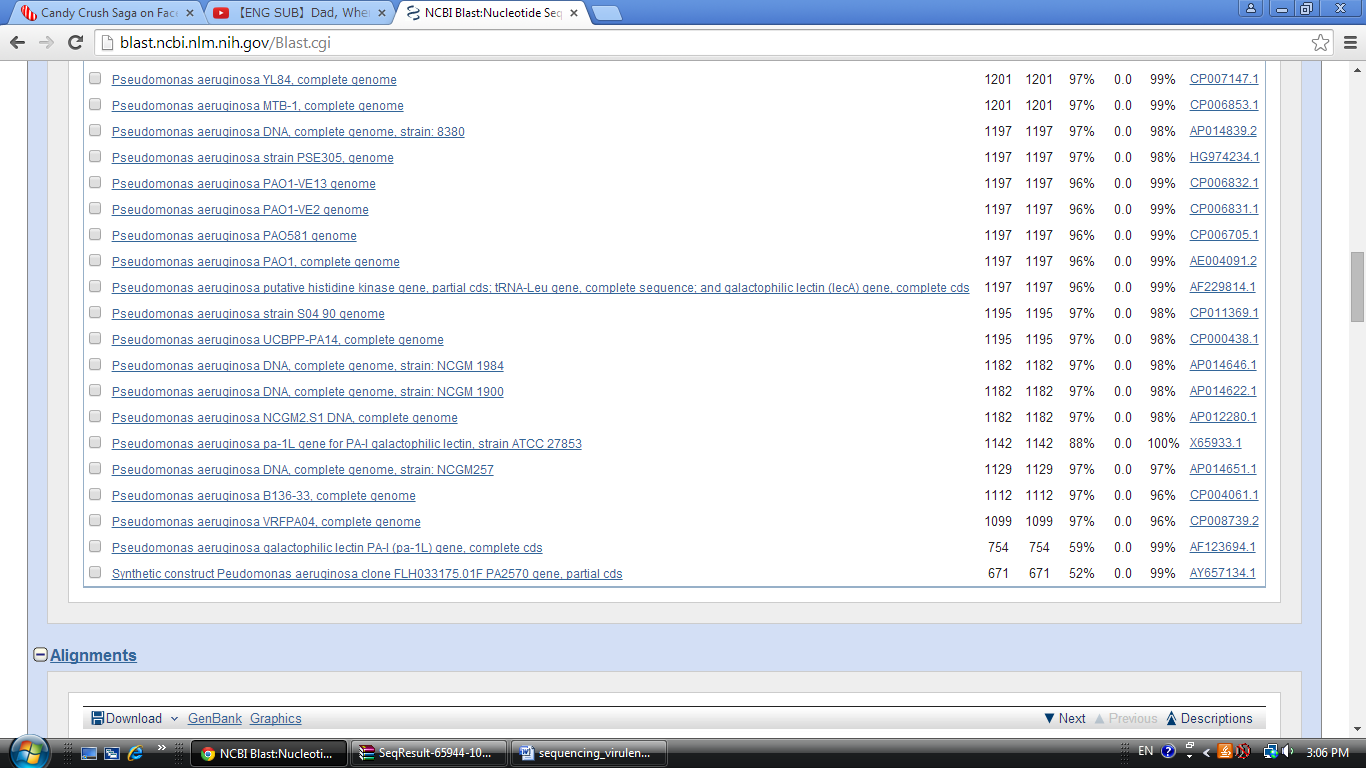


**Lectin (*lecB*)**

>1988171_lecB_lecB_u

ATAGCTAGCTGCGGTCTGCACGGGCATGCATCGCCAGTGCCAGGACACCCCTGTAGCCCTCCTGCGAACTCTAGCAGTGGTCGCGCCCGGTCGACGGCGTAGATTGGCTGAACGATGCGTCCTTCCTGCCTGCGCATCGCCTGCCGCCAAGGACTGCGGATTTCTCCCGACACGCCATACCGCGCGCCAGACGGCTGCCTGAAAAGGCAGGCCAGGTATTCAGTGGAGATACACCATGGCAACACAAGGAGTGTTCACCCTTCCCGCCAACACCCGGTTCGGCGTCACCGCCTTCGCCAACTCGTCCGGAACCCAGACGGTGAACGTGCTGGTCAACAACGAGACGGCCGCGACCTTCAGCGGGCAAAGCACCAATAACGCCGTCATCGGCACCCAGGTGCTCAACTCCGGCAGCAGTGGCAAGGTACAGGTCCAGGTCAGCGTCAACGGCCGCCCCTCGGATCTGGTCTCGGCACAGGTAATCCTGACCAACGAGCTGAACTTCGCCCTGGTCGGCTCTGAAGACGGCACCGACAACGACTACAACGACGCCGTCGTGGTGATCAACTGGCCGCTCGGCTAGGAGTTCGGAAGGGACGGGA

>1988172_lecB_lecB_d

CGAAGCGATTCTCTGCTGCTGGGGCTGTCCGCAGCATCCAGAAGCCGGTGGCCTACCTGACCCTGGCCAGCGCCATATTCACCGCCTGGTTGCCCGGCCTGCTGCTGCTCGGGCAGGTGCTCAAGGCATAGCAGGAAGTCGGAAAGGGATGGCGGCTTGCCGCCATCCCGTCCCTTCCGAACTCCTAGCCGAGCGGCCAGTTGATCACCACGACGGCGTCGTTGTAGTCGTTGTCGGTGCCGTCTTCAGAGCCGACCAGGGCGAAGTTCAGCTCGTTGGTCAGGATTACCTGTGCCGAGACCAGATCCGAGGGGCGGCCGTTGACGCTGACCTGGACCTGTACCTTGCCACTGCTGCCGGAGTTGAGCACCTGGGTGCCGATGACGGCGTTATTGGTGCTTTGCCCGCTGAAGGTCGCGGCCGTCTCGTTGTTGACCAGCACGTTCACCGTCTGGGTTCCGGACGAGTTGGCGAAGGCGGTGACGCCGAACCGGGTGTTGGCGGGAAGGGTGAACACTCCTTGTGTTGCCATGGTGTATCTCCACTGAATACCTGGCCTGCCTTTTCAGGCAGCCGTCTGGCGCGCGGTATGGCGTGTCGGGAGAAATCCGCAGTCCTTGGCGGCAGGCGATGCGCAGGCAGGAAGGACGCATCGTTCAGCCAATCTACGCCGTCGACCGGGCGCGACCACTGCTAGAGTTCGCAGGAGGGCTACAGGGGTGTCCTGGCACTGGCGATGCATGGCCCGGTGCCGGACTGCGGCAGCGGCCTGCCGTGCCGGCTCGTCCGATTGTTAAA


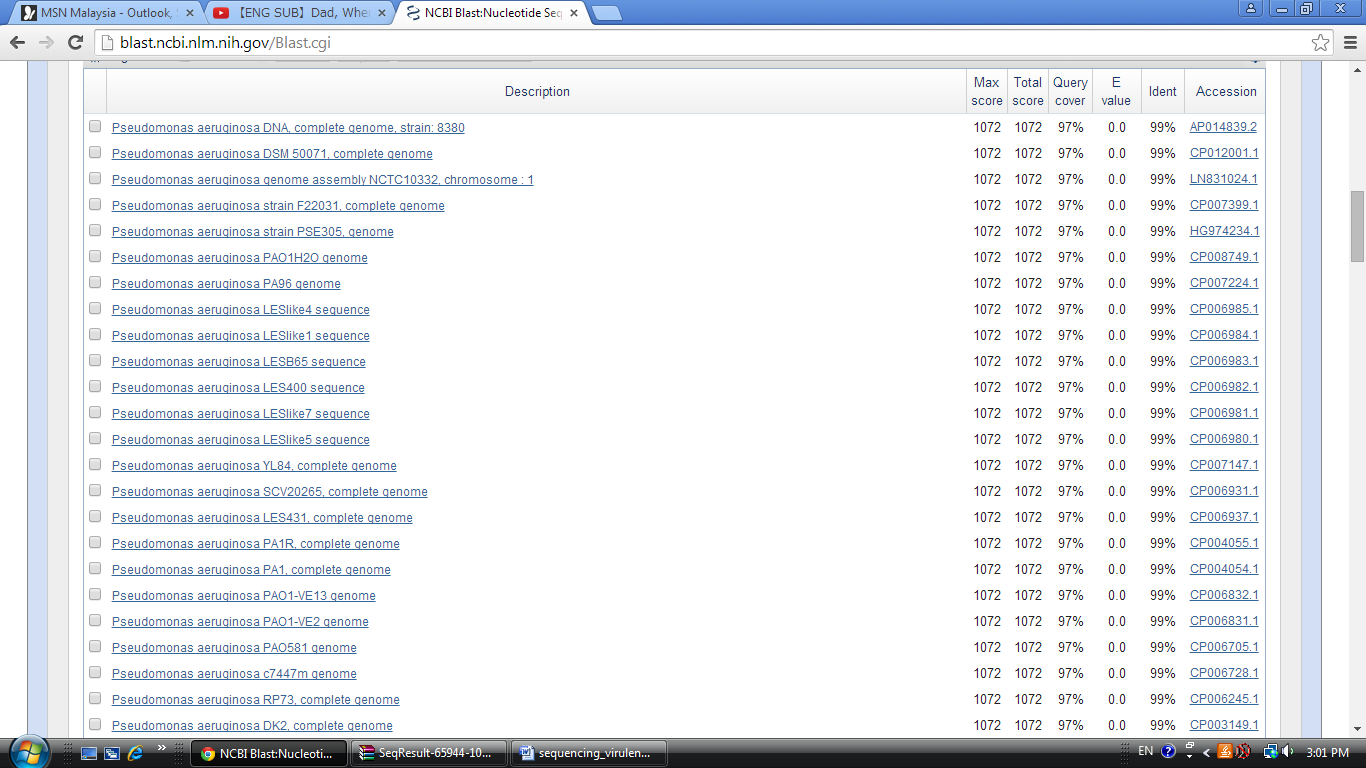


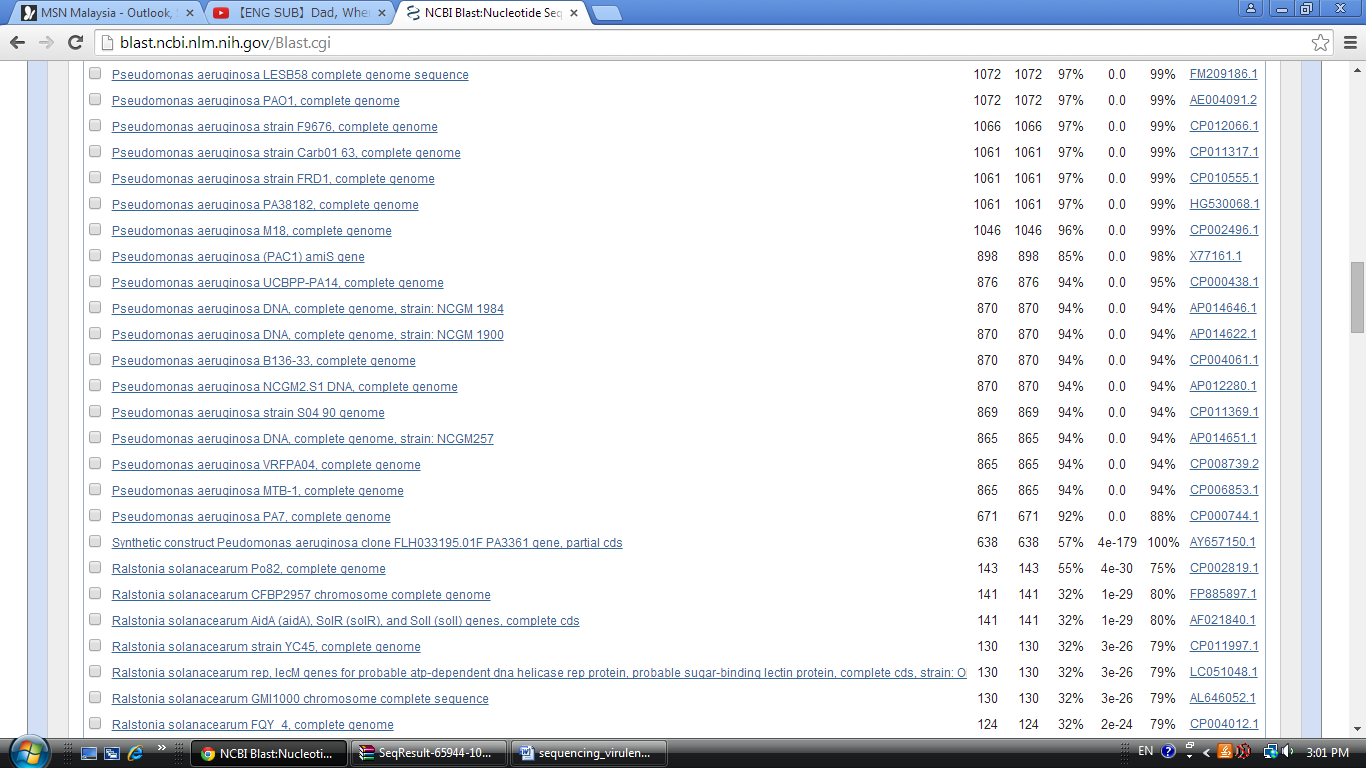


**Pili (*pilB*)**

>1980322_pilB_x5_pilB_F

CAACTGAATCTGGCGGCGATTGCATTCCCAGTACCAGAACTATGTGGCGCGTTCGGAAGGTGCTTCGGCGCTGGCGACGATCAATCCGCTGAAGACCACTGTTGAAGAGTCTCTGTCGCGTGGAATTGCTGGTACCAATATTAAAATTGGTACTGATCCATCTACTGCCACCGAAACATATGTTGGGGTTGCTGCGAATGCCAACAAGCTGGGCTTGATTGATGTAAAGATTGCTGATACTGGTGCGGGTGATATTACCTTTACCTTCCAGACTGGTACTTCTAGTCCCAAGAATGCTACTAAAGTTATCACTCTGAATCGTACTGCGGATGGGGTCTGGGCTTGTAAATCTACCCAGGATCCGATGTTCACTCCGAAAGA

>1980323_pilB_x5_pilB_R

CTGGATTAAGTCAGACCCCATCCGCAGTACGATTCAGAGTGATAACTTTAGTAGCATTCTTGGGACTAGAAGTACCAGTCTGGAAGGTAAAGGTAATATCACCCGCACCAGTATCAGCAATCTTTACATCAATCAAGCCCAGCTTGTTGGCATTCGCAGCAACCCCAACATATGTTTCGGTGGCAGTAGATGGATCAGTACCAATTTTAATATTGGTACCAGCAATTCCACGCGACAGAGACTCTTCAACAGTGGTCTTCAGCGGATTGATCGTCGCCAGCGCCGAAGCACCTTCCGAACGCGCCACATAGTTCTGGTACTGGGGAATGGCAATCGCCGCCAGGATACCGATGATCGCAACCACGATCATCAGTTCGA


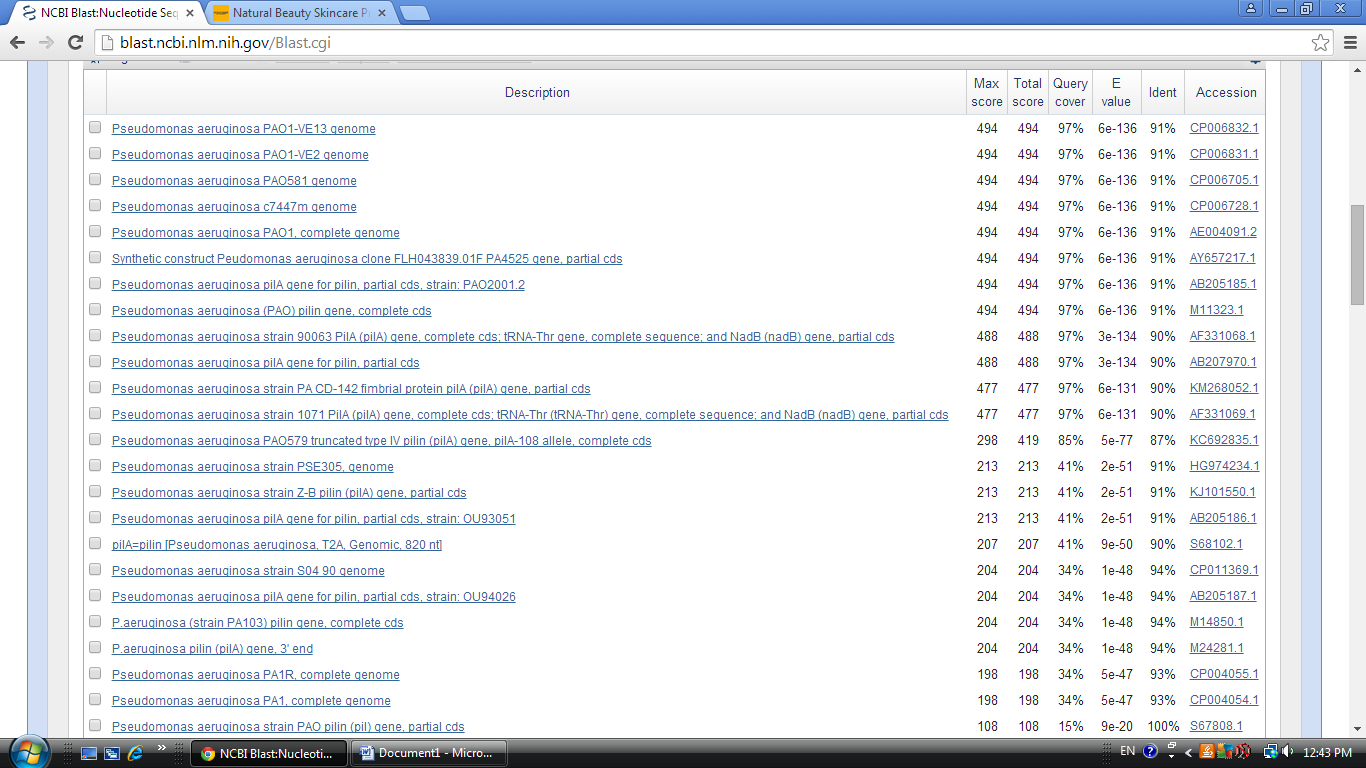

Supplement: Supplemental Information 5 [file peerj-07-6217-s005.docx]
